# Preparation and Synthetic Applications of Five-to-Seven-Membered Cyclic α-Diazo Monocarbonyl Compounds

**DOI:** 10.3390/molecules27062030

**Published:** 2022-03-21

**Authors:** Daniil Zhukovsky, Dmitry Dar’in, Olga Bakulina, Mikhail Krasavin

**Affiliations:** 1 Research & Development Department, BratskChemSyntez LLC, PharmaSyntez Company, 5A/1 Kommunalnaya St., 665717 Bratsk, Russia; jukouskij@mail.ru; 2 Institute of Chemistry, Saint Petersburg State University, 199034 Saint Petersburg, Russia; o.bakulina@spbu.ru; 3 Immanuel Kant Baltic Federal University, 236041 Kaliningrad, Russia

**Keywords:** α-diazo ketones, α-diazo lactones, α-diazo lactams, preparation, synthetic applications

## Abstract

The reactivity of cyclic α-diazo monocarbonyl compounds differs from that of their acyclic counterparts. In this review, we summarize the current literature available on the synthesis and synthetic applications of three major classes of cyclic α-diazo monocarbonyl compounds: α-diazo ketones, α-diazo lactones and α-diazo lactams.

## 1. Introduction

α-Diazocarbonyl compounds are distinctly versatile reagents for organic synthesis, capable of a wide range of synthetic transformations [1,2,3,4]. However, in contrast to more common cyclic 2-diazo-1,3-dicarbonyl compounds, five-to-seven-membered cyclic α-diazo monocarbonyl compounds (including α-diazo ketones, α-diazo lactones and α-diazo lactams) remain noticeably underexplored, both in terms of preparative methods to access them and their utility as reagents for organic synthesis. Such compounds (as well as carbene species generated therefrom) are expected to be more reactive compared to their acyclic counterparts, due to ring strain that causes, in turn, weaker conjugation of the diazo function (or the resulting carbene center) to the carbonyl group [5]. Higher reactivity may also be the reason for the lower chemical stability of α-diazo monocarbonyl compounds, which consequently hampers their preparation and use in organic synthesis. However, the range of preparative methods and synthetic applications enabling access to and the practical use of α-diazo monocarbonyl compounds has been expanding. In this review, we aim to summarize the current developments in the field of cyclic α-diazo monocarbonyl compounds, including α-diazo-γ-butyrolactams that were recently introduced and developed as reagents for organic synthesis in our laboratories.

## 2. Cyclic α-Diazo Ketoness

### 2.1. Methods of Synthesis of Cyclic α-Diazo Ketones

**Scheme 1:**
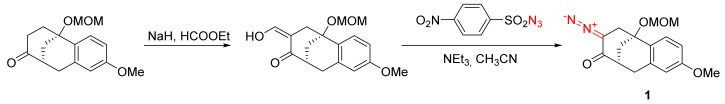
Example of deformylative diazo transfer in the synthesis of bicyclic α-diazo ketone **1**.

In the majority of cases, cyclic α-diazo ketones are synthesized using the general method of diazo transfer. As the acidity of the methylene group in the α-position to the carbonyl group is relatively low, the diazo transfer reaction requires fairly forceful conditions, which would lead to the other methylene group also being affected in the reaction. Therefore, to achieve a selective mono transfer of the diazo function, the ketones are preliminarily activated by formylation, and only such a monoformylated substrate is subjected to deformylative diazo transfer conditions [6,7,8,9]. This is illustrated by the preparation of mono-diazo bicyclic ketone derivative **1** (Scheme 1).

**Scheme 2:**
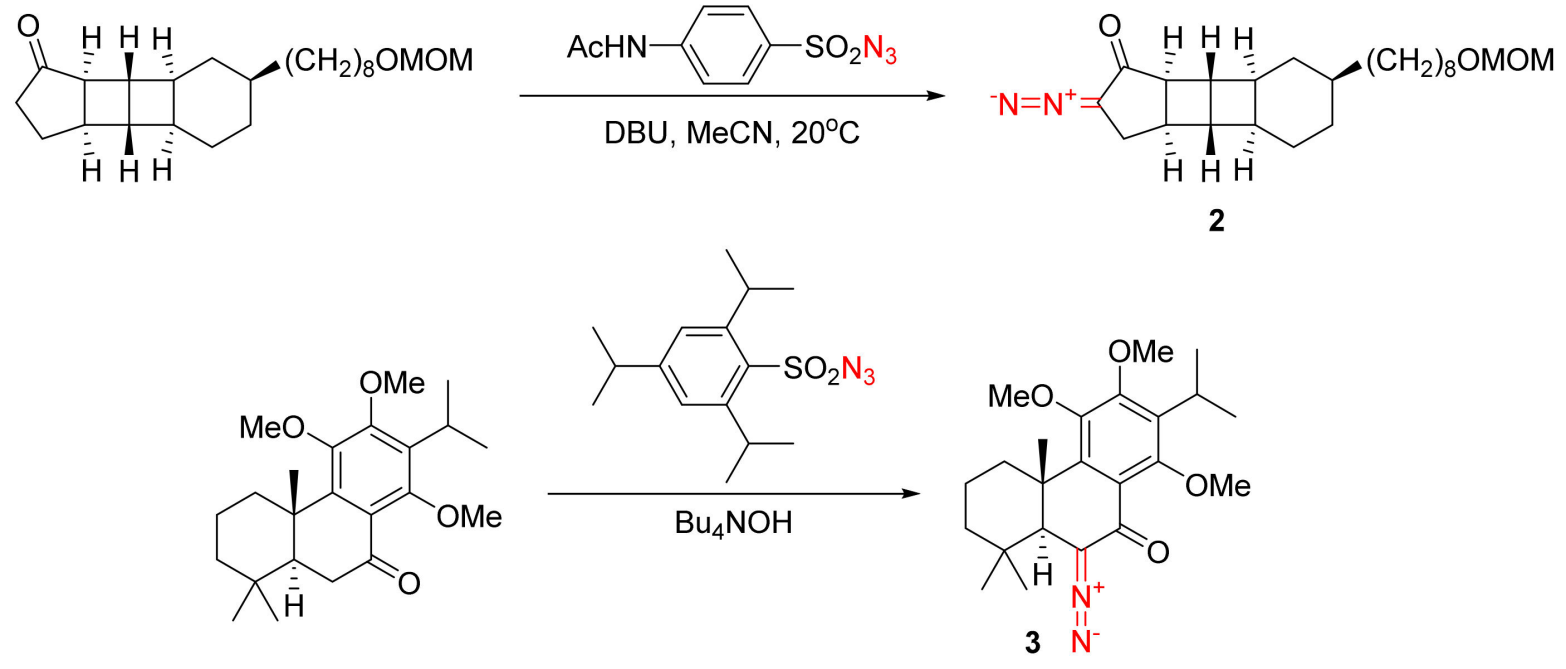
Examples of direct diazo transfer reactions not requiring pre-formylation.

In some cases, however, when the target molecule does not have a nearby methylene or methyne group with similar acidity to the target methylene group, a direct diazo transfer becomes possible, as illustrated by the synthesis of polycyclic α-diazo ketones **2** and **3** (Scheme 2) [10,11,12,13].

**Scheme 3:**
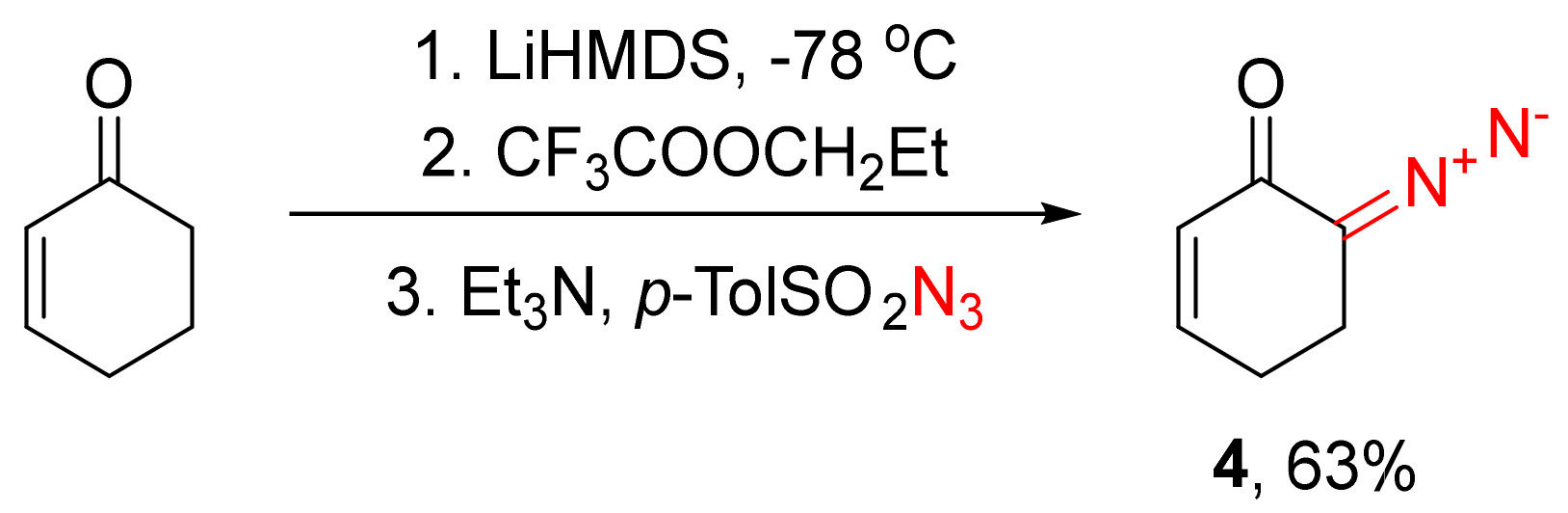
Synthesis of diazo ketone **4** via trifluoroacetylation.

A standalone case is when a diazo transfer is employed to prepare β,γ-unsaturated α-diazo cyclic ketones, such as α-diazo cyclohex-β,γ-enones. The lowered acidity of the α-methylene group, along with the presence of the reactive double bond, mandate that the substrate is first α-trifluoroacetylated in the presence of a strong base at a low temperature. Thereupon, the de-trifluoroacetylative diazo transfer can already occur. This is illustrated by the synthesis of α-diazo δ,ε-cyclohexenone **4** (Scheme 3) [14,15,16].

**Scheme 4:**
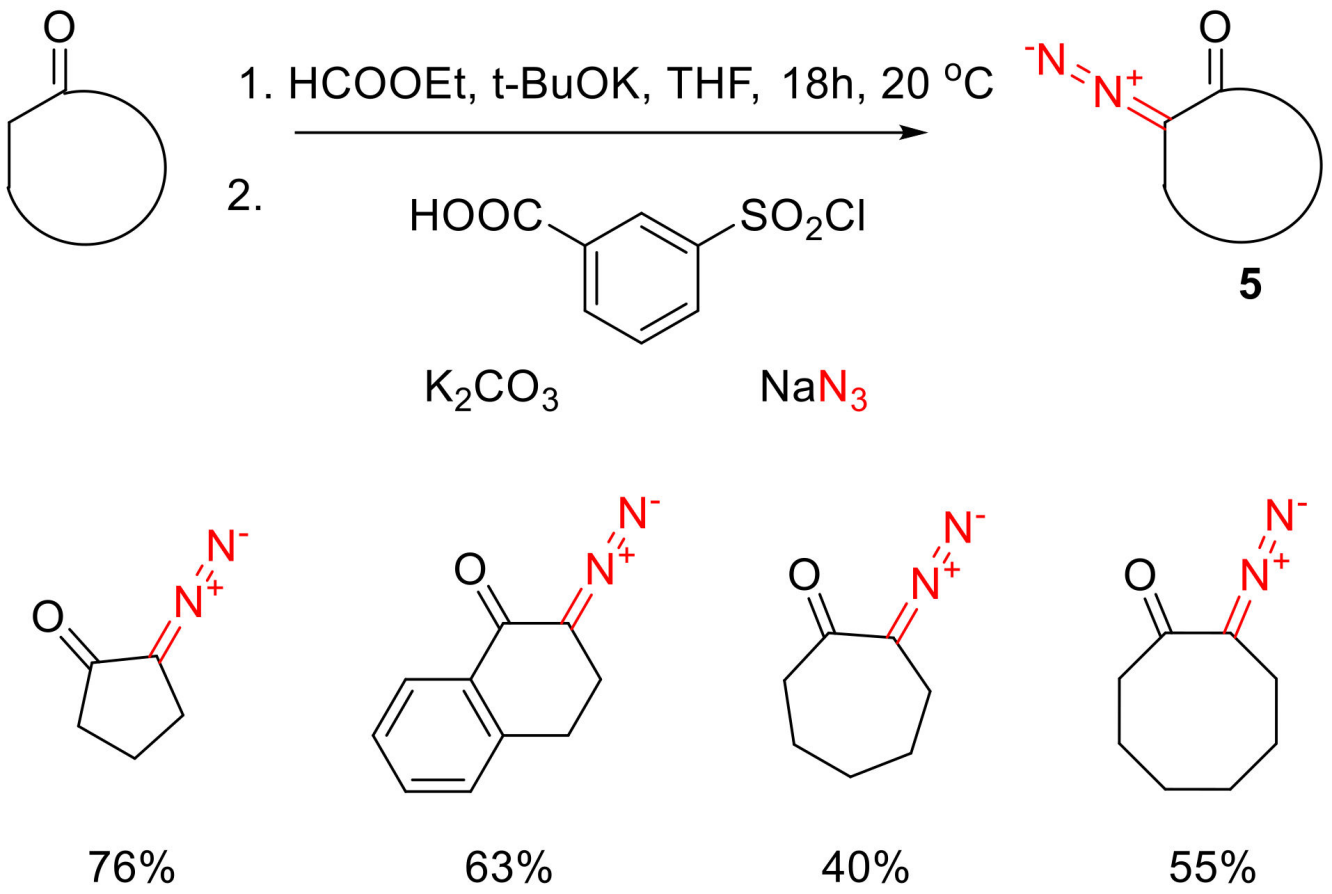
The ‘SAFE’ method of preparing cyclic α-diazo ketones **5**.

The main drawback of many diazo transfer reactions is the formation of an equivalent amount of sulfonamide, which requires the target product to be chromatographically purified. This drawback can be conveniently alleviated by the recently described modification of the diazo transfer reaction termed ‘sulfonyl-azide-free’ (SAFE), which allows, among other things, the synthesizing of cyclic α-diazo ketones. The key to this method is the use of water-soluble 3-(chlorosulfonyl)benzoic acid as the precursor to the respective sulfonyl azide. The latter is formed in situ under the influence of sodium azide in a weakly alkaline aqueous solution. To the solution, thus formed, a formylated cyclic ketone is added and, when the reaction is over, the target α-diazo ketone is simply extracted with chloroform while the unwanted sulfonamide remains in the water. This method turns out to be applicable to the preparation of high-purity five-to-eight-membered cyclic ketones **5** in good yields, the only surprising exception being cyclohexanone (Scheme 4) [17].

**Scheme 5:**
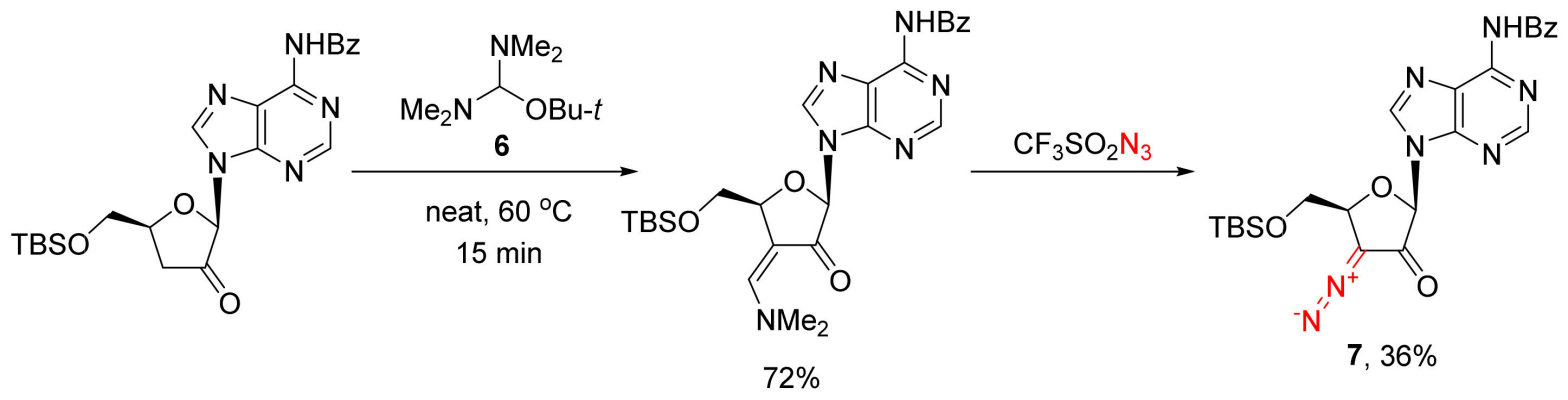
The use of the Bredereck’s reagent (**6**) in the activation of sensitive substrates.

The major drawback of synthesizing α-diazo ketones using the diazo transfer reaction is the need to use a strong base. If the use of such bases must be avoided for some reason, a diazo transfer could involve, not a premade enol but rather, an enamine. Such enamines are easy to synthesize using the so-called Bredereck’s reagent (**6**) in reactions with the starting cyclic ketones. In this case, the use of a strong base is required at neither the enamine synthesis stage nor at the diazo transfer step, as illustrated, for example, by the synthesis of a rather sensitive nucleobase **7** derivative (Scheme 5) [18,19].

**Scheme 6:**
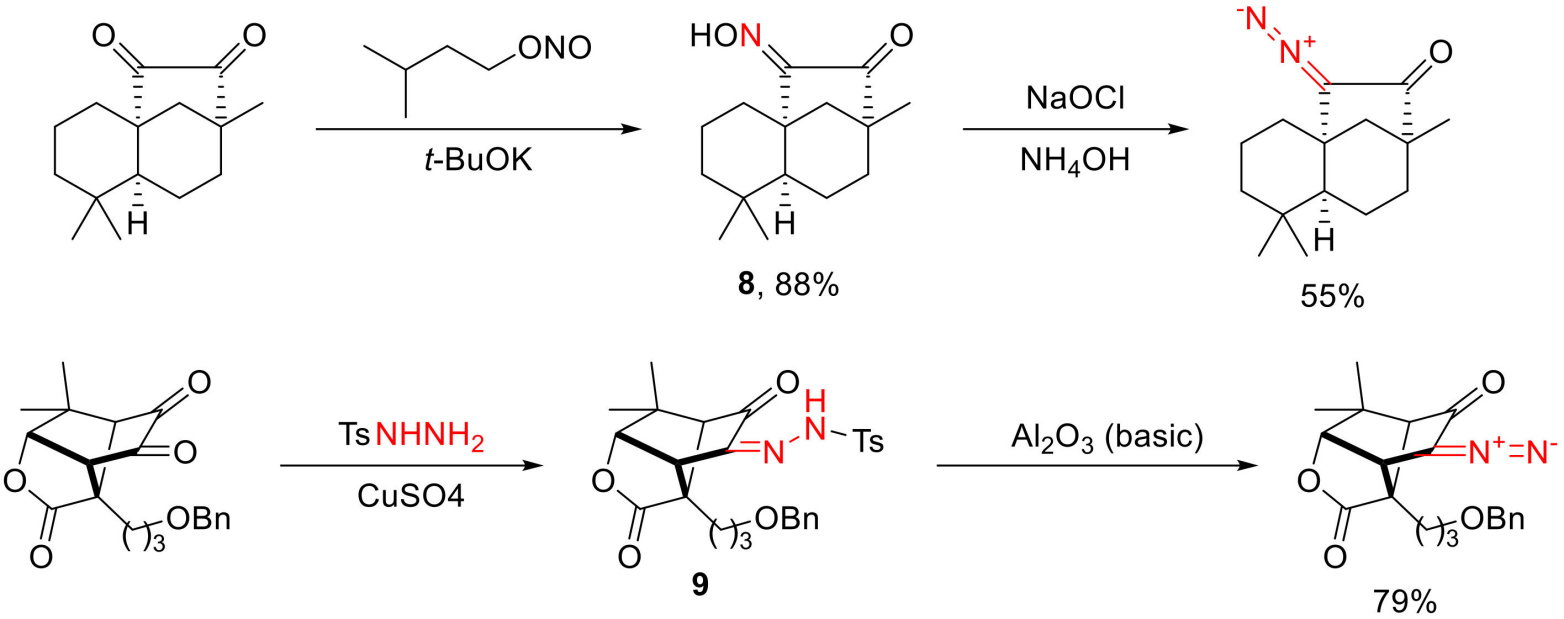
Examples of α-diazo ketone synthesis via oxime (**8**) and tosyl hydrazone (**9**) fragmentation.

In one case, one of the two carbonyl groups is turned into an oxime (such as **8**) with treatment with isoamyl nitrite in a basic medium, and the resulting oxime is oxidized to a diazo group [20]. Alternatively, one of the carbonyl groups can be transformed into a tosyl hydrazone, and the resulting hydrazone (such as **9**) is fragmented with a base to give the target α-diazo ketone (Scheme 6) [21,22,23].

**Scheme 7:**
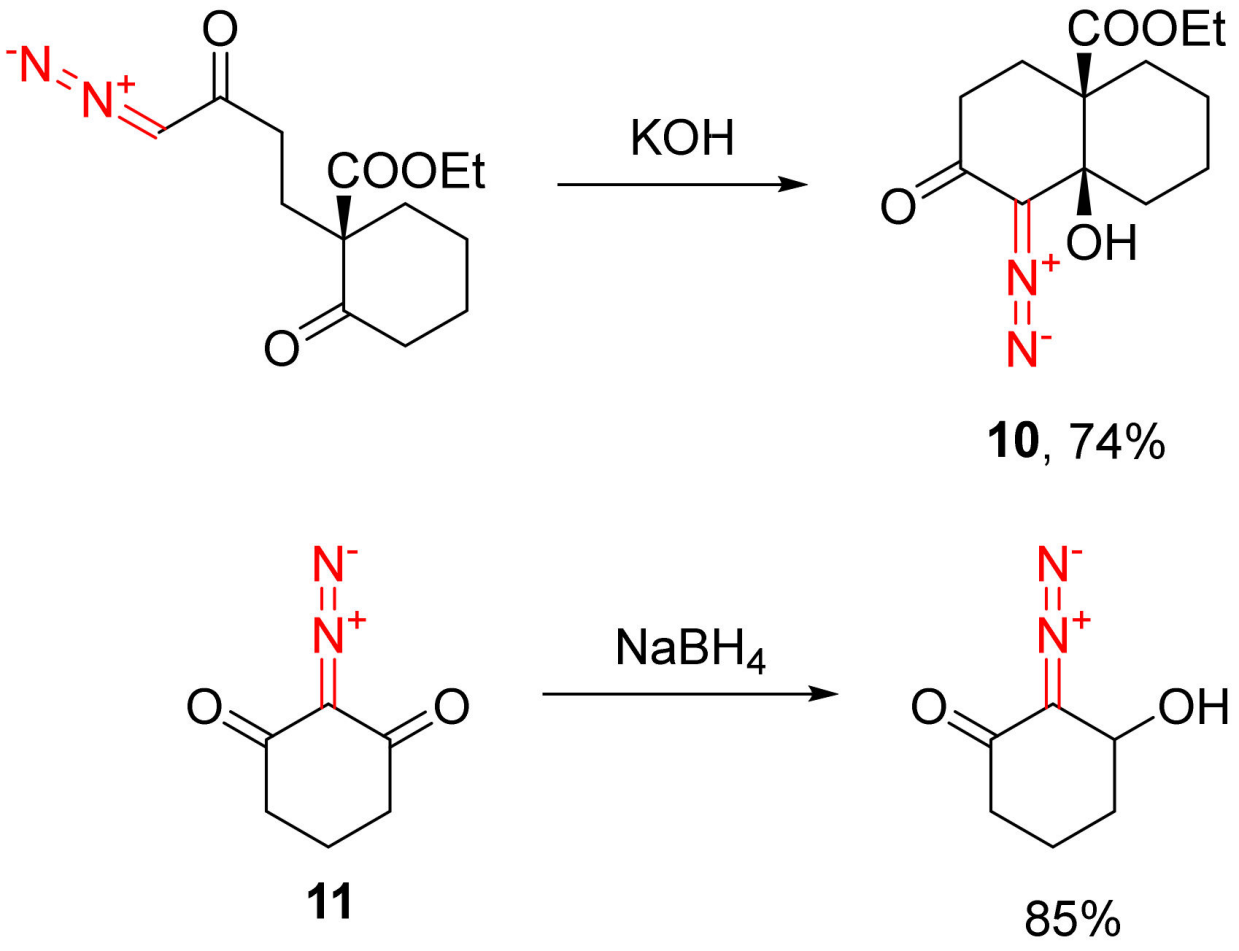
Approaches to the synthesis of β-hydroxy-α-diazo cyclic ketones.

A separate case is represented by the synthesis of β-hydroxy-α-diazo cyclic ketones—an important class of diazo compounds. The most common way to access such building blocks is with a base-promoted intramolecular addition of a terminal diazo ketone to another carbonyl group (as shown for the preparation of **10**) [24,25,26]. More simple derivatives of this class can be accessed from the respective diazo dicarbonyl compounds (such as **11**) by desymmetrizing the reduction of one of the carbonyl groups (Scheme 7) [27].

### 2.2. The Wolff Rearrangement of Cyclic α-Diazo Ketones

Despite the more than fifty-year history of studying the reactions of ketenes resulting from the decomposition of cyclic diazo monocarbonyl compounds, these reactions still find a wide utility in organic synthesis. To a large extent, this has to do with the utility of the Wolff rearrangement, and the subsequent addition of nucleophiles to the ketene that results, for the synthesis of cyclopentane and cyclobutane carboxylic acids for the needs of total synthesis [12].

**Scheme 8:**
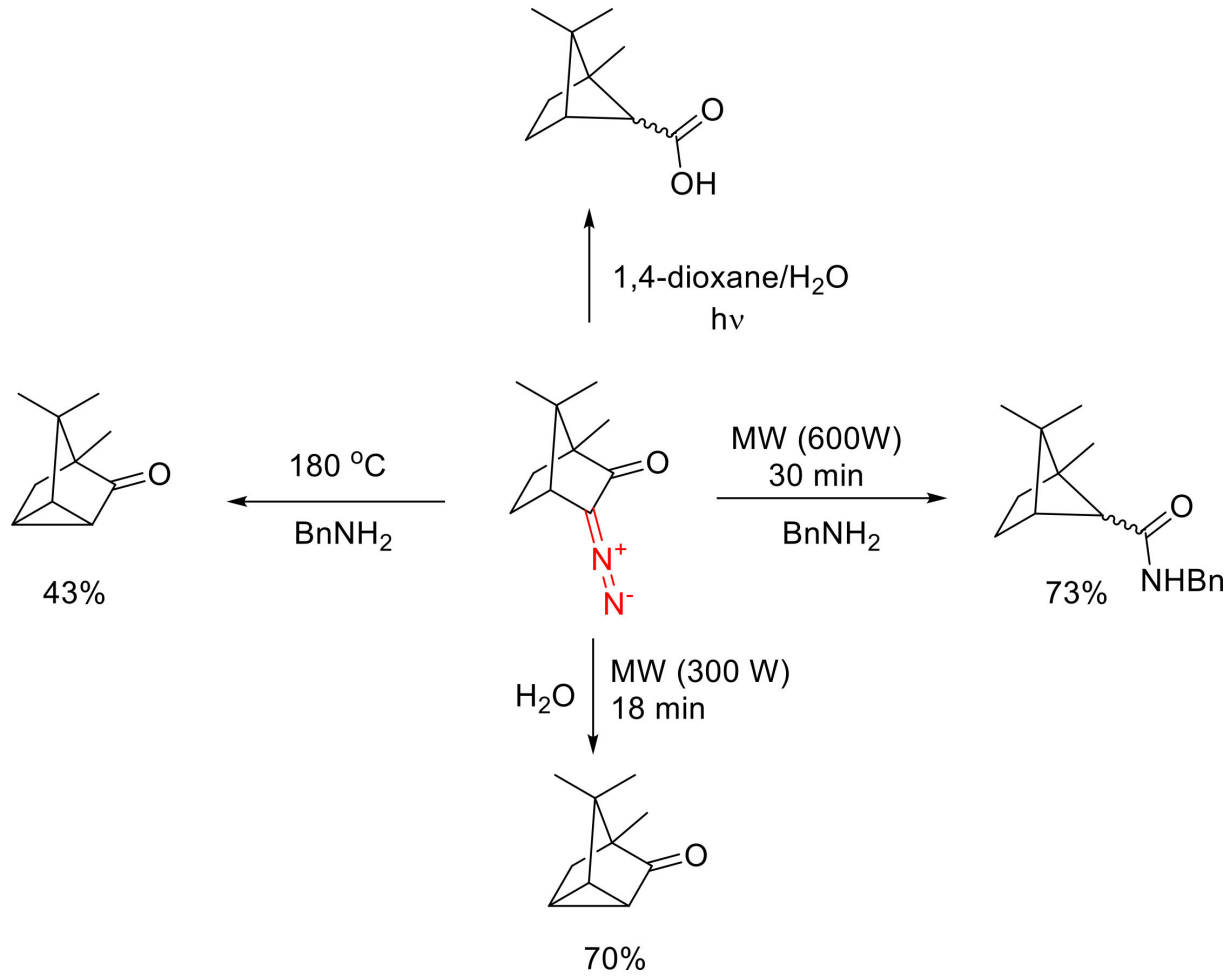
Various transformations of 3-diazo camphor under microwave irradiation and conventional heating.

Notably, the α-carbonyl carbenes formed with the decomposition of cyclic diazo monocarbonyl compounds are more inclined to undergo the Wolff rearrangement than their acyclic counterparts, which is supported by quantum chemical calculations [28]. This has to do with the fact that, in acyclic carbenes, the unshared electron pair is stabilized due to the interaction with the orthogonally oriented π* orbital of the carbonyl group. Because cyclic diazo carbonyl compounds are conformationally fixed, such orthogonal orientation of the unshared pair of the resulting carbene and the carbonyl group cannot be achieved. Hence, the latter type of carbenes has a higher reactivity. For cyclic diazo ketones, the Wolff rearrangement has been shown to be more facile under microwave irradiation than under thermal conditions [29]. For the Wolff rearrangement under microwave irradiation, the choice of the solvent plays a crucial role. For instance, 3-diazo camphor microwave-irradiated in benzylamine gives an excellent yield of ketene adducts. Under thermal conditions, the sole product is that of a formal intramolecular insertion into the C-H bond. However, attempts to bring about the Wolff rearrangement in an aqueous medium does not lead to the formation of the respective carboxylic acid, even under microwave irradiation, since the intramolecular C-H insertion product is the sole one in this case. This has to do with the high absorption of the microwave irradiation by water (due to its high dipole moment) and the resulting heating of the reaction mixture. The said carboxylic acid can only be obtained from 3-diazo camphor under photochemical conditions in 1,4-dioxane (Scheme 8) [29].

Notably, a nucleophile addition to cyclic ketenes tends to proceed with endo-selectivity [30,31,32].

**Scheme 9:**
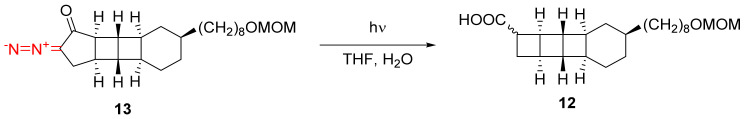
The key step in the synthesis of *ent*-[3]ladderanol.

The Wolff rearrangement of α-diazo cyclopentanones has found broad applications in organic synthesis. In aqueous tetrahydrofuran or 1,4-dioxane, free cyclobutanecarboxylic acids can be obtained [11,33]. For example, in the total synthesis of *ent*-[3]-ladderanol, the third cyclobutene ring is introduced in key intermediate **12** through the Wolff rearrangement of the fairly complex α-diazo cyclopentane precursor **13** (Scheme 9). The low diastereoselectivity of the process does not present an issue because the carboxylic acid group is removed in the next step under acidic conditions [11].

**Scheme 10:**
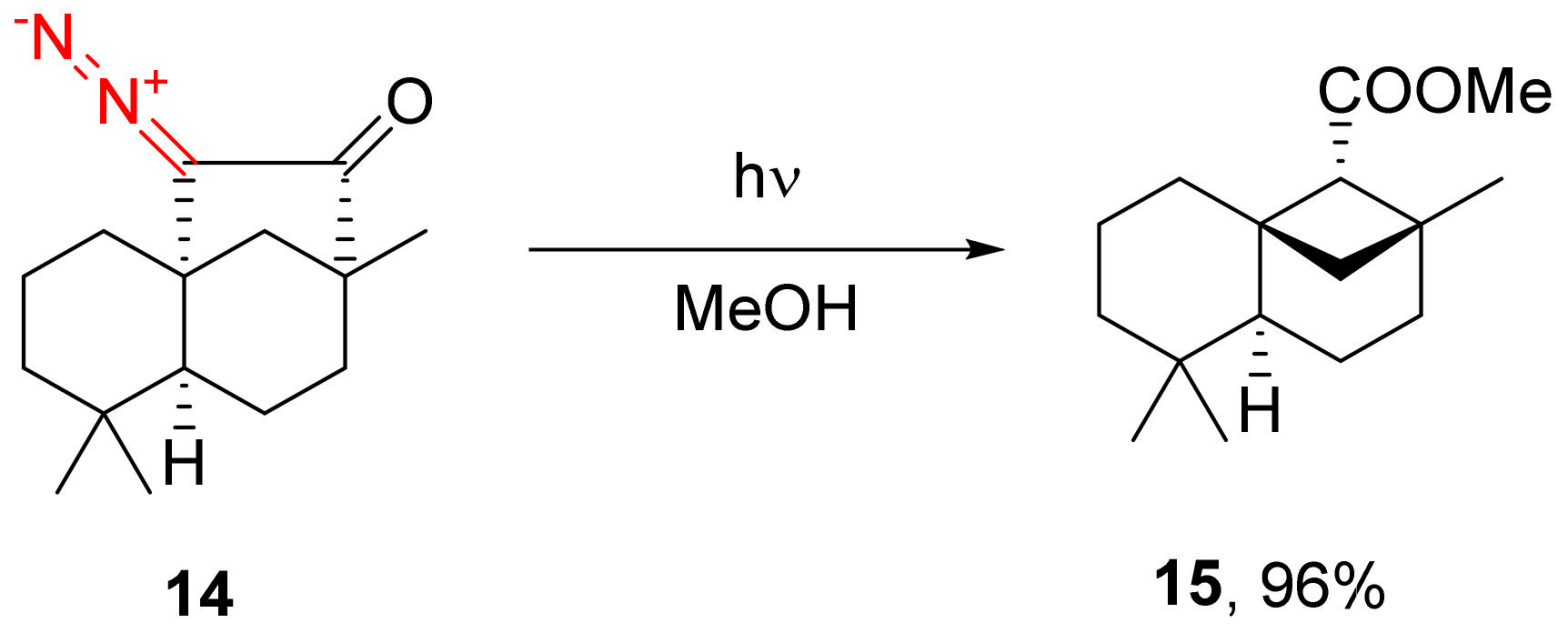
Example of photolytic decomposition of α-diazo cyclopentanone with diastereoselective formation of the Wolff rearrangement product.

An alkoxycyclobutane carboxylate fragment can easily be obtained via the photochemical decomposition of the α-diazo cyclopentanone moiety in **14** in an alcoholic solution [34,35]. In some cases, the respective product **15** can be obtained diastereoselectively (Scheme 10) [7,20,36].

**Scheme 11:**
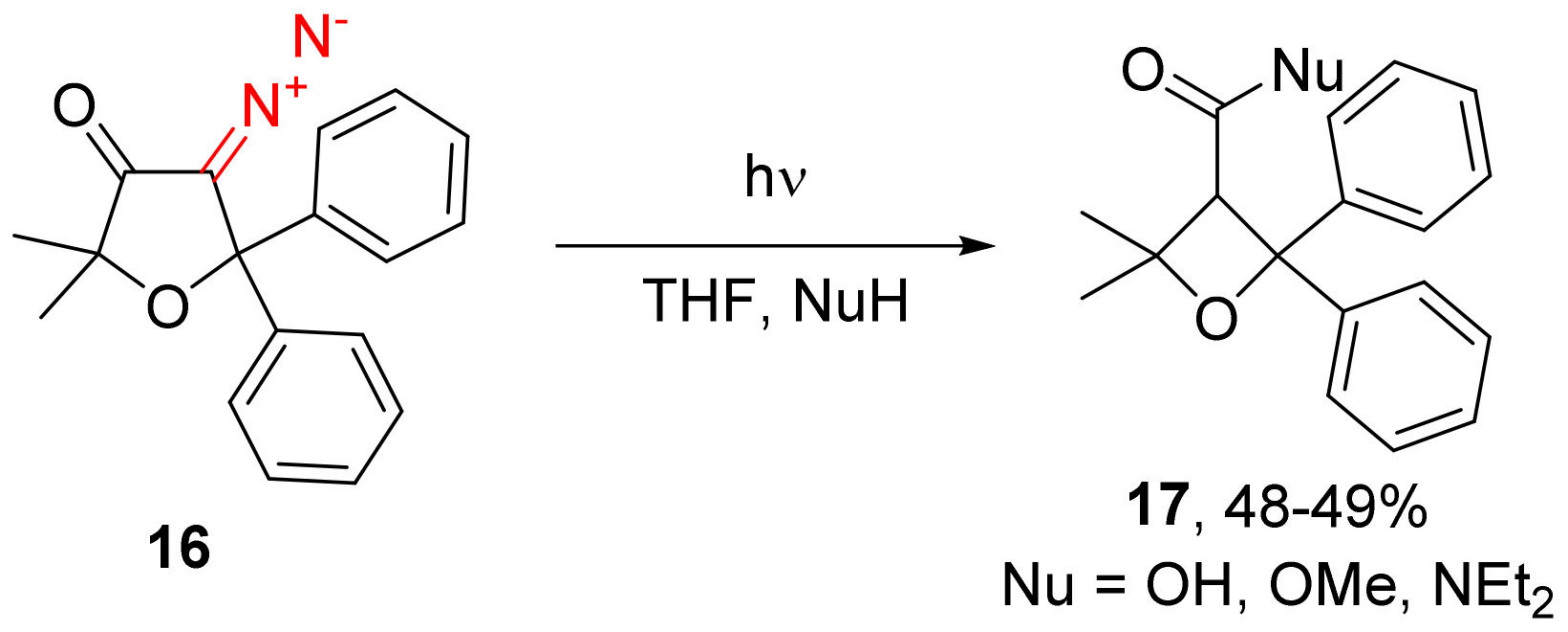
Photolysis of 4-diazodihydrofuran-3(2*H*)-ones in the presence of various nucleophiles.

Not only α-diazo cyclopentanones can undergo the Wolff rearrangement with a subsequent nucleophile addition. Their heterocyclic analogs can undergo a similar transformation [37]. For instance, the photochemical decomposition of 4-diazodihydrofuran-3(2*H*)-ones, such as **16**, in tetrahydrofuran in the presence of various nucleophiles, delivers derivatives of oxetan-3-carboxylic acids **17** in modest yields and moderate diastereoselectivity (Scheme 11) [18]. Unfortunately, the reported reaction is accompanied by numerous side-products, including products of the tetrahydrofuran addition, 1,2-migration and diazo function reduction [38,39].

**Scheme 12:**
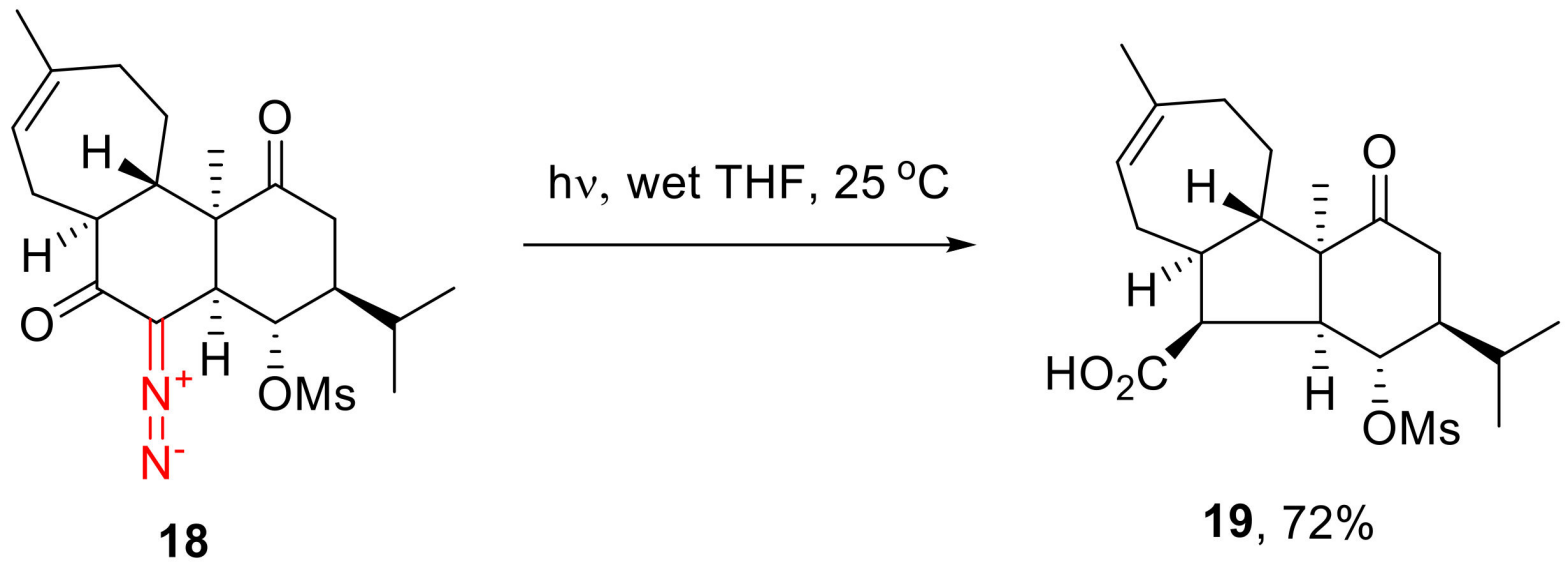
The Wolff rearrangement involving six-membered α-diazo carbonyl compounds.

The Wolff rearrangement of carbonyl carbenes formed from α-diazo cyclohexanones has gained prominence in organic synthesis; however, it is less widespread compared to their five-membered counterparts. For instance, the photochemical decomposition of diazo compound **18** in wet tetrahydrofuran delivers the respective cyclopentane carboxylic acids **19** (Scheme 12) [40].

**Scheme 13:**
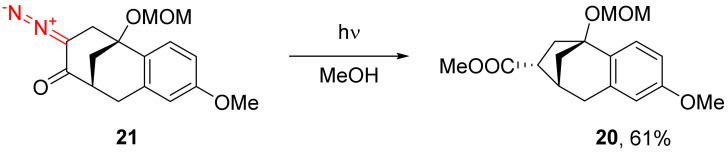
Photolysis of bicyclic diazo compound **21** in methanol.

Alkyl cyclopentane carboxylate **20** is obtained in the course of the photochemical [30,41] decomposition of bicyclic diazocyclohexane **21** in the presence of methanol, usually employed as a solvent (Scheme 13).

**Scheme 14:**
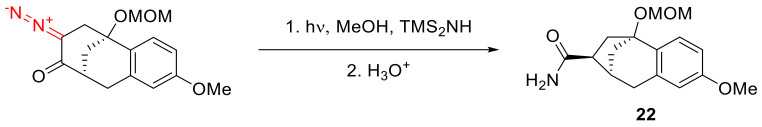
Using hexamethyldisylazane as a nucleophile in the Wolff rearrangement.

Using hexamethyldisylazane as a nucleophile with subsequent acidic hydrolysis allows the obtaining of primary amide **22** (Scheme 14) [42].

Notably, the decomposition of α-diazo cyclohexanones in the presence of nucleophiles typically proceeds diastereoselectively [10,13].

**Scheme 15:**
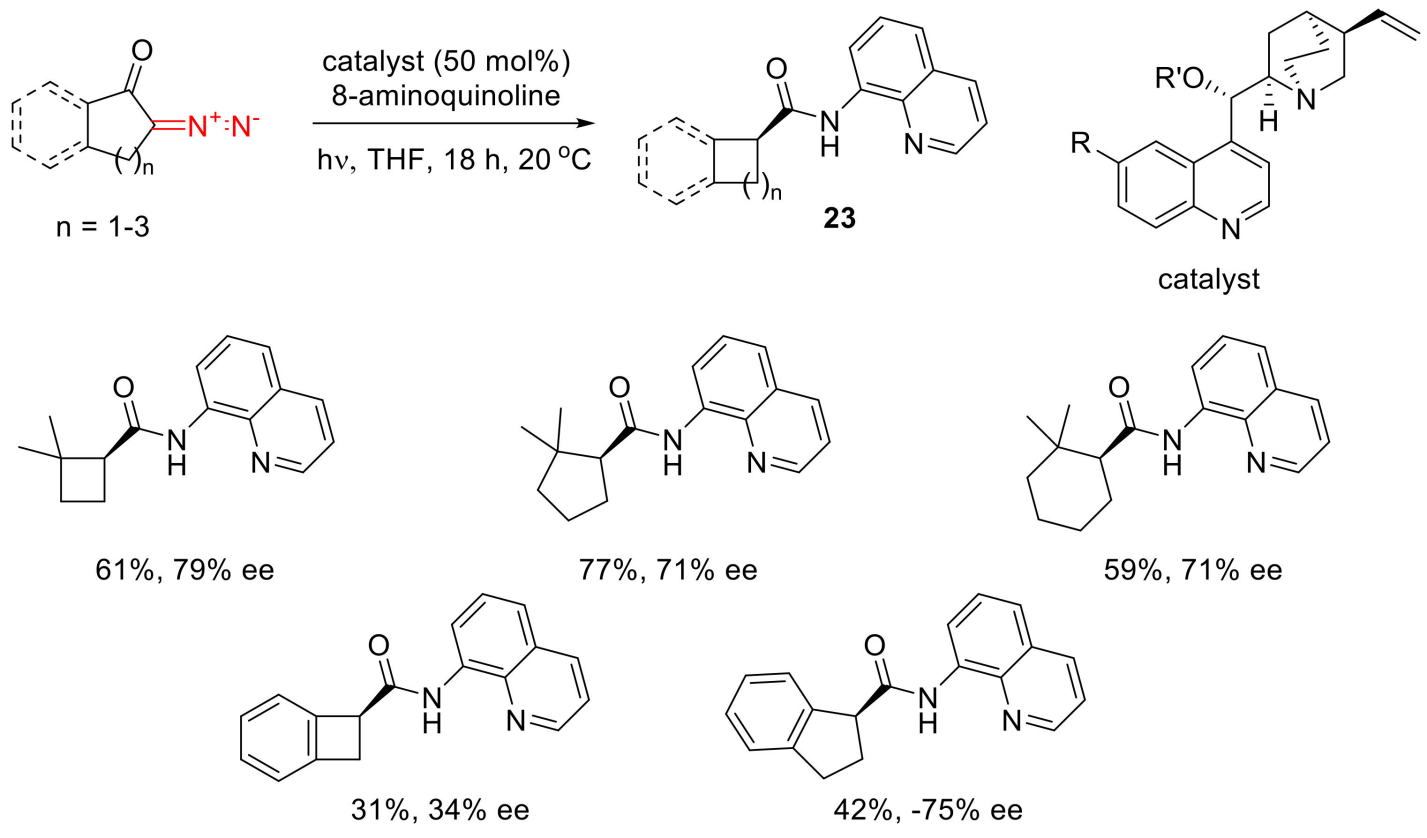
Examples of an enantioselective nucleophile addition to ketenes.

Recently, an enantioselective version of a nucleophile addition to ketenes has been reported. The resulting 8-acylamino isoquinolines **23** were obtained with *ee* values exceeding 70% (Scheme 15) [43].

**Scheme 16:**
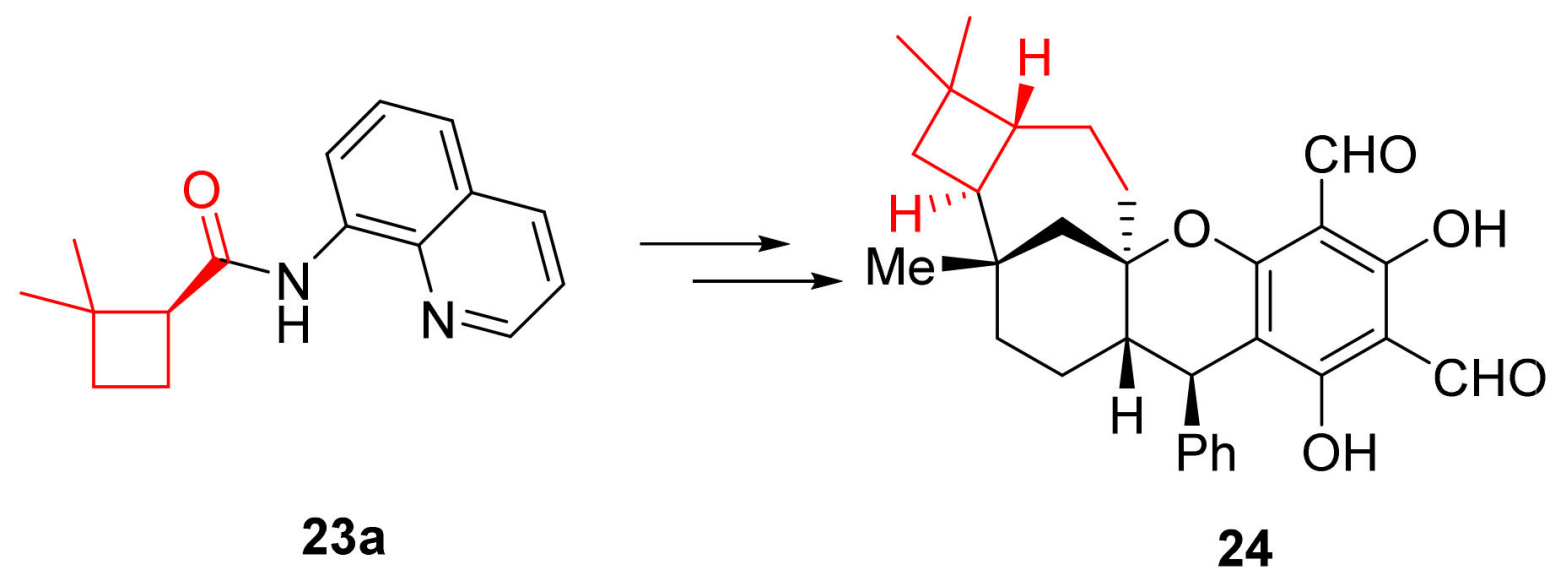
Enantioselective synthesis of (+)-psiguadial B (**24**).

One of the enantioenriched building blocks obtained that way (**23a**) was employed in the total synthesis of (+)-psiguadial B (**24**) (Scheme 16).

**Scheme 17:**
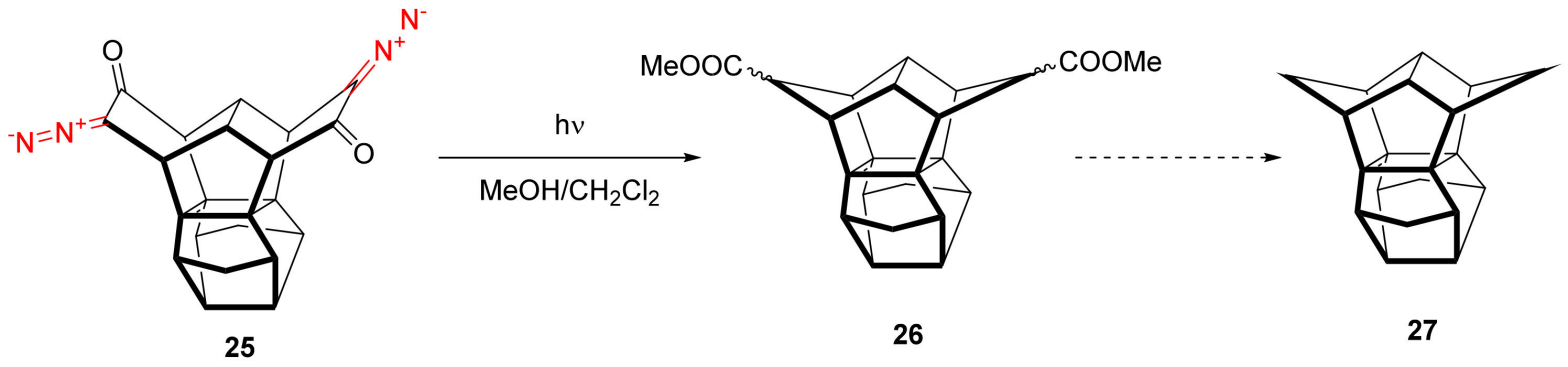
Bis-diazo compound photolysis in the synthesis of iso[1.1.1.1]pagodane **27**.

The decomposition of cyclic diazo ketones with a subsequent Wolff rearrangement can be employed, not only in total synthesis, but also for the construction of polycyclic cage structures. For instance, the photochemical transformation of bis-diazo cyclohexanone **25** into bis-methyl cyclopentanoate **26** is the key step in the synthesis of iso[1.1.1.1]pagodane **27** (Scheme 17) [44].

**Scheme 18:**
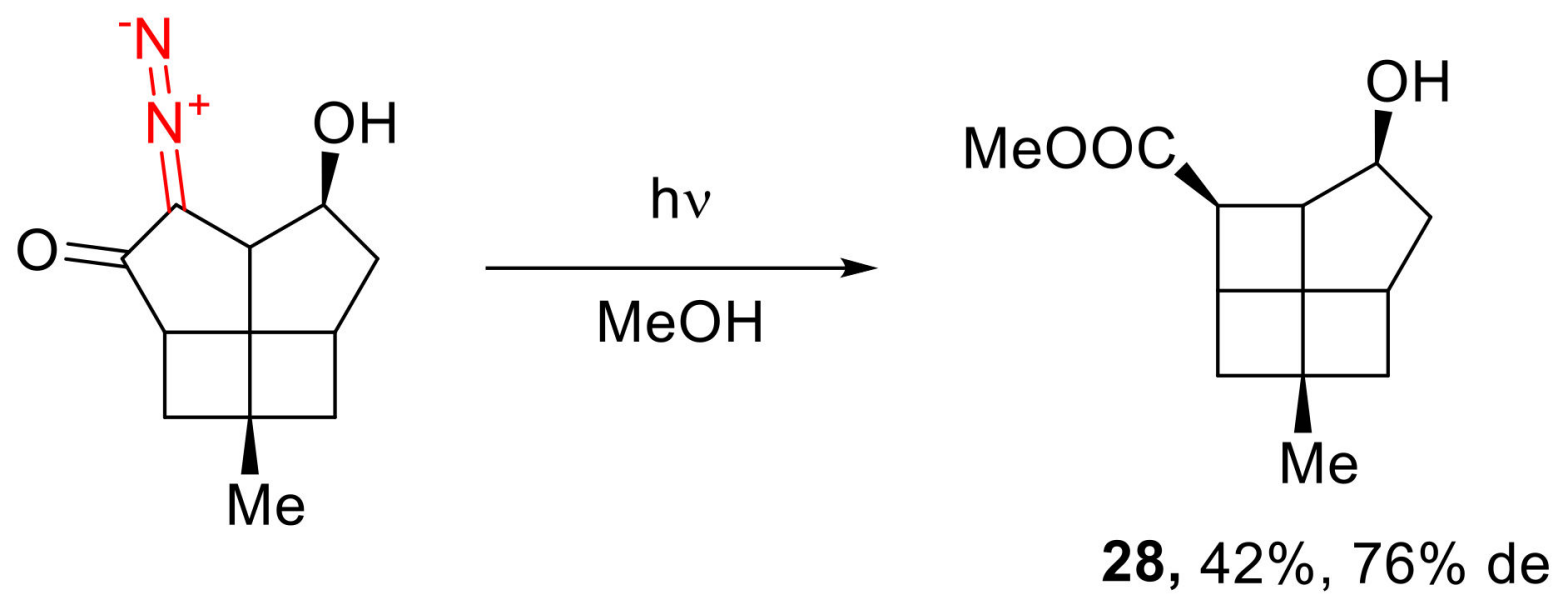
Synthesis of [4.4.4.5]fenestrane skeleton.

The photolysis of the α-diazo cyclopentanone moiety in methanol was successfully employed toward the construction of [4.4.4.5]fenestrane skeleton (**28**) [40]. Other authors have managed to conduct that reaction diastereoselectively (Scheme 18) [8].

**Scheme 19:**
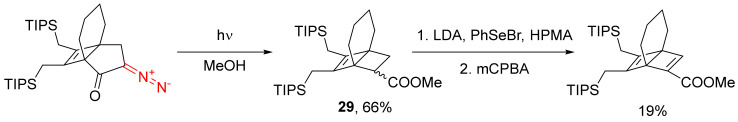
Synthesis of Dewar arene structures.

Another application of the photolysis of cyclic diazo monocarbonyl compounds is in the synthesis of the Dewar arenes. In this synthesis, the resulting bicyclohexenecarboxylic ester **29** is selenylated and oxidized with *m*-CPBA (Scheme 19) [35,45].

**Scheme 20:**
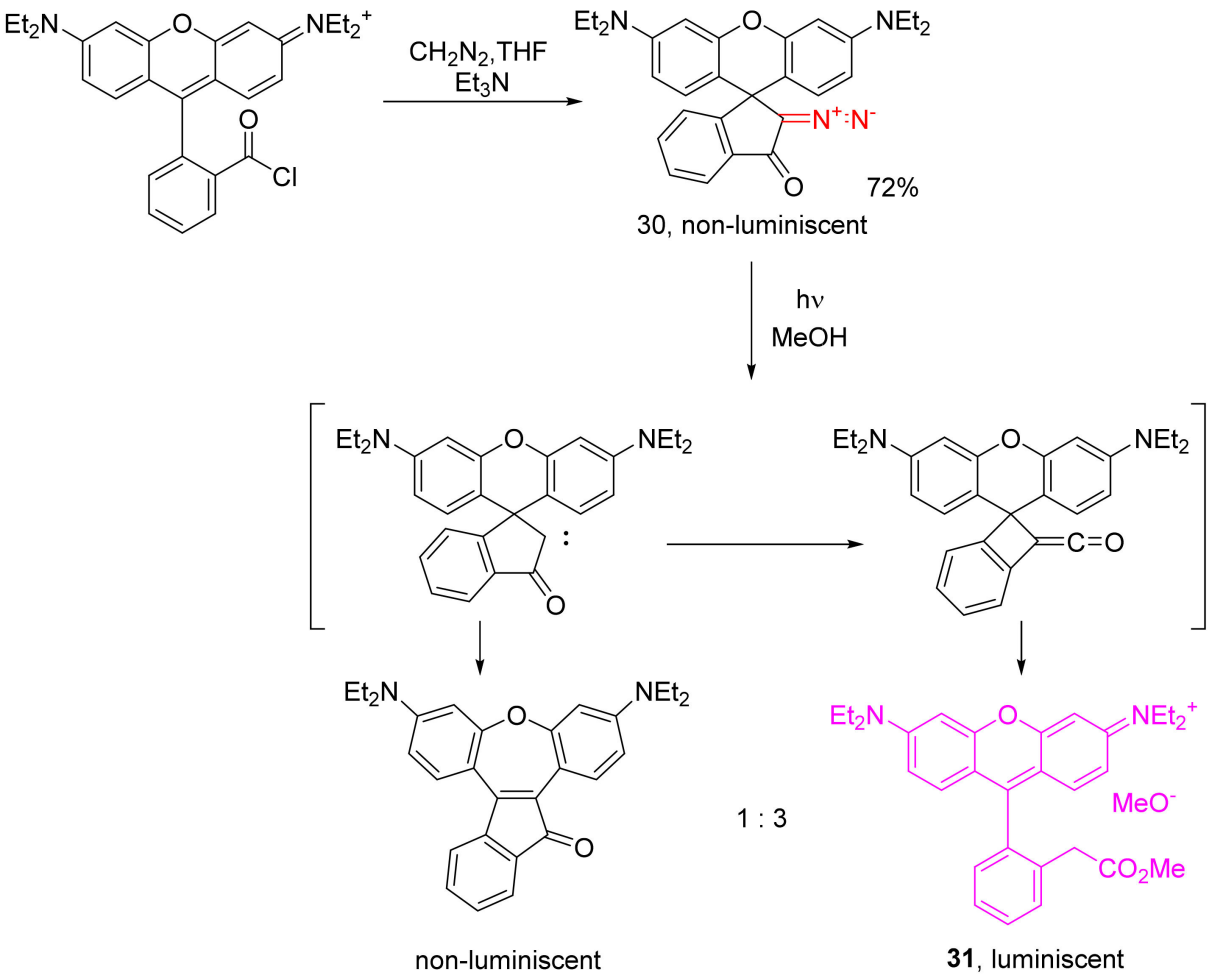
Photochemical production of strongly luminescent rhodamine B derivatives.

The reactions of cyclic ketenes with nucleophiles finds an interesting application in the dye chemistry. The diazo derivative of rhodamine obtained from the respective chloroanhydride is, itself, not luminescent, as it exists in the spiro form. However, its photochemical decomposition in methanol results in the formation of two products. The major product **31** results from the Wolff rearrangement and the addition of a methanol molecule, with a subsequent rupture of the cyclobutene cycle, in which an extended conjugate system is formed, and it demonstrates strong fluorescence. The minor product, resulting from a 1,2-aryl shift in carbonyl carbene, does not display luminescent properties (Scheme 20) [46].

The photoactivation of diazo rhodamines finds application in various areas of bioimaging. In nonactivated form, these dyes are not charged and, therefore, can cross the phospholipid membrane of the cell [46,47,48].

**Scheme 21:**
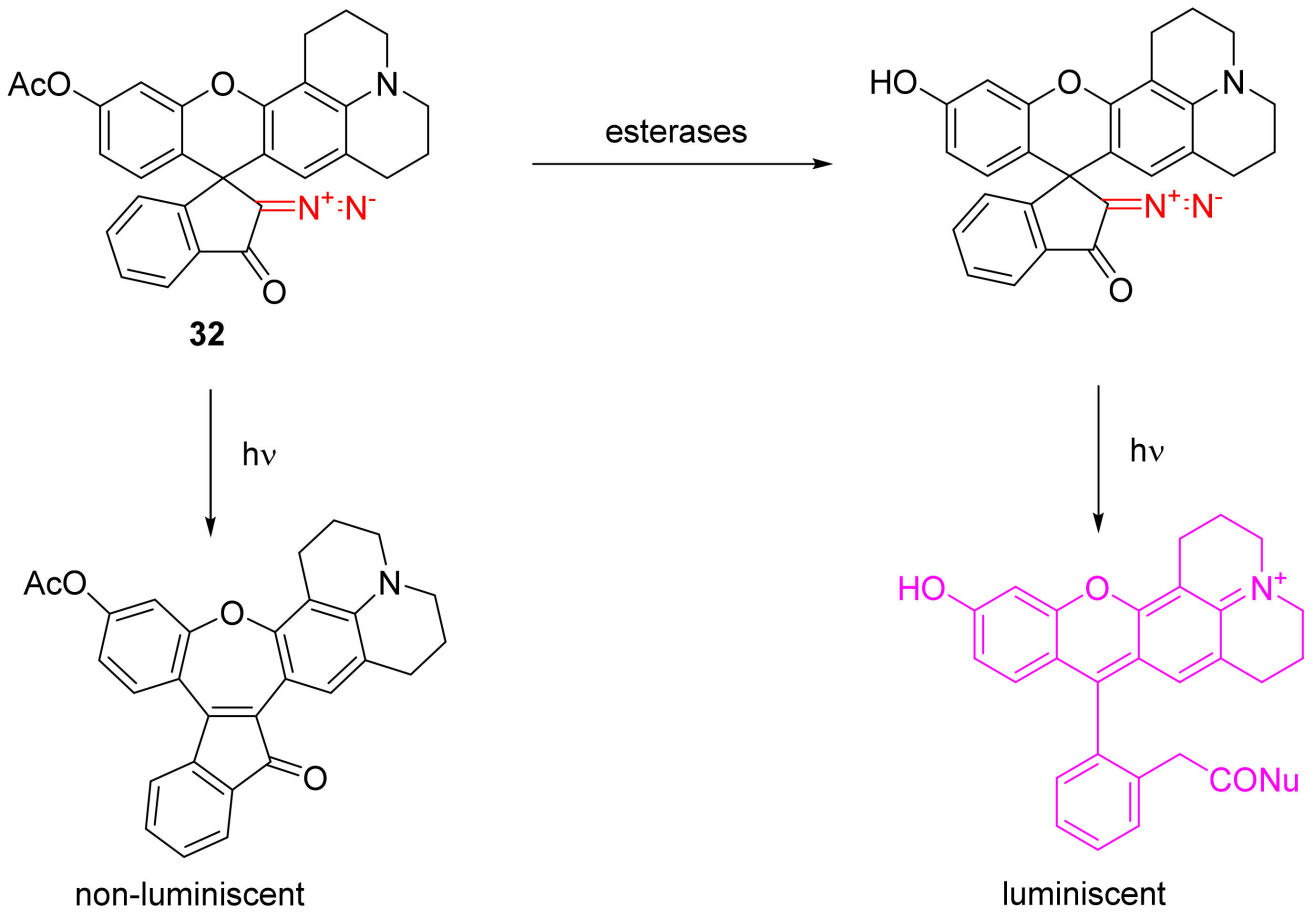
Rhodamine–fluoresceine sensor for esterases.

Particularly interesting is the mixed rhodamine–fluoresceine sensor **32** for esterases. The phenolic group in **32** is acylated which leads to a non-luminescing photo adduct. In the presence of esterases, **32** is de-acetylated and, upon photolysis and the addition of external nucleophiles, gives a strongly luminescent product (Scheme 21) [49].

**Scheme 22:**
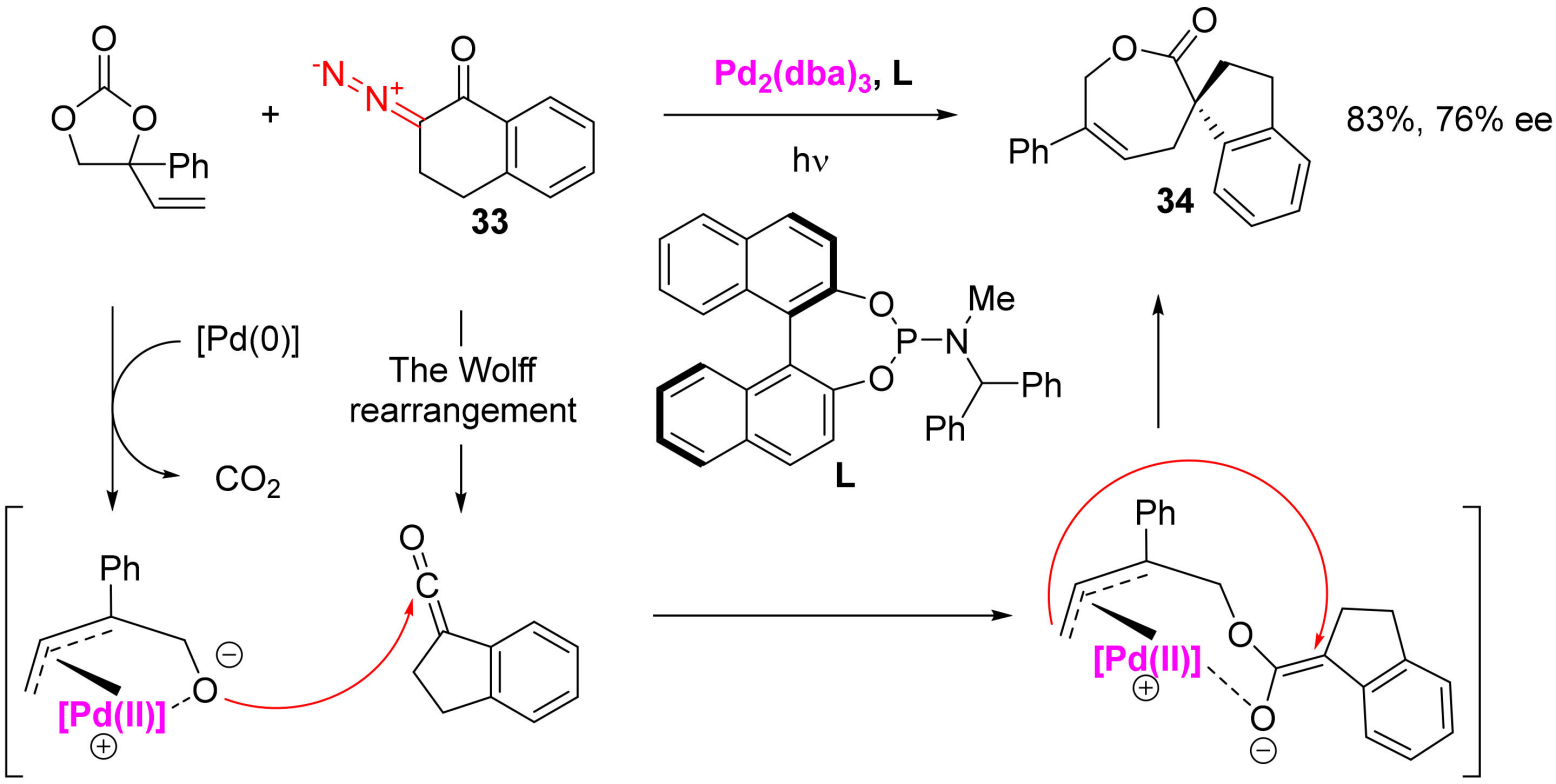
Enantioselective, Pd(II)-catalyzed formation of spirocyclic adduct **34**.

The ketene adduct with nucleophiles may turn out to be rather reactive itself and undergo various post-transformations. With the photochemical decomposition of β-diazo-α-tetralone **33** in the presence of vinyl ethylene carbonate and chiral palladium catalyst, spirocycle **34** is formed in high yield and *ee*. According to the proposed mechanism, the ketene interacts with the nucleophilic oxygen atom of alkoxy-η^3^-allyl ligand of Pd(II). The initial adduct, bidentately coordinated to Pd(II), undergoes intramolecular asymmetric allylic alkylation, which leads to the observed product **34** (Scheme 22). It is likely that β-diazo-α-indanone and its seven-membered analog can also undergo a similar transformation [50].

**Scheme 23:**
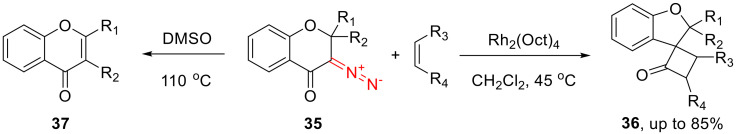
Cyclic ketenes in [2 + 2] cycloaddition reactions.

The Wolff-rearrangement-derived ketenes can interact with more than nucleophiles. For instance, ketenes derived from 3-diazochroman-4-one (**35**) can undergo [2 + 2] cycloaddition with various olefins delivering 6-oxospiro[3.4]octan-1-ones **36** [51]. The presence of the catalyst is necessary to suppress a competition 1,2-alkyl migration, which would lead to 4-benzopyrans **37**. Unfortunately, the reaction appears to be limited to 2,2-disubstituted 3-diazochoman-4-ones, as even monosubstituted ones predominantly undergo 1,2-hydride shift, and ketene is not formed (Scheme 23).

**Scheme 24:**
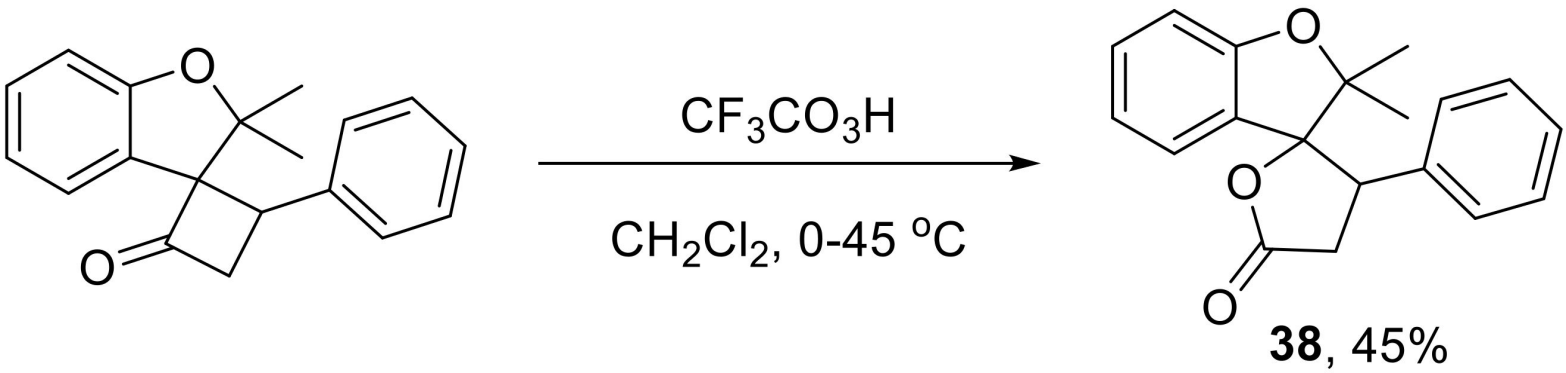
The Baeyer–Villiger oxidation of spirocyclic Wolff rearrangement products.

Spirocyclic compounds derived from the Wolff rearrangement can undergo a Baeyer–Villiger oxidation to give 1,7-dioxospiro[3.4]octan-1-ones **38**—an important building block for natural product synthesis (Scheme 24) [52,53].

### 2.3. Decomposition of Cyclic α-Diazo Ketones Followed by β-Migration

**Scheme 25:**
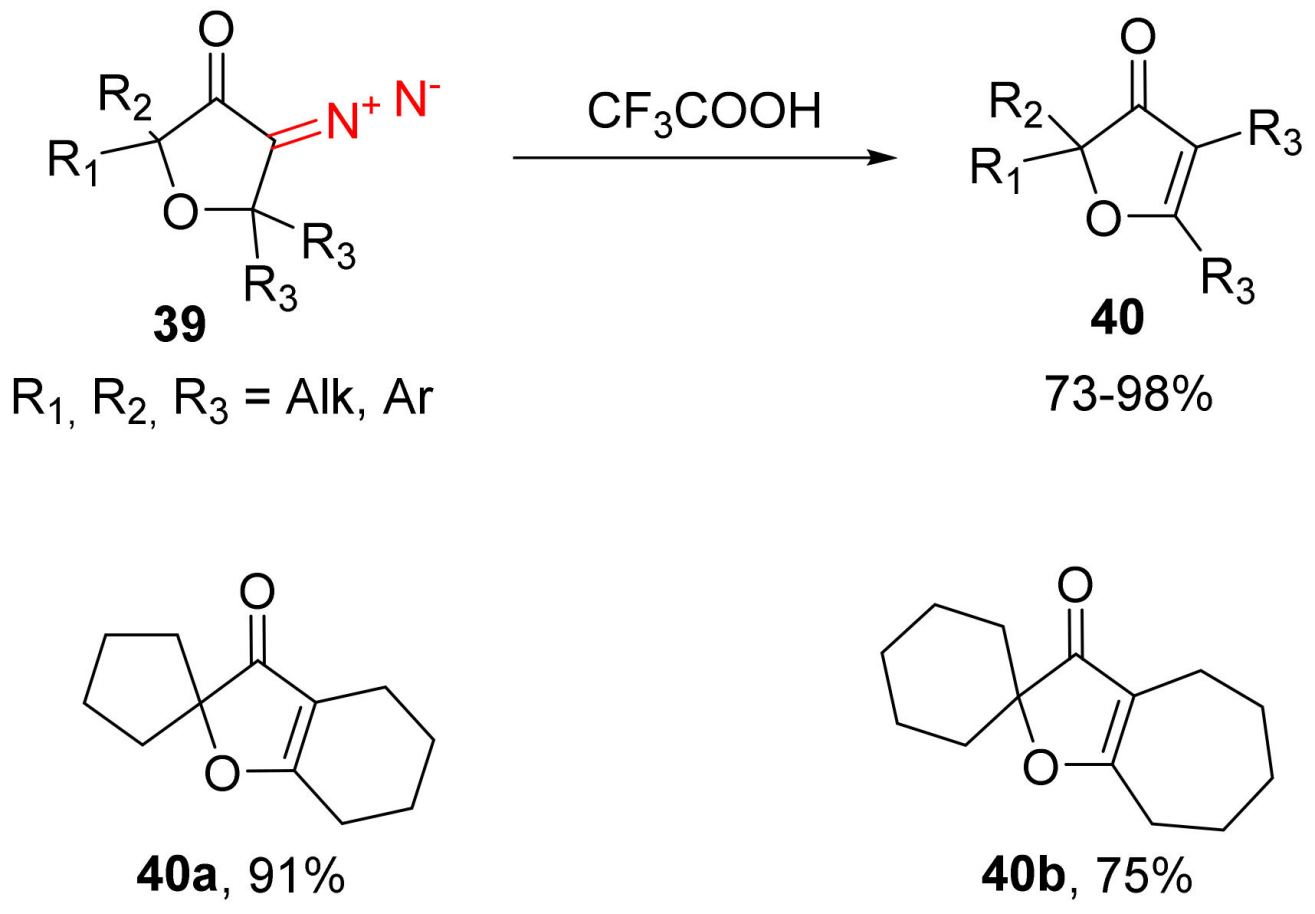
Decomposition of 3-diazodihydrofuran-3(2*H*)-ones **39** in trifluoroacetic acid.

With the decomposition of α-diazocarbonyl compounds, the resulting diazonium salts can be stabilized via the β-to-α migration of substituents. The stability of 3-diazodihydrofuran-3(2*H*)-ones **39** towards various protic acids was investigated. The compounds were stable in acetic and formic acids; however, in stronger trifluoroacetic acid, products of β-migration **40** were obtained in high yields. The decomposition of the spirocyclic structures gave annelated products **40a**–**b** (Scheme 25) [54].

**Scheme 26:**
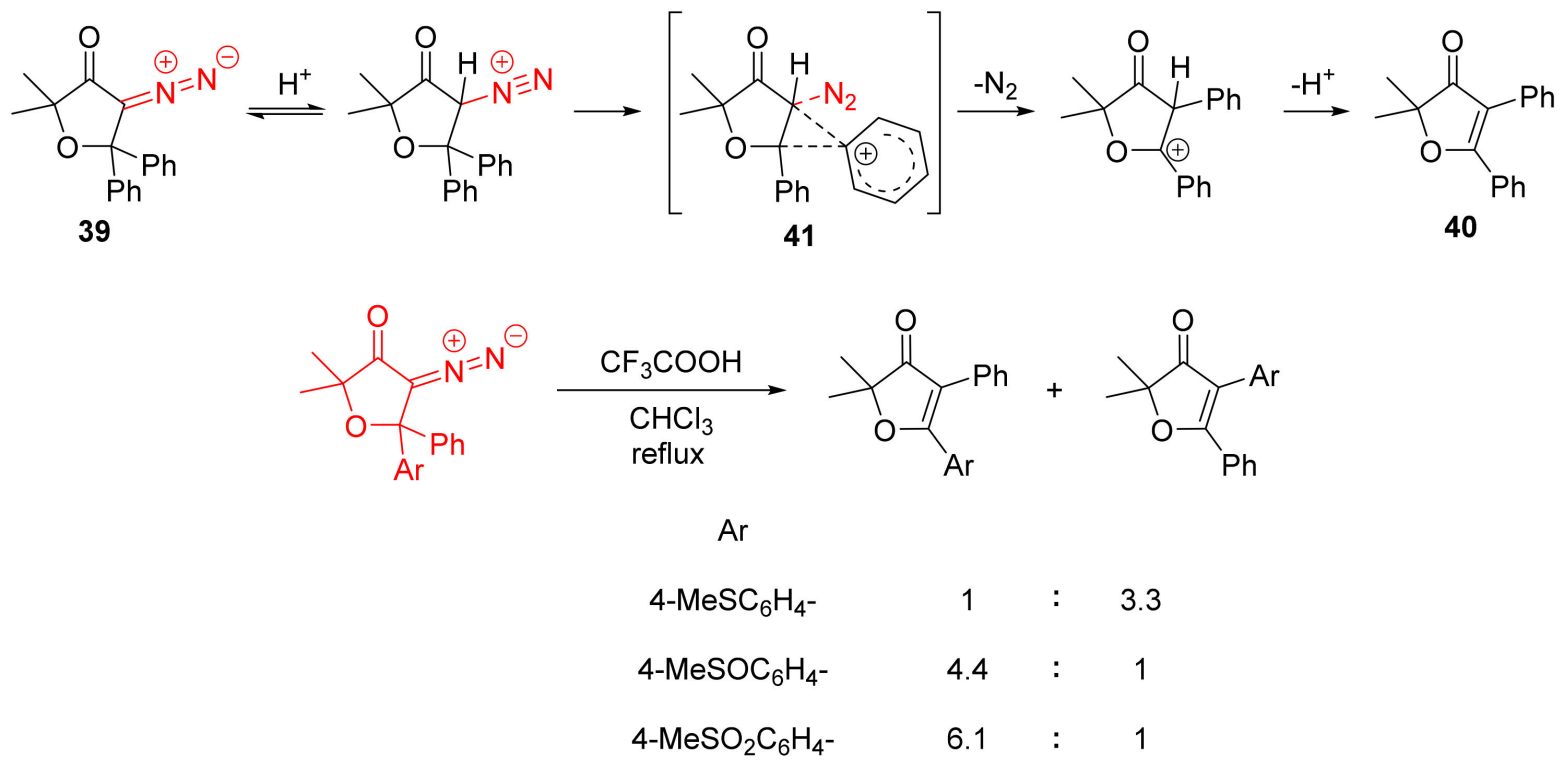
Mechanism of decomposition of **39** in trifluoroacetic acid.

Mechanistically, the protonation of the diazo group can lead to the formation of the transition state **41**. Upon migration, deprotonation gives the observed olefin **40**. This mechanism can justify the selectivity for products **39** containing two different aromatic groups. More electron-rich aryls tend to migrate preferentially (Scheme 26) [55].

**Scheme 27:**
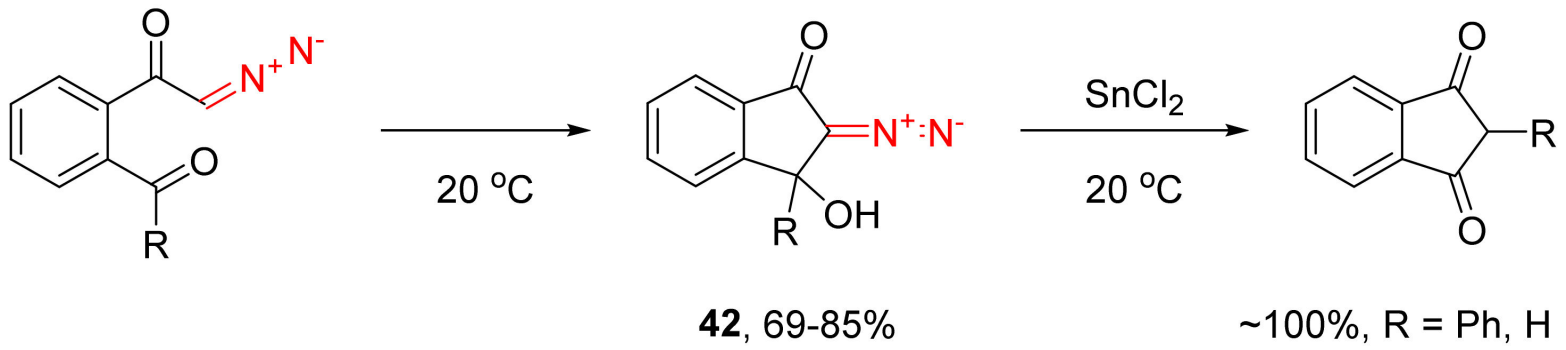
Tin(II) chloride catalyzed Tiffeneau–Demjanov rearrangement of **42**.

For β-hydroxy-substituted substrates, 1,2-migration leads to the enol formation (Tiffeneau–Demjanov rearrangement), whose main tautomer is the dicarbonyl compound. For instance, for 3-hydroxy-2-diazoindan-1-ones **42**, formed in situ as the result of intramolecular condensation, decomposition with the subsequent migration of the phenyl substituent or a hydrogen atom takes place on catalysis with tin(II) chloride (Scheme 27) [25]. The same transformation can be catalyzed by *p*-toluenesulfonic acid [55].

**Scheme 28:**
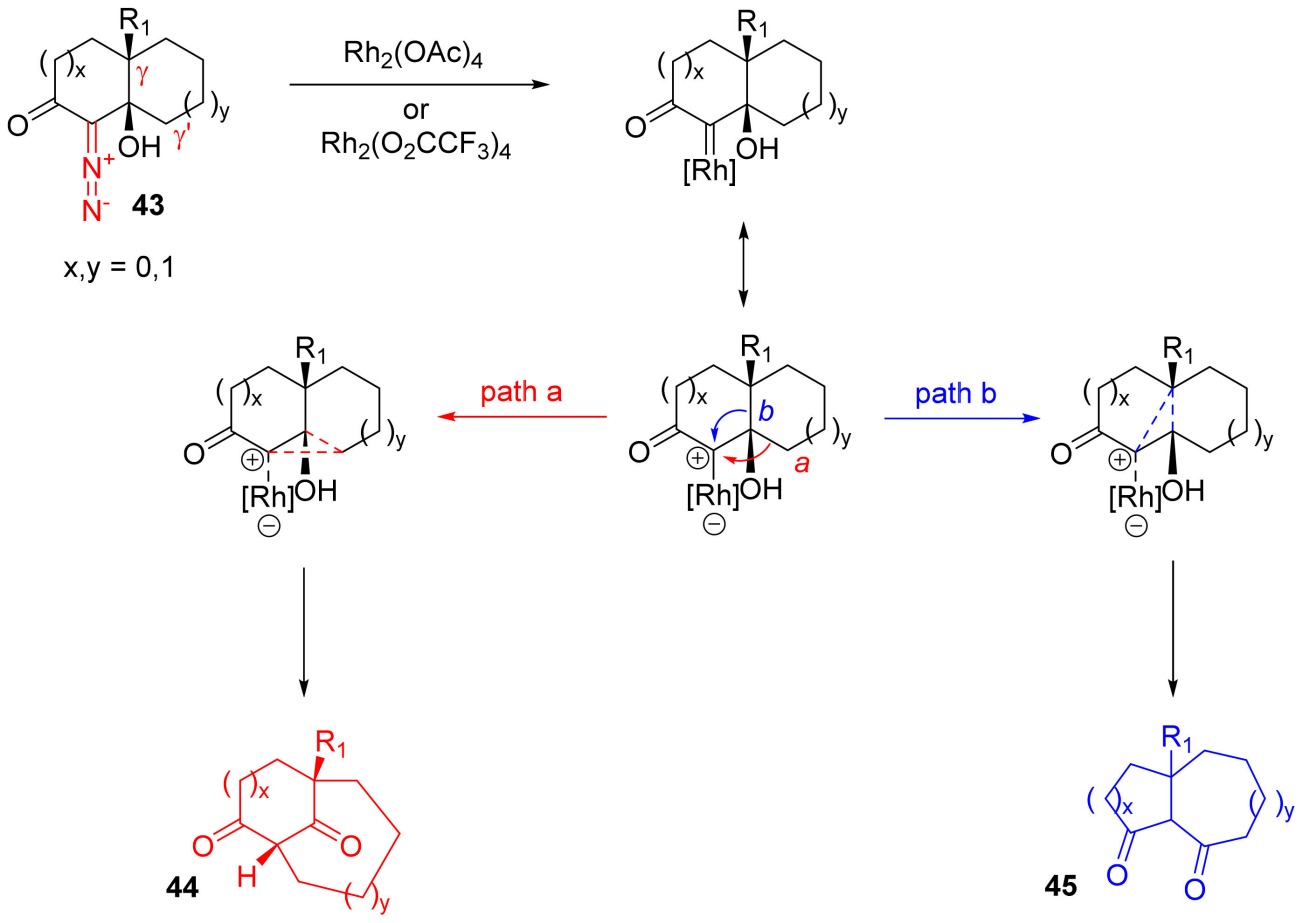
Rh(II)-catalyzed Tiffeneau–Demjanov rearrangement towards cage products.

The decomposition of α-diazo-β-hydroxyketones with a subsequent Tiffeneau–Demjanov rearrangement can be employed for building cage structures. Bicyclic α-diazo ketones bearing a hydroxy group in β-position relative to the diazo group (such as **43**) can be decomposed in the presence of tetracarboxylates of dirhodium. Since both carbon–carbon bonds leading to this hydroxy group can be fragmented, two products (**44** and **45**) can be formed in varying ratios. It is supposed that the alkyl migration takes place as a concerted process, which is supported by the preserved stereochemistry of the α-carbon atom (Scheme 28) [24].

**Scheme 29:**
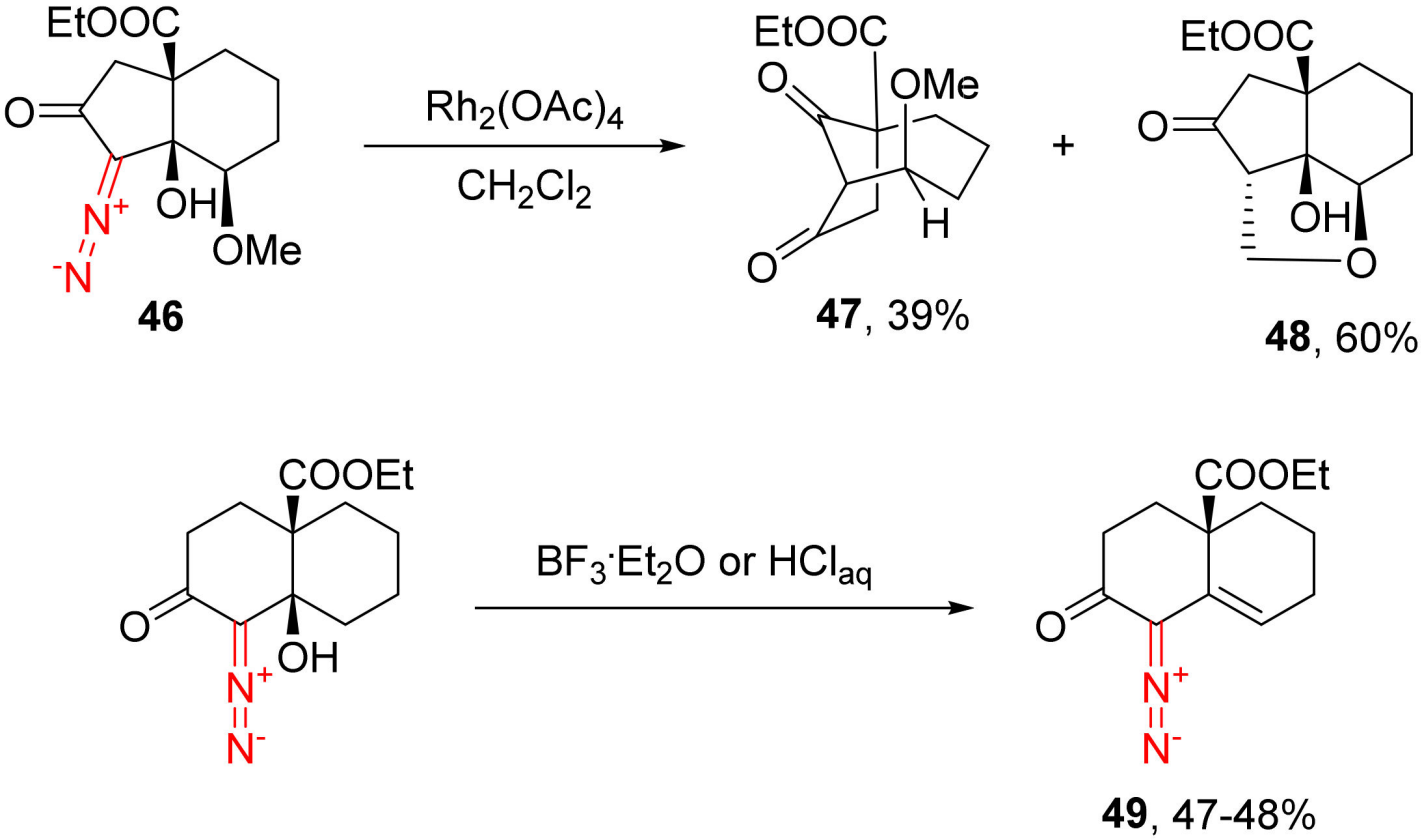
Rh(II)-catalyzed transformations of substrates **46**.

In the case of γ’-methoxy substitution (as in **46**), along with the rearrangement product (**47**), a substantial amount of intramolecular C-H insertion product **48** is formed, which is indicative of the reaction proceeding through rhodium carbenes. Additional evidence that the catalyst does not act as a Lewis acid is that the reaction is not workable with acidic catalysts (HCl or BF_3_·Et_2_O), in which case only products **49** of the dehydration of the starting alcohol are formed (Scheme 29) [24].

**Scheme 30:**
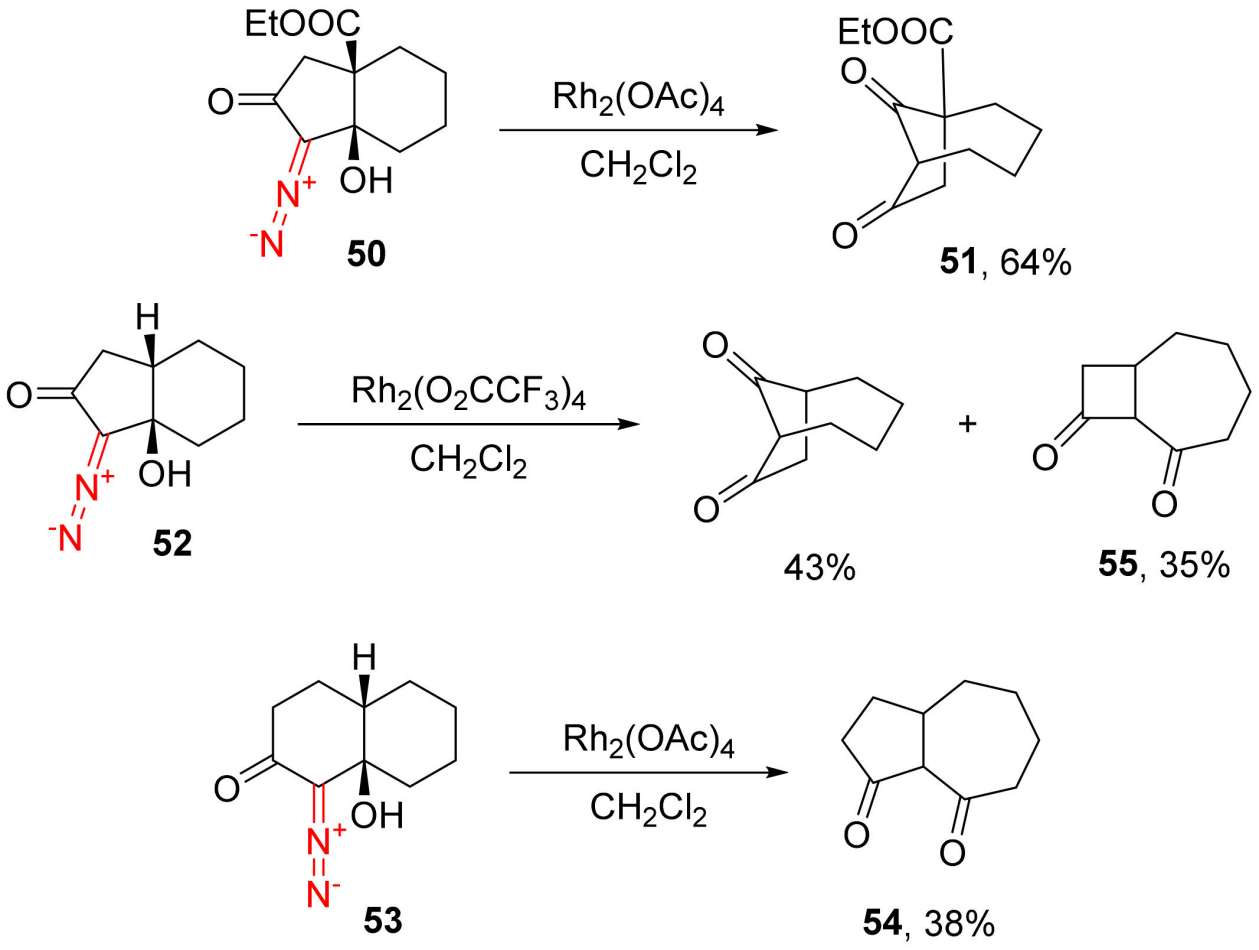
The influence of electronic and ring-size factors on the outcome of the Rh(II)-catalyzed rearrangement of α-diazocarbonyl compounds.

Since metal carbene is electrophilic in nature, the reactions primarily involve the rupture of the bond bearing higher electrophilic density on it. For instance, the introduction of an ethoxycarbonyl substituent in the γ-position of 1-diazo-7a-hydroxy octahydro-2*H*-inden-2-one **50** reduces electron *b*, and only the path *a* rearrangement product **51** is selectively formed. The selectivity is affected by the cycle size of the α-diazocarbonyl compound. Going from a five-membered cycle (**52**) to a six-membered cycle (**53**), the amount of the path *b* rearrangement product is increased. Due to stereochemical and energetic considerations, the formation of a cyclopentanone ring (**54**) is more preferred compared to a cyclobutene ring (**55**) (Scheme 30) [24].

The importance of the information summarized above is underscored by the frequent occurrence of a bicyclo[m.n.1]alkanone scaffold in various natural products [24]. The said transformation was employed in the construction of a welwistatin core (fragment of alkaloids of the bacteria, *Hapalosiphon welwitschii* and *Westiella intricata*) [56].

**Scheme 31:**
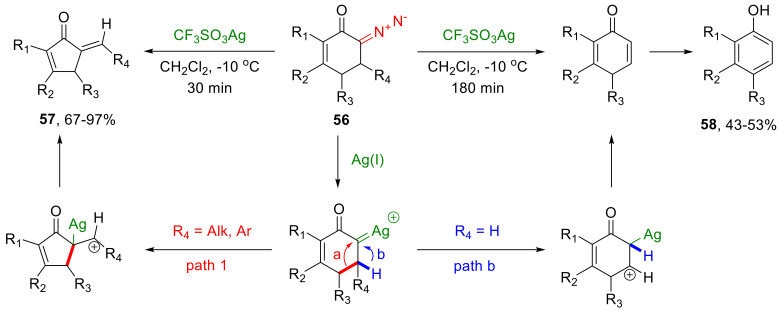
Transformations of diazo compounds **56** under silver triflate catalysis.

In an endocyclic mode, β-migration, not only of alkyl but also of allylic substituents, occurs. The decomposition of 6-diazocyclohex-2-enones **56** in the presence of silver triflate leads to the formation of a metal carbene undergoing an endocyclic migration, which leads, in turn, to the formation of 5-alkyliden-2-cyclopentenones **57** in high yields and with an exclusively *trans*-configured double bond. This transformation requires the presence of at least one substituent in position 5 of the starting diazo compound; otherwise, a β-hydride shift overrides the β-allyl migration, which leads to the formation of corresponding phenols **58** in moderate yields (Scheme 31) [57].

**Scheme 32:**
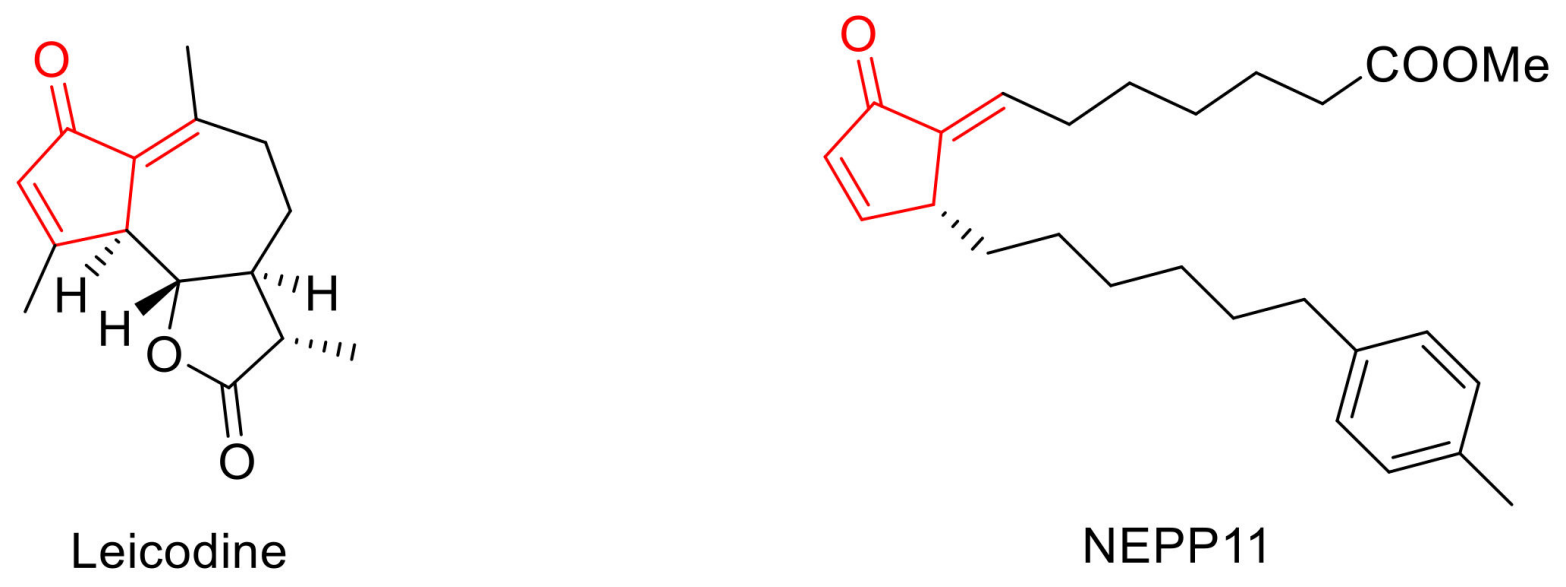
Examples of biologically active compounds comprising a 5-alkylidene-2-cyclopentenone scaffold.

The decomposition of 6-diazocyclohex-2-enones with a subsequent endocyclic β-migration have been found to tolerate the presence of bulky substituents in the substrate. As to the electronic properties, slightly better yields have been observed for substrates bearing an electron-donating group in position 5 of the starting diazo compound, which confirms the reaction proceeding via cationic intermediates. This transformation has an undisputed practical significance, as the resulting 5-alkylidene-2-cyclopentenone scaffold is present in many biologically active compounds (Scheme 32) [58,59].

**Scheme 33:**
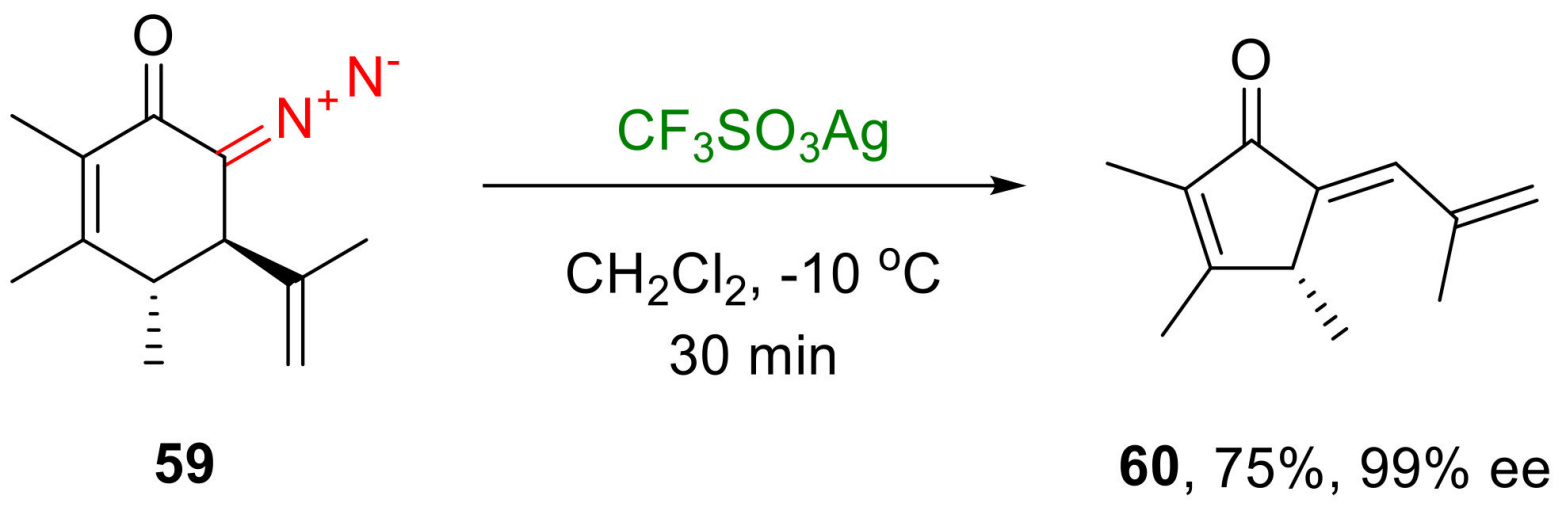
Example of chirality transfer from diazo substrates **59**.

More practical aspects of the described method become apparent in the case of using 6-diazocyclohex-2-enones with a chiral center in position 5 (such as **59**), which can easily be synthesized from suitable natural products. With the decomposition of such substrates and a subsequent β-migration, this stereocenter is usually not epimerized, which allows the obtaining of products **60** with high enantiopurity (Scheme 33) [57].

**Scheme 34:**
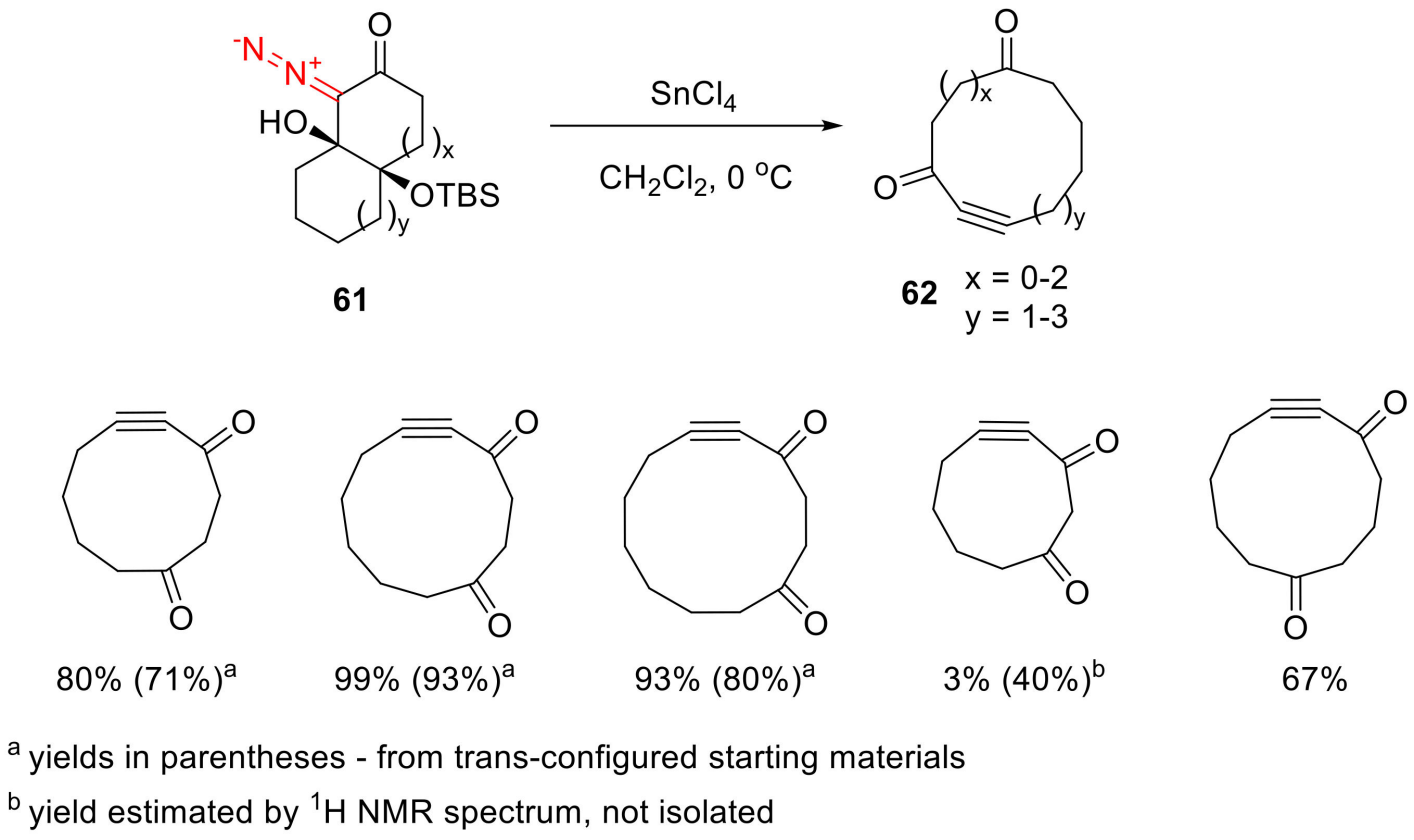
Sn(IV) chloride promoted fragmentation of bicyclic β-hydroxy-α-diazo-γ-silyloxy ketones **61**.

Of special interest is the fragmentation of bicyclic β-hydroxy-α-diazo-γ-silyloxy ketones **61**. Their decomposition, catalyzed by Sn(IV) chloride under mild conditions, delivers macrocyclic alkynones **62**. The transformation turns out to be sensitive to the ring size bearing the diazo function. Good results have been obtained with α-diazo cyclohexanone templates. Reducing the ring size to five-membered leads to worse results, according to spectroscopic data; however, isolating it is not feasible due to instability. With the fragmentation of the starting material with an α-diazo cycloheptanone moiety, isolation of the product was possible, but with a significantly reduced yield compared to the six-membered counterpart. Unfortunately, synthesis of an α-diazo cyclooctanone fragment-containing substrate is not feasible. Notably, the reaction is also successfully realized for the trans-isomers of compounds bearing an α-diazo cyclohexanone fragment; however, the yields are generally lower than for the cis counterparts (Scheme 34) [26].

**Scheme 35:**
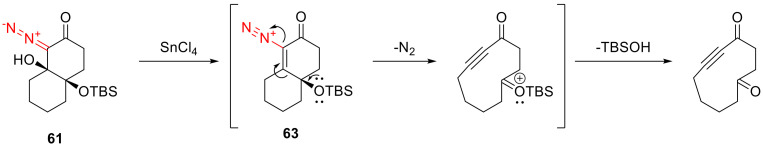
Mechanism of Sn(IV) chloride promoted fragmentation of **61**.

Mechanistically, as it was established for simpler substrates [60], the reaction proceeds via the Lewis acid promoted elimination of the hydroxy group, with the formation of vinyldiazonium intermediate **63**. Due to the high electron density on the δ-oxygen atom, intermediate **63** undergoes the Grob fragmentation, with the elimination of the nitrogen molecule and formation of a triple bond. Desilylation at the last step gives the final product (Scheme 35).

The presence of the silyloxy group is crucial for the reaction to reach completion. Should the γ-position be substituted with a hydroxy group, intermediate 63 would simply turn into a vinyl cation with a subsequent ring expansion due to the 1,2-alkyl migration [61]. In principle, the silyloxy group could be replaced with a donor alkyl substituent; however, this leads to lower product yields [60]. That is why a strongly donating silyloxy group appears crucial for the fragmentative formation of complex macrocyclic systems [26].

### 2.4. Decomposition of Cyclic α-Diazo Ketones in the Presence of Substrates Containing n-Nucleophiles

**Scheme 36:**
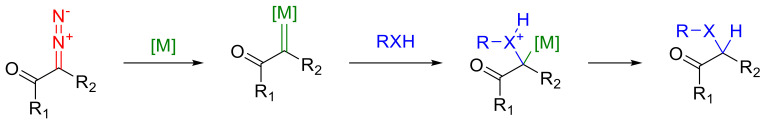
General metal carbene X-H insertion reactions.

The reactions of the decomposition of cyclic α-diazo ketones in the presence of substrates containing n-nucleophiles are well-represented in the literature. The simplest case of this sort of transformation are the reactions of the formal insertion of carbenes into a hydrogen–heteroatom bond (X-H). These reactions presumably proceed through an ylide formation, followed by a proton migration (Scheme 36) [2].

**Scheme 37:**
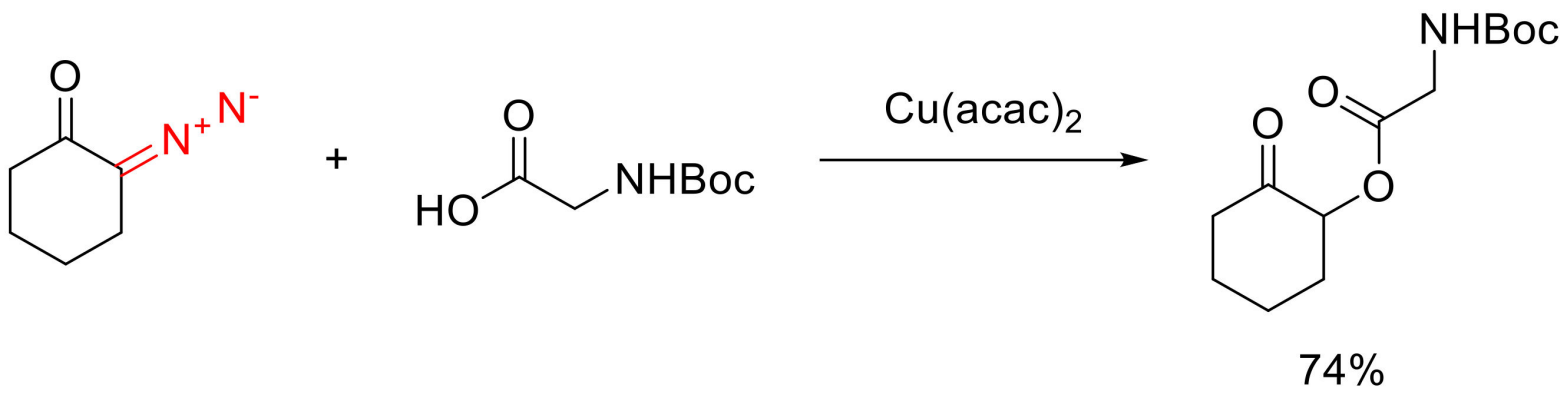
Cu(acac)_2_ catalyzed selective O-H insertion into Boc-protected glycine.

The O-H insertion reactions are known for carboxylic acids as well as alcohols. Cyclic α-diazo ketones react with aliphatic carboxylic acids in the presence of the Cu(acac)_2_ catalyst. The substrates are apparently stable in the presence of carboxylic acids, which is not the case for all diazo compounds. The insertion into the O-H bond of *N*-protected amino acids is selective over the insertion into the amide N-H bond (Scheme 37) [62].

**Scheme 38:**
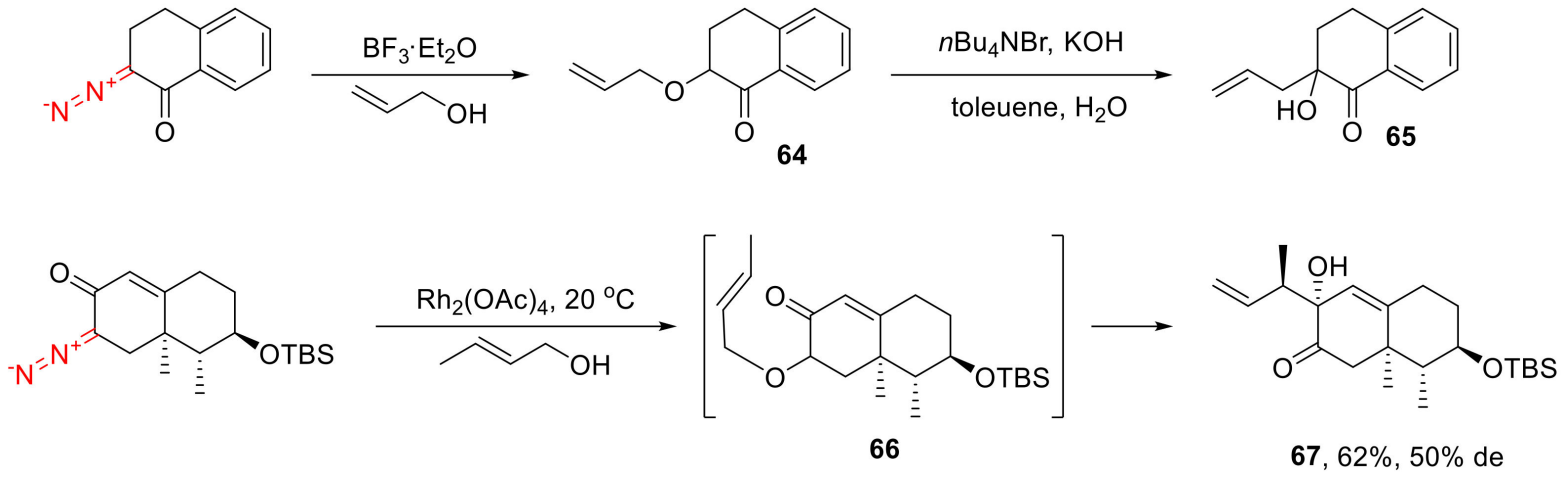
Allylic O-H insertions accompanied by sigmatropic rearrangements.

The insertion into the O-H bond of alcohols is poorly studied. However, selected literature examples demonstrate that the reaction can be catalyzed by Lewis acids [63,64] as well as transition metal complexes [65]. The insertion into the O-H bond of allylic alcohols is of much interest, as it can be accompanied by a subsequent sigmatropic rearrangement. Under basic conditions, α-allyloxyketones, such as **64**, selectively undergo a [2,3]-sigmatropic rearrangement to give α-allyl-α-hydroxy ketones, such as **65** [63,66]. Alternatively, a [3,3]-sigmatropic rearrangement (**66**→**67**) proceeds spontaneously [15] or with thermolysis (Scheme 38) [23,66].

**Scheme 39:**
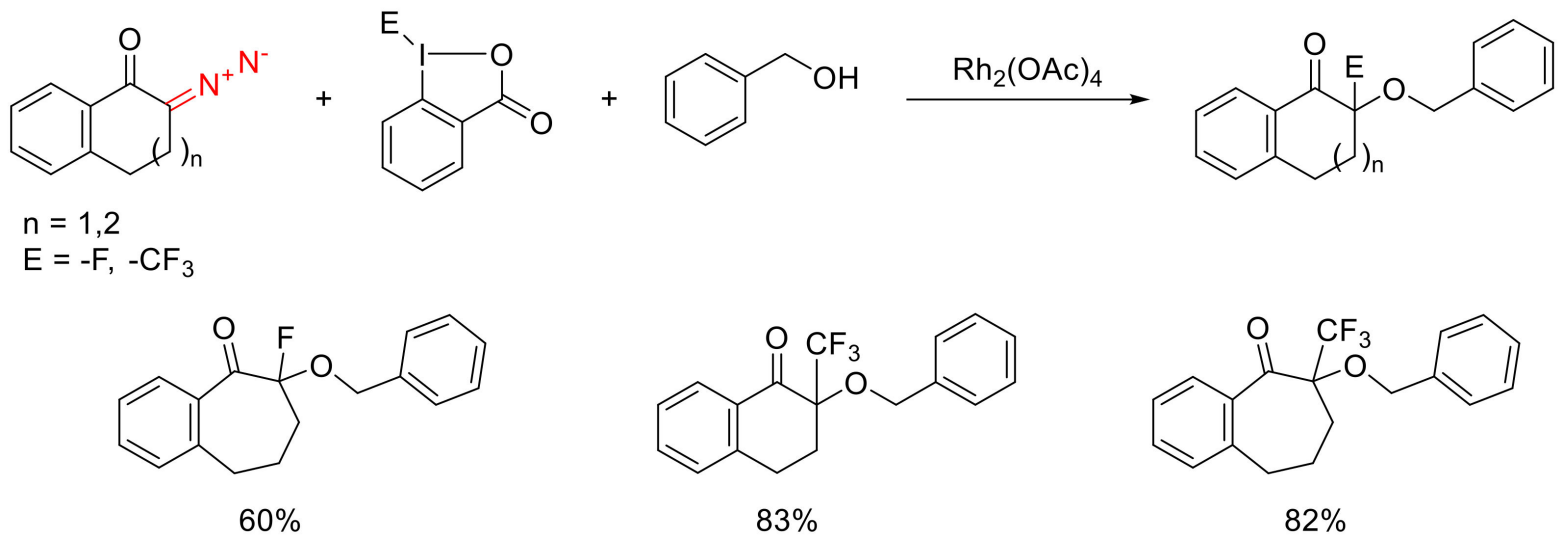
Examples of oxonium ylide interception by an electrophilic fluorine or trifluoromethyl group.

The onium ylide formed in the course of the catalytic decomposition of cyclic diazo ketones can be stabilized, not only due to proton migration, but also as the result of an external electrophile addition. A fluorine or trifluoromethyl group bound to hypervalent iodine can act as such an intercepting electrophile (Scheme 39) [67].

**Scheme 40:**
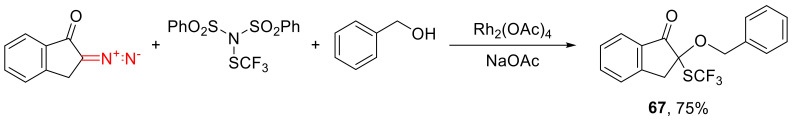
Example of oxonium ylide interception by an electrophilic SCF_3_.

Moreover, α-diazoindanone, among other diazocarbonyl compounds, reacts with benzyl alcohol in the presence of an electrophilic SCF_3_ source to give the respective α,α-bifunctionalized indane **67** (Scheme 40) [68].

**Scheme 41:**
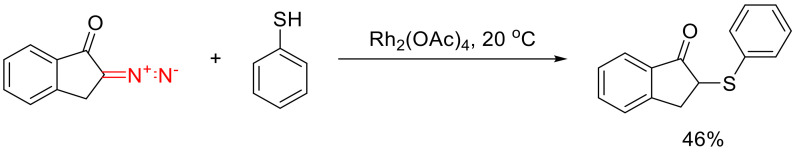
An example of S-H insertion of Rh(II) carbene derived from a cyclic α-diazo ketone.

Formal S-H insertion reactions are represented in the literature on cyclic α-diazo ketones, by a few examples involving thiophenol with a catalysis by dirhodium tetraacetate (Scheme 41) [69,70].

**Scheme 42:**
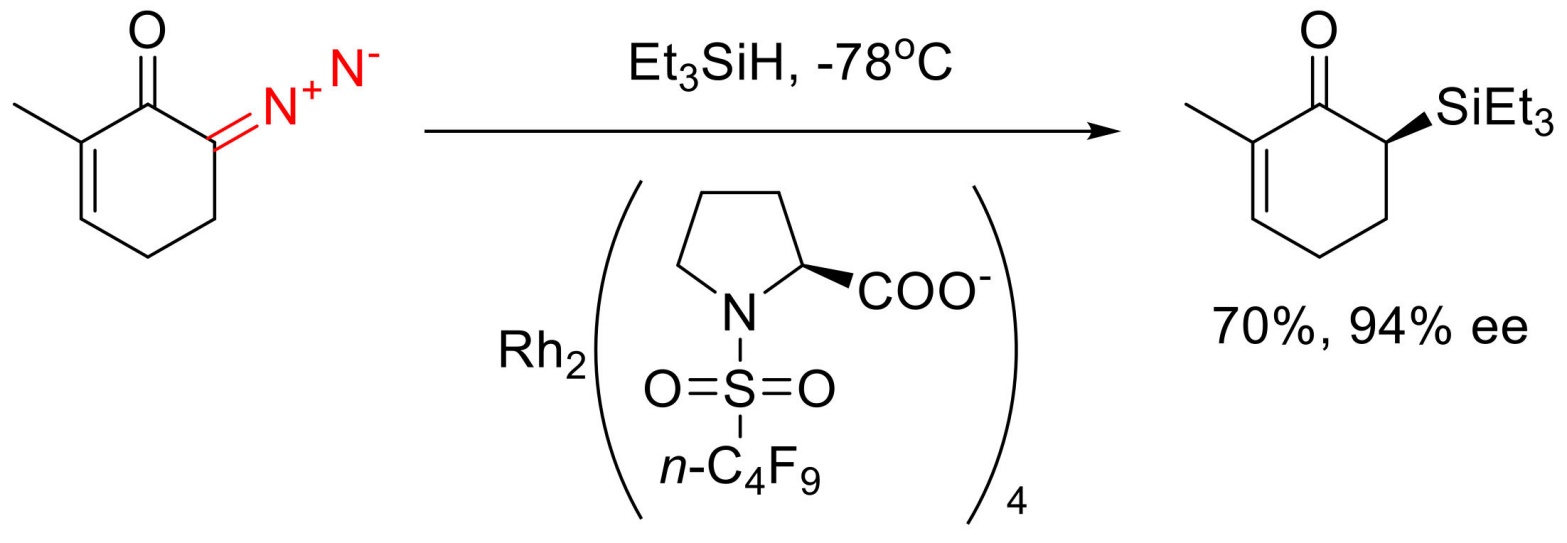
An example of enantioselective Si-H Rh(II) carbene insertion.

Noteworthy are the formal Si-H insertion reactions described for various silanes, including enantioselective versions (Scheme 42) [14,71].

**Scheme 43:**
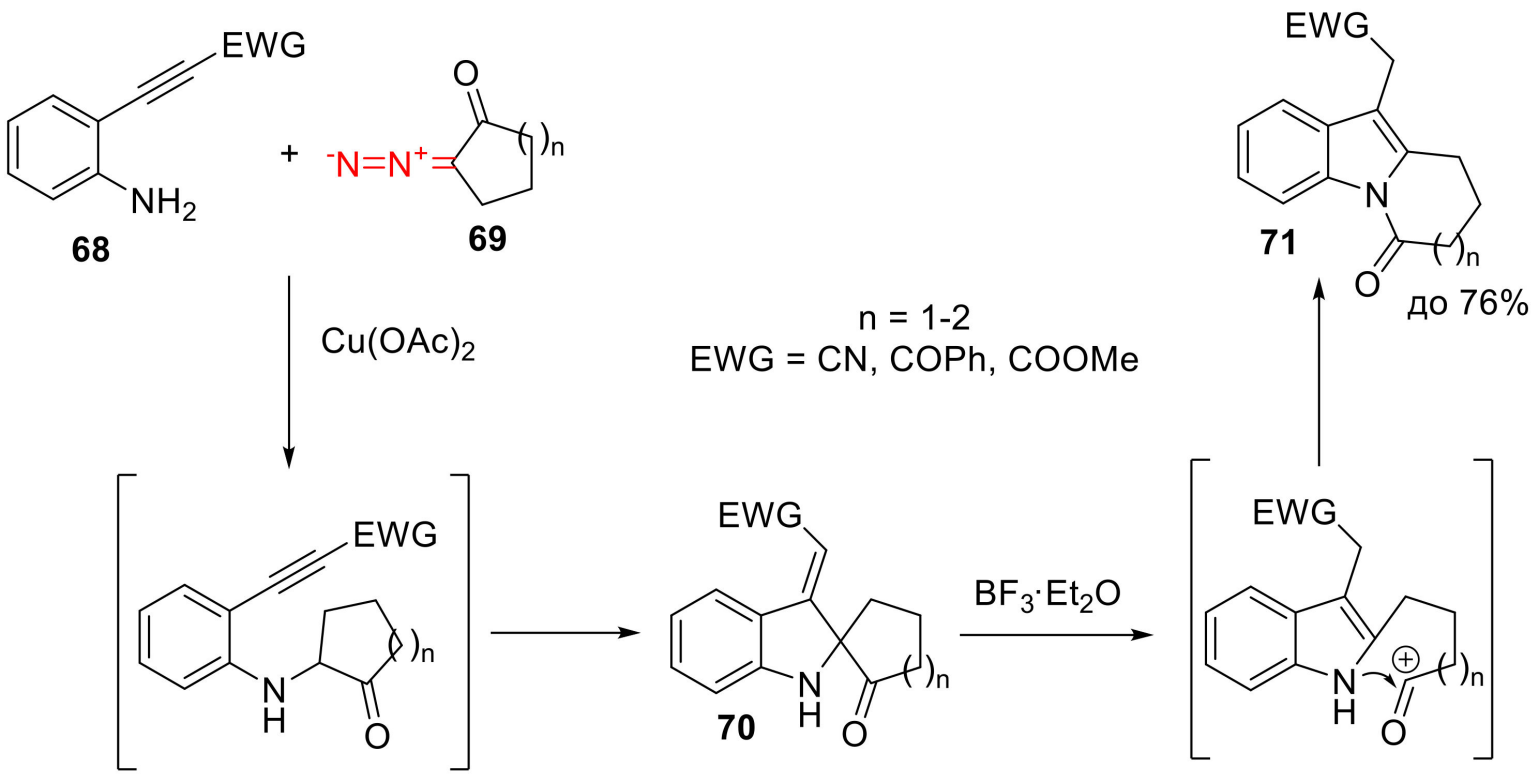
Cascade transformation leading to indole derivative **71**.

The N-H insertions of cyclic α-diazo ketones is well-known. However, as such, without subsequent post-transformations, only one example is known (the reaction of β-diazo-α-tetralone and *tert*-butylamine) [72]. Much more intriguing are those reactions that involve amines containing an electrophilic moiety in its structure. For example, the product of the Cu(II)-catalyzed reaction of *o*-alkynyl anilines **68** with cyclic α-diazo ketones **69** immediately undergoes a Michael reaction, giving spirocyclic product **70**, which is then fragmented and recyclized with the addition of BF_3_∙OEt_2_. This results in indole derivatives **71**, which are valuable precursors en route to natural products (Scheme 43) [73].

**Scheme 44:**
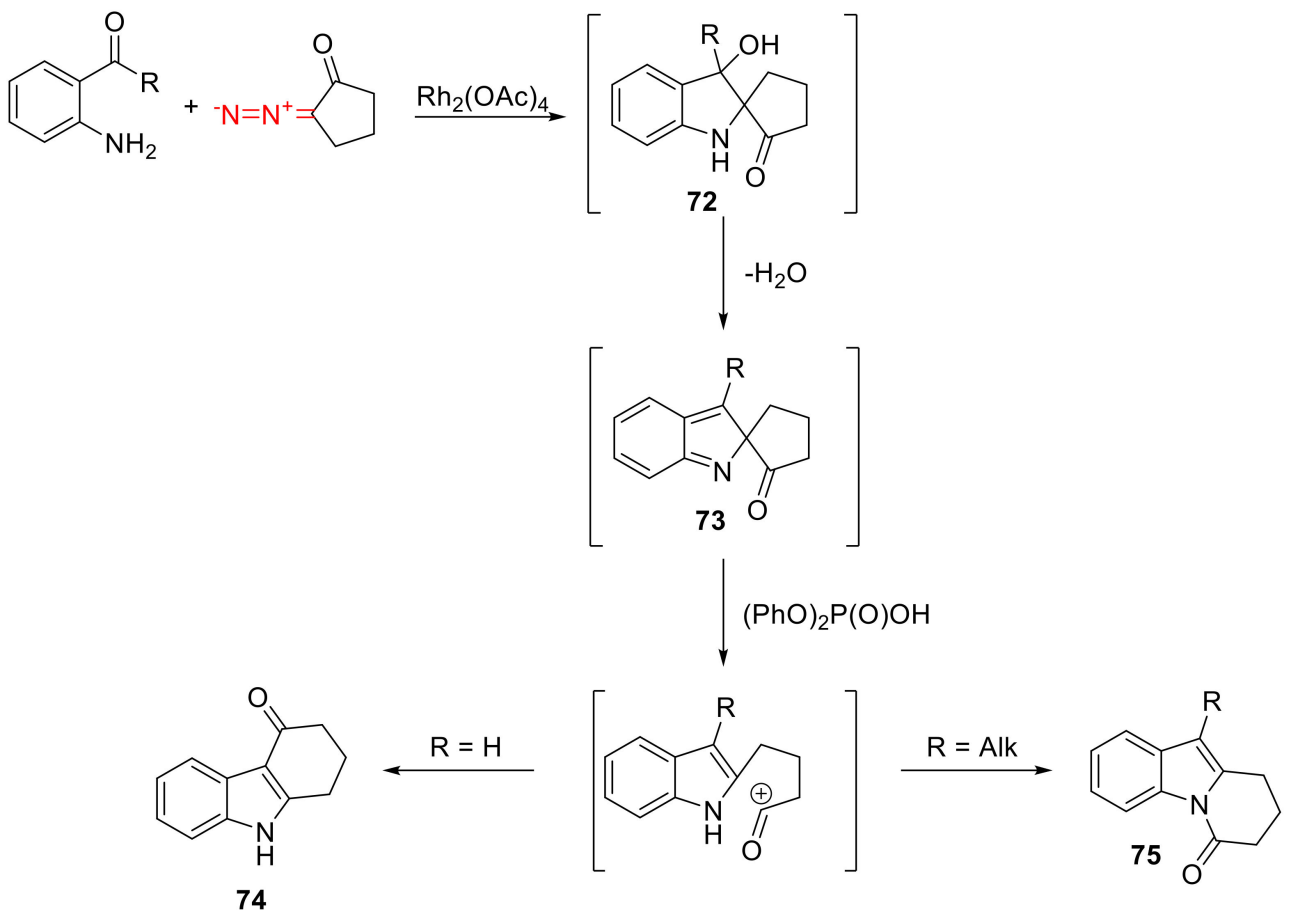
Rh(II)-catalyzed reactions of α-diazo cyclopentanone.

In addition to *o*-alkynyl anilines, anthranilic aldehydes and *o*-amino acetophenones can participate in similar reactions. The reactions also proceed via spirocyclic intermediate **72**, which is dehydrated to give 2*H*-indole **73**. Following fragmentation, the reaction can take either direction depending on the presence of a substituent in position 3, that is, on the structure of the starting aminocarbonyl partner. For anthranilic aldehydes (containing no such substituent), the intramolecular acylation of the indole nucleus is the predominant pathway, resulting in the formation of carbazoles **74**. For *o*-amino acetophenones, *N*-acylation prevails, resulting in the formation of indolones **75** (Scheme 44) [74].

**Scheme 45:**
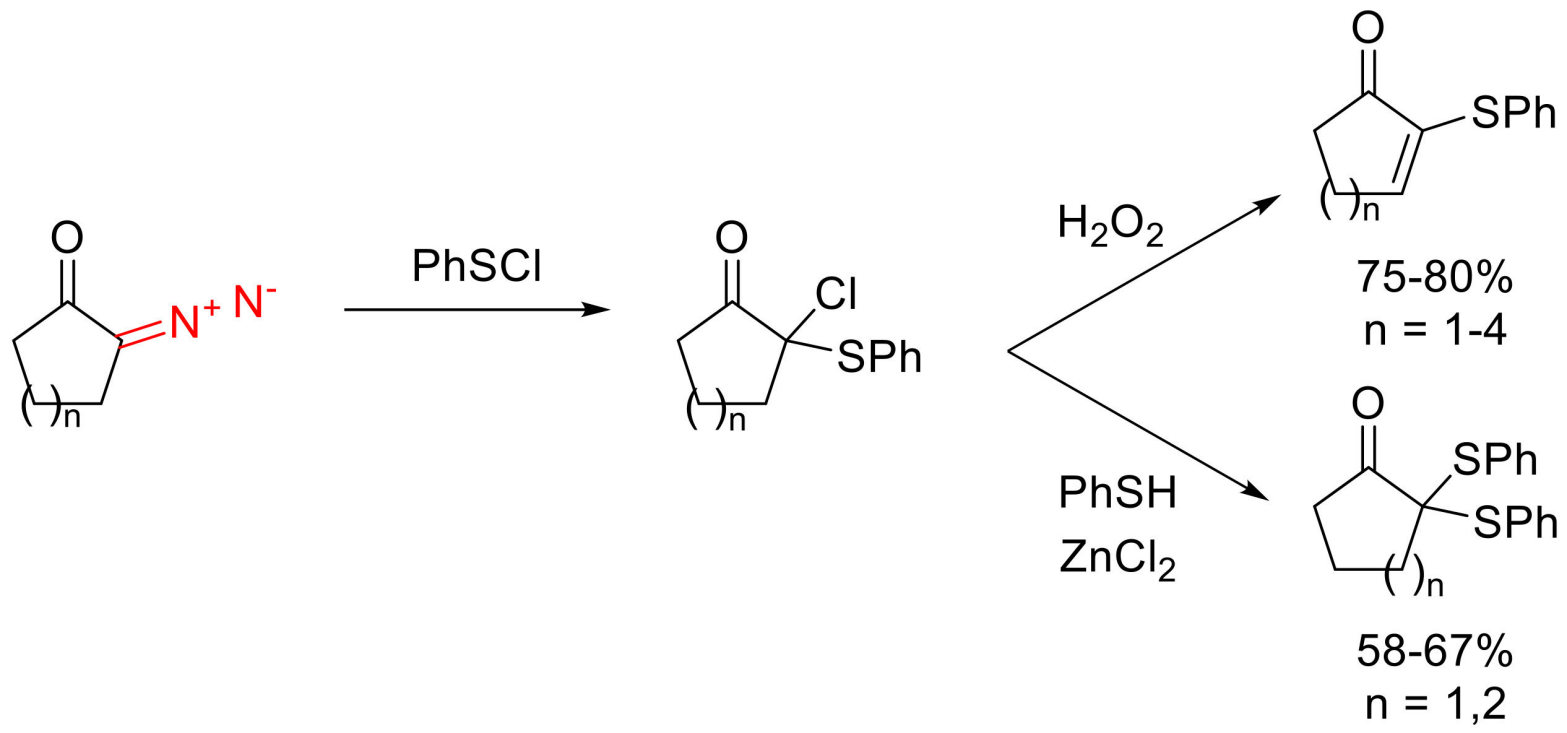
Carbene insertion into S-Cl bond and subsequent transformations.

Cyclic α-diazo ketones also can participate in a formal chalcogen–halogen bond insertion. The resulting products themselves are of little interest. However, their postmodifications can give rise to difficult-to-access compounds. For instance, the reaction with phenyl sulfenyl chloride with subsequent hydrogen peroxide oxidation gives high yields of α-phenylthio-α,β-unsaturated cyclic carbonyl compounds [75]. Instead of oxidation, the chlorine atom in the initial adducts can be replaced with thiophenol in the presence of Zn(II) chloride to give α,α-bis(arylthio)ketones (Scheme 45) [76].

**Scheme 46:**
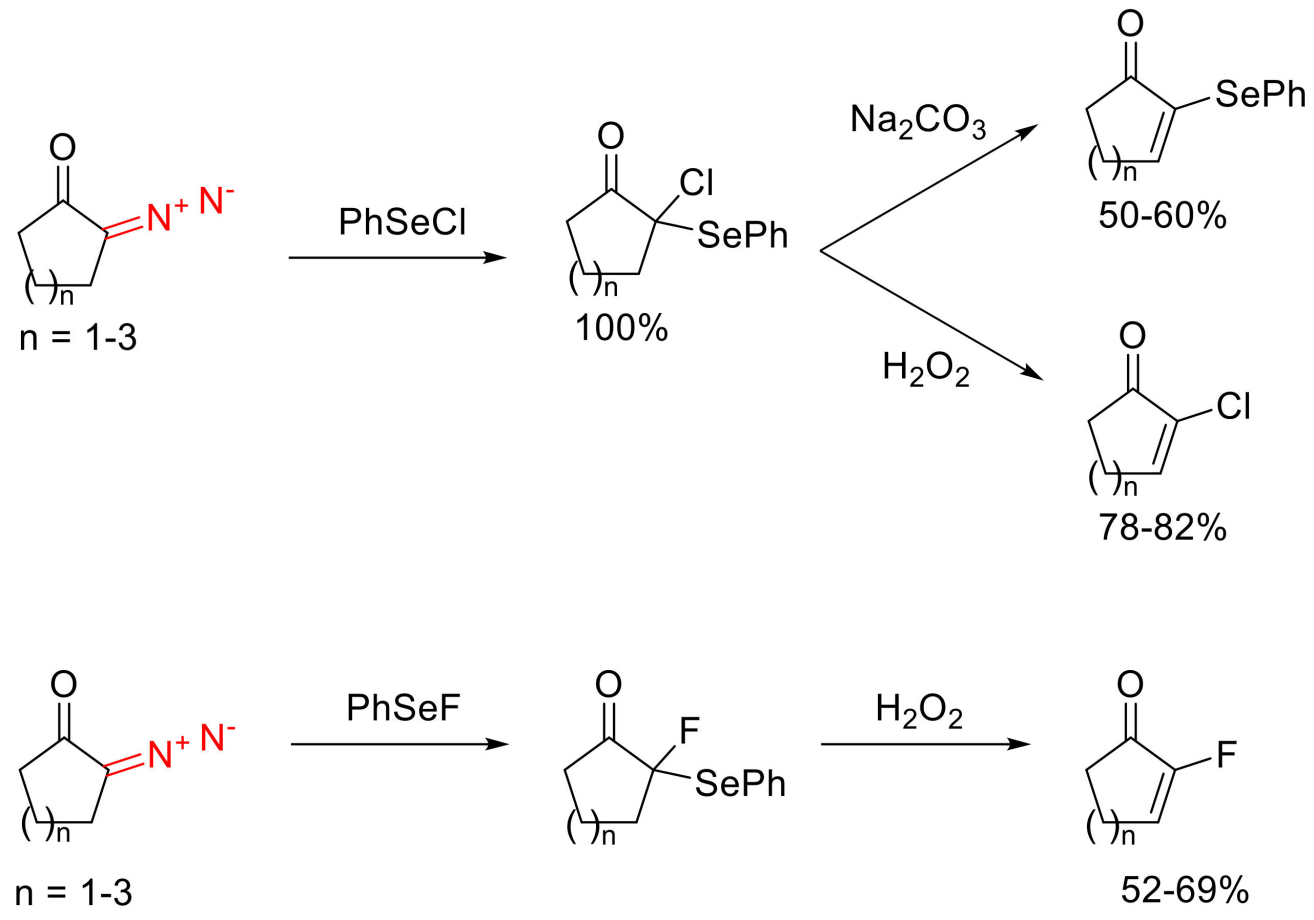
Examples of carbene insertion into Se-Hal bonds.

α-Halo-α-arylselenyl ketones, resulting from the reaction of cyclic α-diazo ketones with phenyl selenyl halides, can also be oxidized. However, in contrast to their sulfur-containing analogs, this will lead to α-halo-α,β-unsaturated cyclic carbonyl compounds. Unsaturated compounds can be obtained with the PhSe group intact, with treatment with sodium carbonate on the products of formal insertion into PhSeCl (Scheme 46) [77,78].

### 2.5. Reactions of Cyclic α-Diazo Ketones Not Involving Nitrogen Elimination

**Scheme 47:**
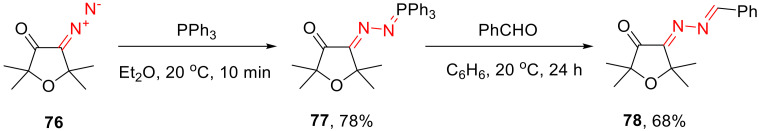
The Staudinger reaction of 4-diazotetrahydrofuran-3-ones **76** and subsequent transformation.

The simplest case of this type of reaction of cyclic α-diazo ketones is the Staudinger reaction reported for 4-diazotetrahydrofuran-3-ones **76**. The reaction is a fast one and gives phosphazines **77** in good yields. The treatment of the latter with aldehydes delivers bis-hydrazones **78** (Scheme 47) [79].

**Scheme 48:**
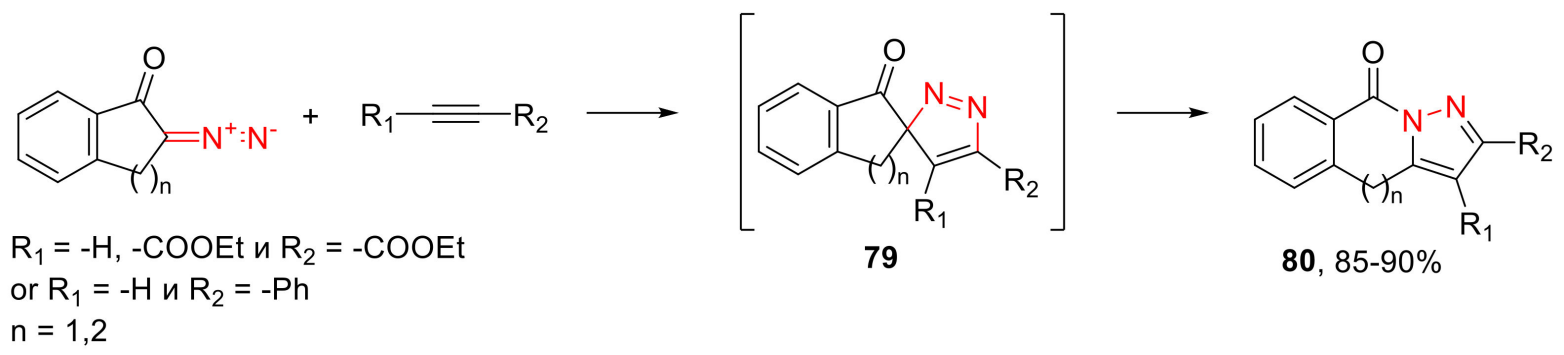
Dipolar cycloaddition of cyclic α-diazo ketones with acetylenes.

Cyclic α-diazo ketones can act as 1,3-dipoles in cycloaddition reactions with various dipolarophiles, such as electron-deficient acetylenes. The initial spirocyclic adduct **79**, formed as the result of a 1,3-dipolar cycloaddition, undergoes subsequent recyclizations as the result of an acyl migration delivering observable pyrazoles **80** (Scheme 48) [80,81,82]. In cases when electron-deficient olefin acts as the dipolarophile, recyclization does not take place [83].

**Scheme 49:**
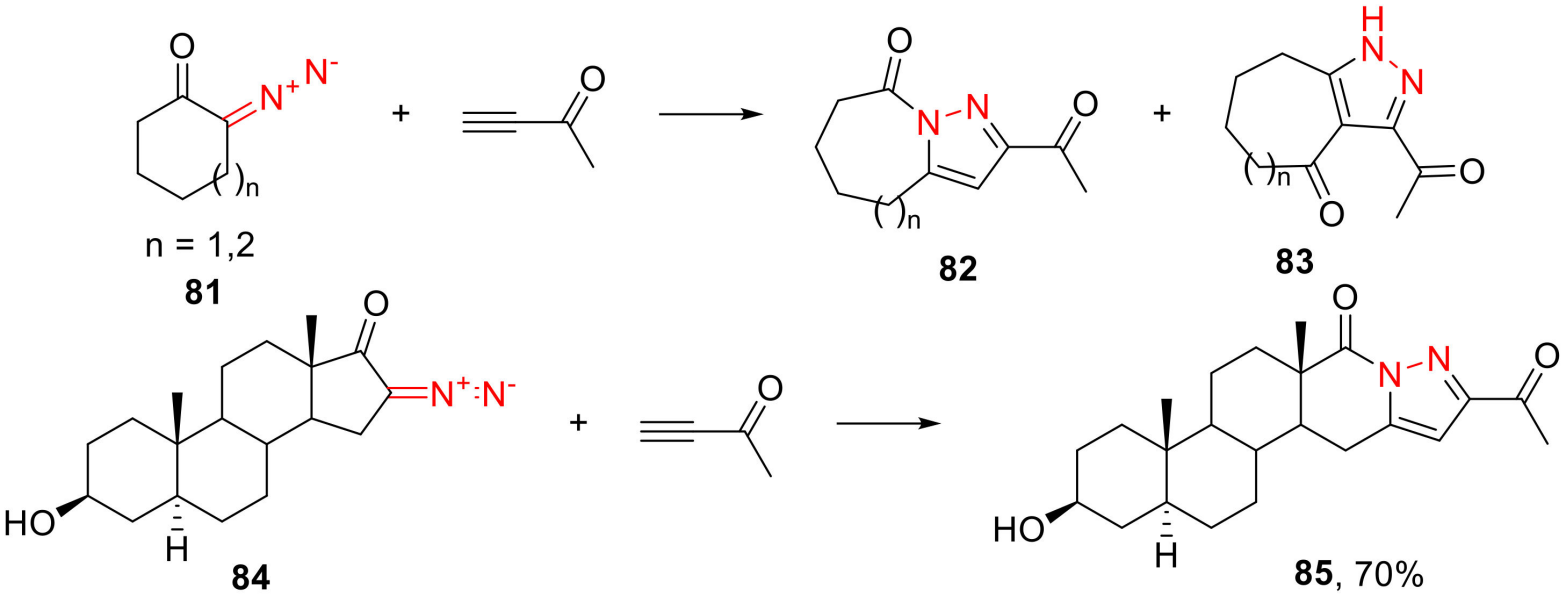
The influence of the ring size of the α-diazo ketone on the outcome of dipolar cycloaddition.

For more conformationally labile α-diazo ketones **81** with terminal alkynes, the intermittent acyl migration can occur toward either the pyrazole nitrogen atom or the symmetrically positioned carbon atom, in which case a mixture of two regioisomers (**82** and **83**, respectively) is formed. More conformationally rigid α-diazo ketones, such as **84**, have only one option for the acyl migration, and only one product (**85**) is observed (Scheme 49) [84].

**Scheme 50:**
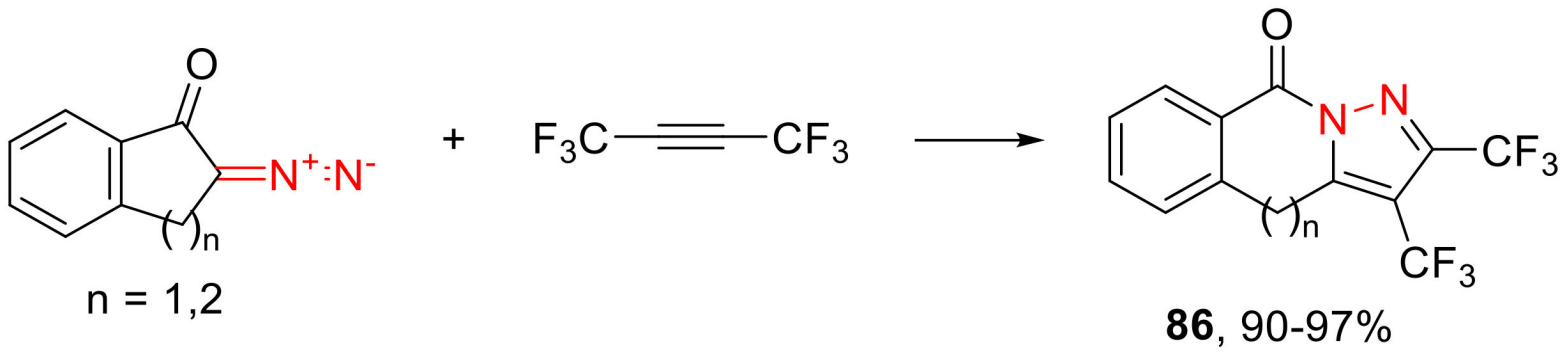
Synthesis of bis(trifluoromethyl)pyrazoles **86**.

Using bis(trifluoromethyl)acetylene as the dipolarophile in the reactions with α-diazo ketones, high yields of medicinally important bis(trifluoromethyl)pyrazoles **86** can be obtained (Scheme 50) [85].

**Scheme 51:**
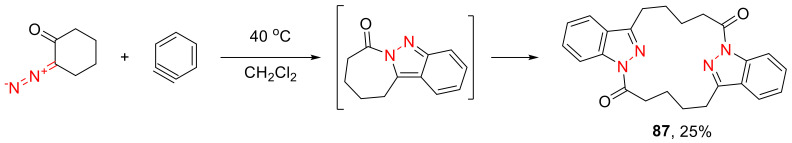
Formation of a dimeric product in dipolar cycloaddition with benzyne.

Attempted in 1973, the use of benzyne as the dipolarophile in reaction with α-diazo cyclohexanone only resulted in the formation of dimeric product **87** as the result of intermolecular acyl migration (Scheme 51) [86].

**Scheme 52:**
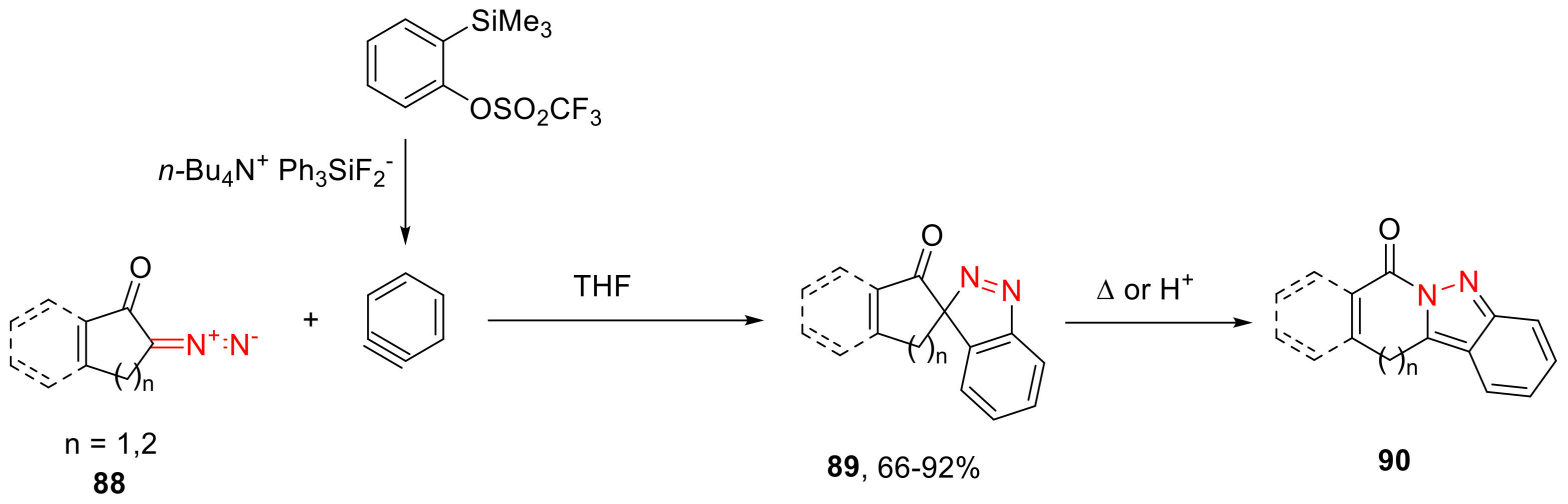
Dipolar cycloaddition with benzyne of more rigid α-diazo ketones.

Conformationally more rigid (compared to α-diazo cyclohexanone) α-diazo ketones **88**, in reactions with in situ generated benzyne, give spirocyclic intermediates **89**, which turn out to be stable at room temperature and are isolated in high yields. They are easily converted into the respective condensed 2*H*-indazoles **90** in an acidic medium or with simple heating (Scheme 52) [87].

### 2.6. Other Reactions of Cyclic α-Diazo Ketones

**Scheme 53:**
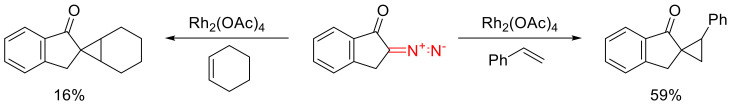
2-Diazo-1-indanone in Rh(II)-catalyzed cyclopropanation reactions.

2-Diazo-1-indanone was investigated in Rh(II)-catalyzed cyclopropanation reactions. With cyclohexene, the yields were markedly lower than those obtained with styrene (Scheme 53) [69].

**Scheme 54:**
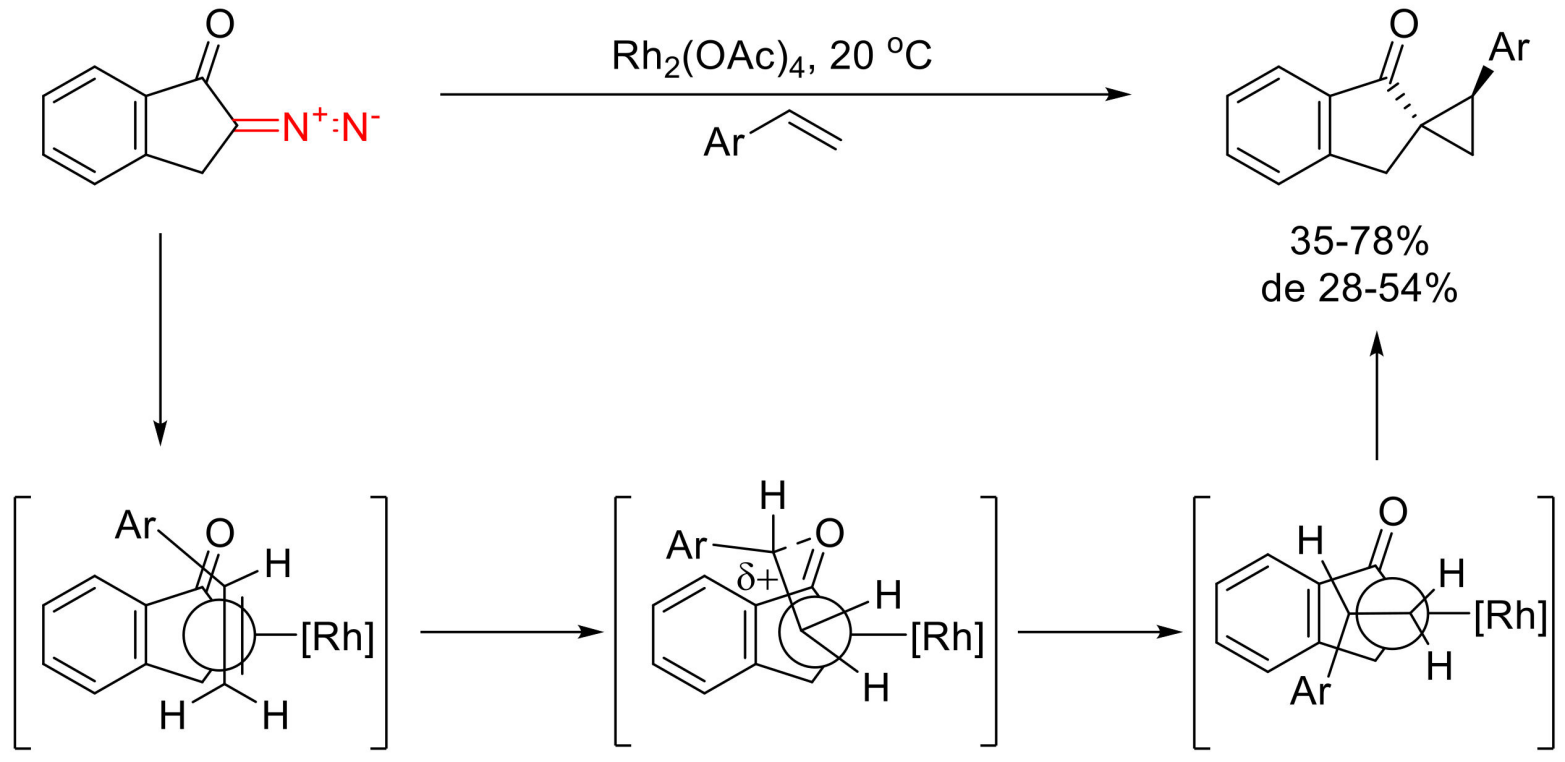
Diastereoselectivity aspects of the 2-diazo-1-indanone behavior in Rh(II)-catalyzed cyclopropanation reactions.

Subsequently, the stereochemical aspects of 2-diazo-1-indanone behavior in Rh(II)-catalyzed cyclopropanation reactions with various styrenes were investigated in detail. It was established that, in most cases, the major diastereomer formed has the aryl group in trans-orientation to the carbonyl group, while the reaction is practically insensitive to the steric bulk of that substituent. It was proposed that the underlying reason for such diastereoselectivity is in the stabilization by the carbonyl group of the partial positive charge in the benzylic position (Scheme 54) [88].

**Scheme 55:**
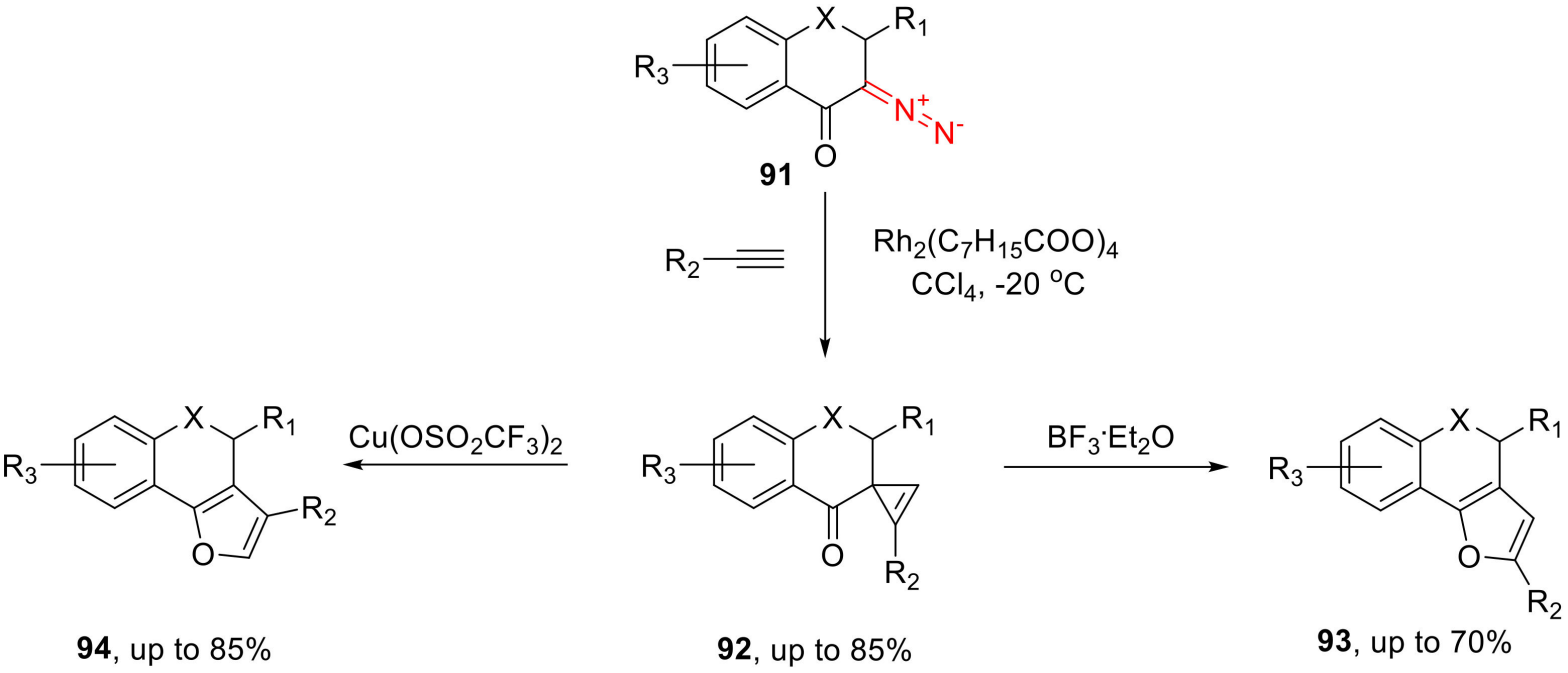
3-Diazochroman-4-ones **91** in cyclopropenation reactions and further transformations.

3-Diazochroman-4-ones **91** (along with 2-diazoindan-1-one and β-diazo-α-tetralone) undergo a cyclopropenation reaction with various terminal acetylenes, catalyzed by dirhodium tetraoctanoate delivering spirocyclopropenes **92**. The latter can easily be converted into tricyclic furans as a result of recyclization. If BF_3_∙OEt_2_ is used as the catalyst, 2-substitutes furans **93** are selectively formed, while using Cu(II) triflate as the catalyst predominantly leads to 3-substituted counterparts **94** (Scheme 55) [89].

**Scheme 56:**
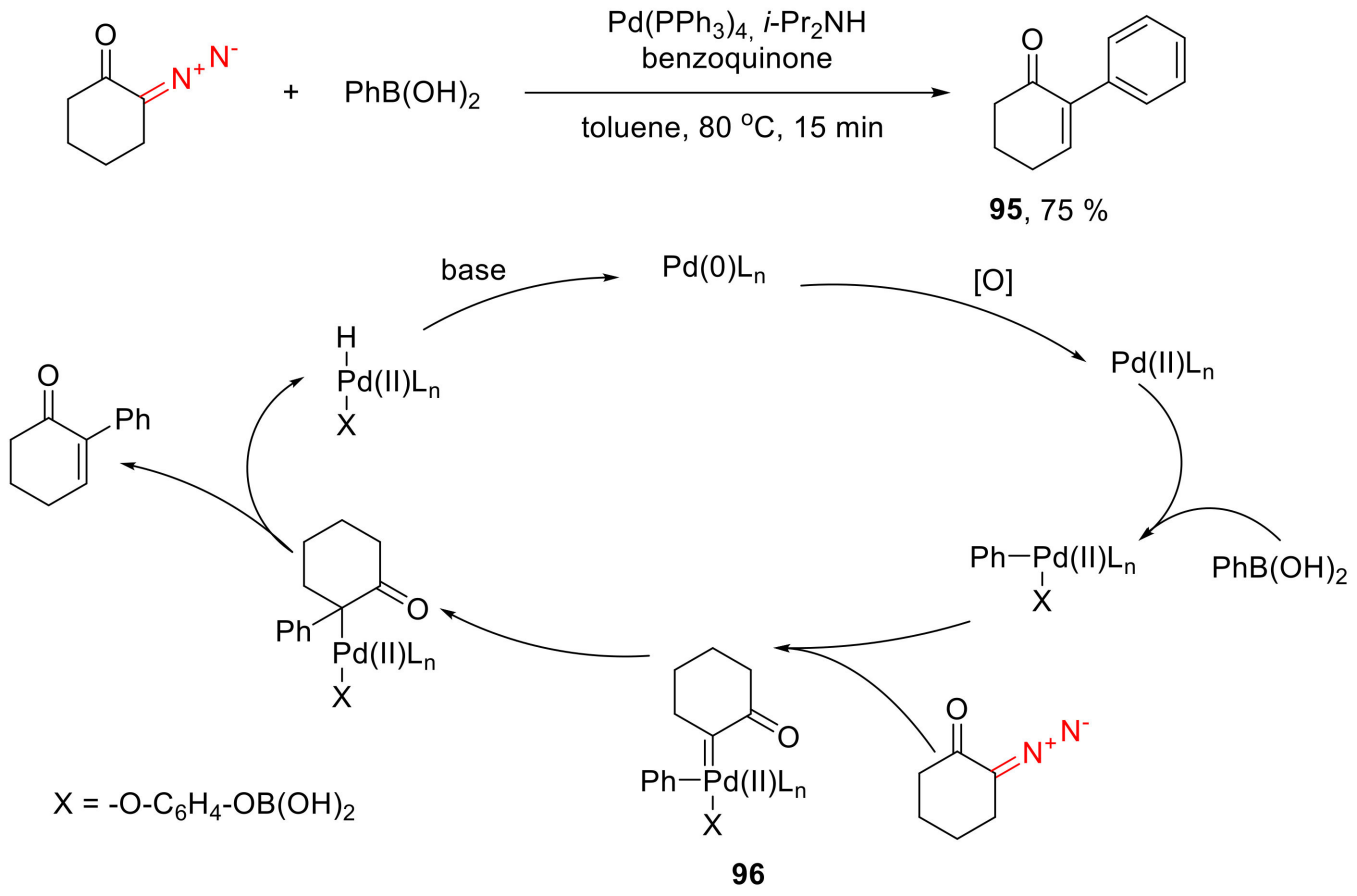
Oxidative Pd-catalyzed cross-coupling of α-diazo ketones with arylboronic acids.

Cyclic α-diazo ketones can undergo Pd-catalyzed oxidative cross-coupling reactions with arylboronic acids to give α-aryl-α,β-unsaturated carbonyl compounds **95**. Although in general such a transformation is well-studied, for cyclic diazo compounds, there are only a handful of examples in the literature [90,91,92]. Instead of arylboronic acids, only without an oxidant, iodoarenes can be used in similar cross-couplings [91]. Mechanistically, the reaction involves the formation of palladium carbene species **96**. Upon migration, within the latter, of the aryl group to the electrophilic carbene carbon atom, β-hydride elimination delivers the observable coupling product **95** (Scheme 56).

**Scheme 57:**
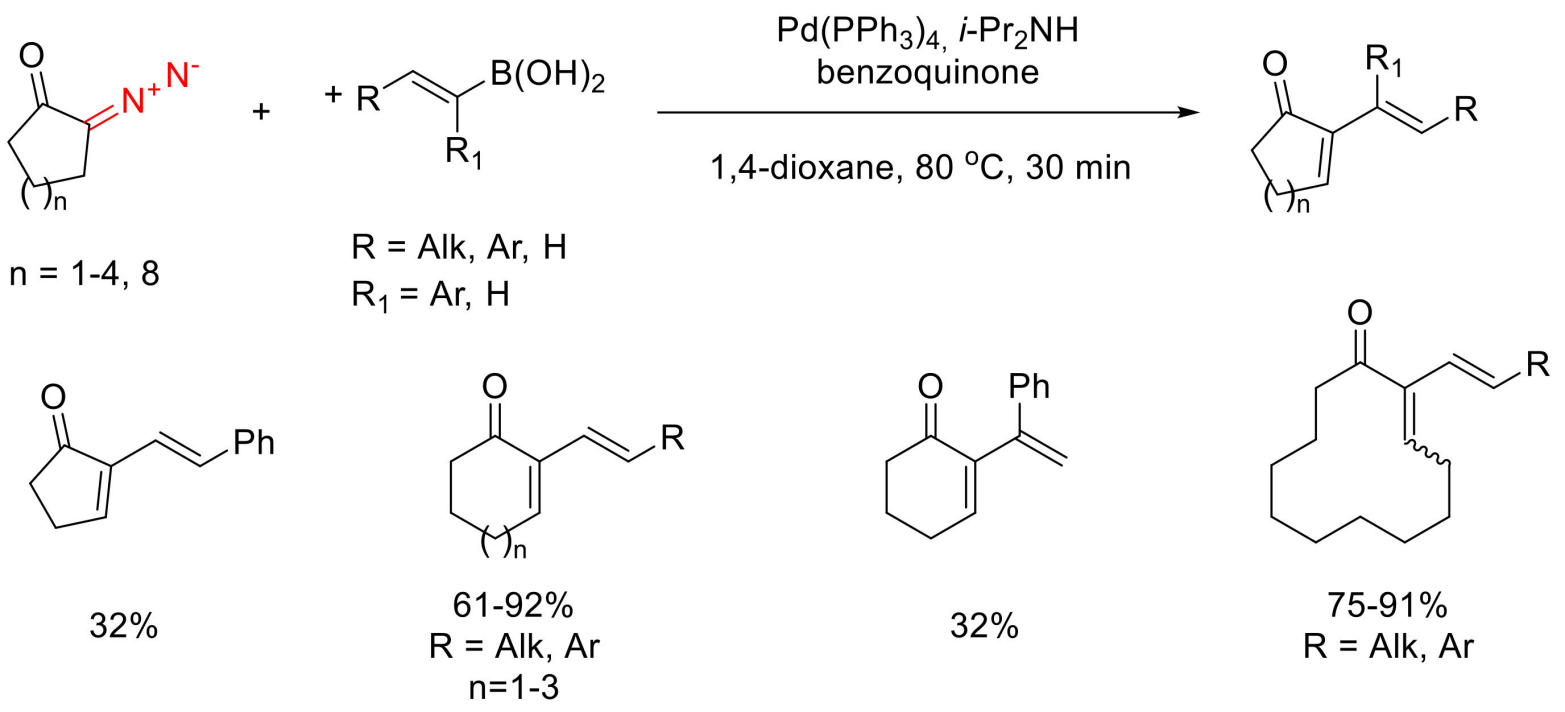
Oxidative coupling of α-diazo ketones with vinyl boronic acids.

Oxidative cross-coupling with α-diazo ketones has been described also for vinyl boronic acids. The transformation has been studied in much detail for a wide range of cyclic α-diazocarbonyl compounds (cyclic α-diazo ketones as well as α-diazo lactones). The reaction demonstrates excellent yields for diazo substrates with more than five atoms in the cycle. With α-diazo cyclopentanone, the yield is a mere 32%. α-Diazo dodecanone is also a competent participant in the reaction. The yield in this case is high, and a sole stereoisomer is obtained; however, the configuration of the double bond has not been established. The same reaction is successful for vinyl boronic acids bearing both aryl and alkyl substituents in the α-position. α-Substituted vinyl boronic acids give much lower yields. The reaction was studied only for *trans*-configured vinyl boronic acids, and the same configuration of the double bond is preserved in the coupling products (Scheme 57) [93].

**Scheme 58:**
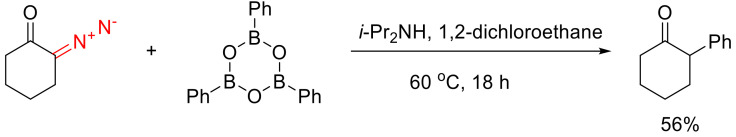
α-Arylation with triphenyl boroxine.

α-Diazo cyclohexanone could be arylated with triphenyl boroxine in the presence of a base to give α-phenyl cyclohexanone (Scheme 58) [94].

### 2.7. 6-Diazo Cyclohex-2-Enones as Precursors to Phenols

Of all cyclic α-diazocarbonyl compounds, 6-diazocyclohex-2-enones occupy a special place, along with their aromatic analogs—β-diazo-α-tetralones. Substituted α-cyclohexenones and α-tetralones, which can be obtained from these precursors, can aromatize under certain circumstances, delivering corresponding phenols and α-naphthols.

**Scheme 59:**
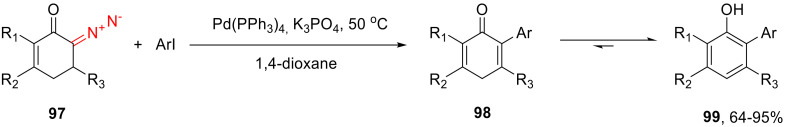
Transformation of 6-diazocyclohex-2-enones **97** into arylphenols **99**.

As was stated above, cyclic α-diazocarbonyl compounds can be arylated by iodoarenes under oxidative cross-coupling conditions. Such a reaction applied to 6-diazocyclohex-2-enone **97** formally gives 2-aryl cyclohexa-2,5-dien-1-ones **98**, which are, essentially, tautomers of the respective arylphenols **99**. A wide scope of diazo cyclohex-5-enones and iodoarenes was studied in this context. The reaction turns out to tolerate both electron-donating and electron-withdrawing substituents in the arylating agent; moreover, o-substituted iodoarenes also work very well in this reaction (Scheme 59) [95].

**Scheme 60:**
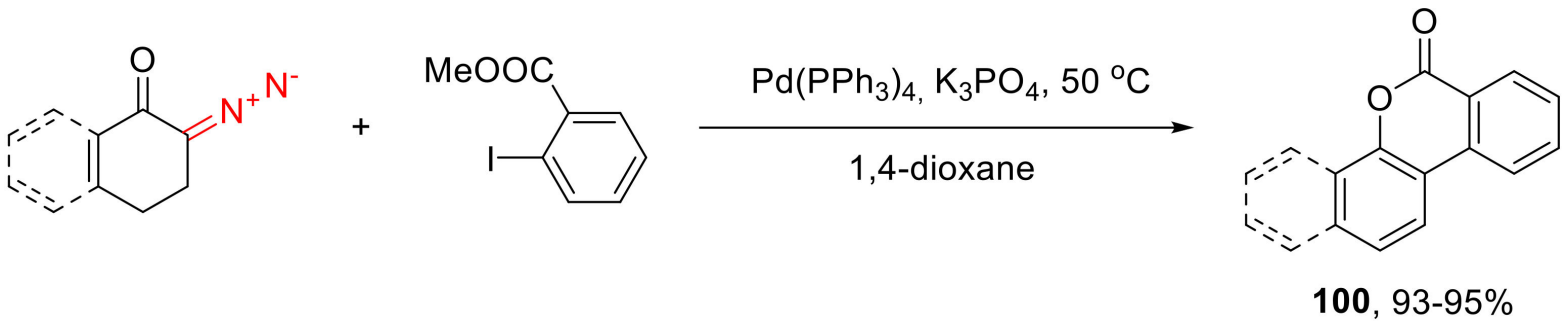
Using *o*-iodobenzoic acid methyl ester as the arylating agent.

When using *o*-iodobenzoic acid methyl ester as the arylating agent, the resulting phenolic adducts undergo spontaneous lactonization into polycyclic scaffolds **100** (Scheme 60) [95].

**Scheme 61:**
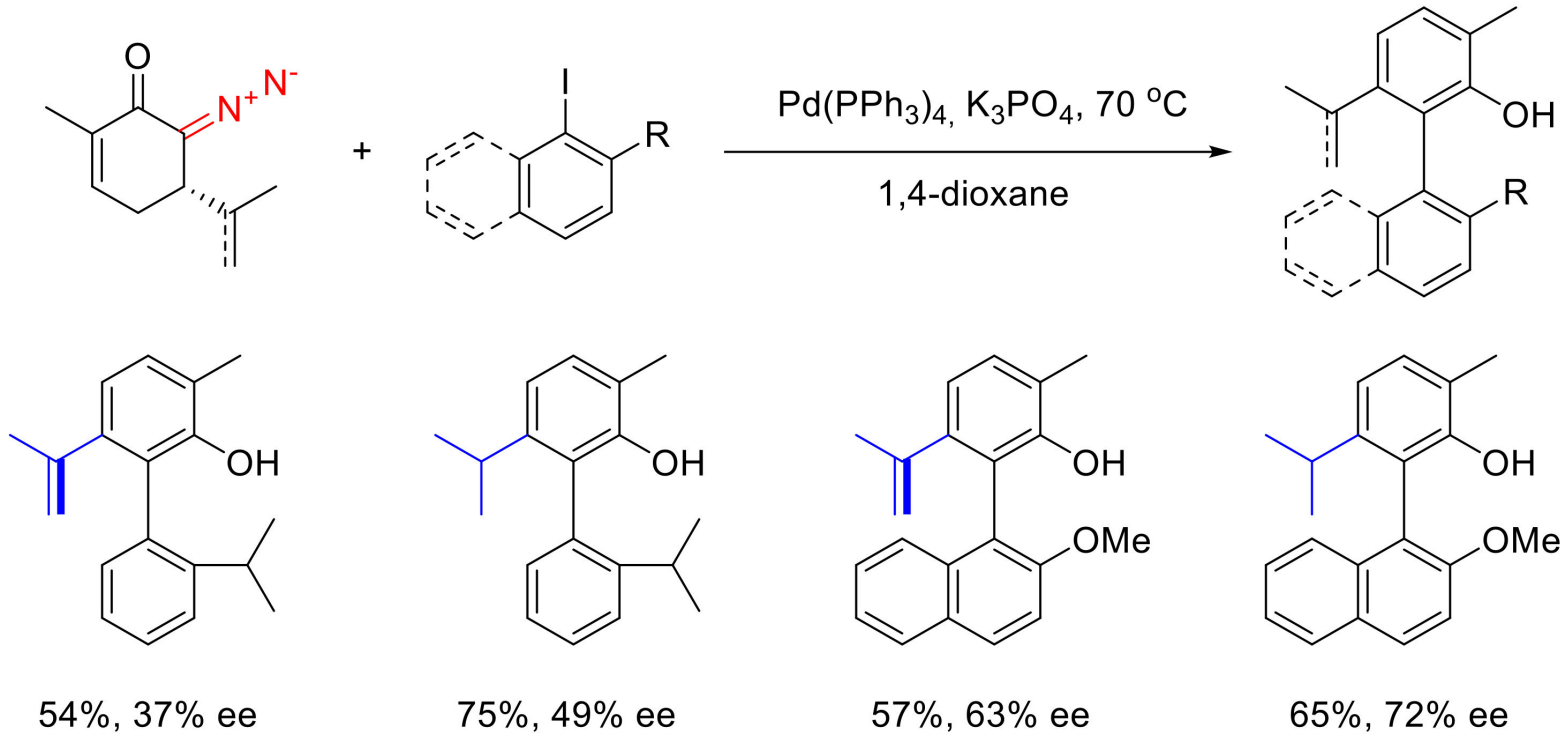
Synthesis of axially chiral biphenyls.

An important aspect of this reaction is that it allows the transfer of chiral information from stereocenters to axially chiral systems. Readily available L-(−)-carvone delivers products with a relatively high enantiopurity with respect to the axis chirality. Replacing the 2-propenyl group with a more sterically demanding isopropyl group in the starting material leads to an increase in both the yield and the enantiomeric purity. Such an approach to enantioselective synthesis of chiral biphenyls holds great promise for the practical synthesis of chiral phosphine ligands and phosphoric acids widely used in enantioselective catalysis (Scheme 61) [95].

**Scheme 62:**
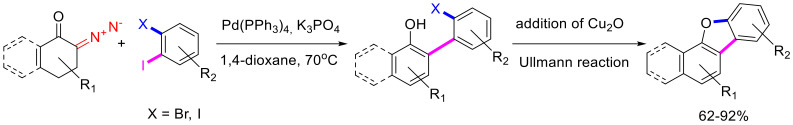
Dibenzofuran synthesis via arylation/aromatization cascade followed by intramolecular Ullmann reaction.

The arylation of 6-diazocyclohex-2-enones can be used for the synthesis of dibenzofurans. In this case, the arylating agents must be various *o*-haloiodoarenes. Once the initial arylation/aromatization is complete, Cu(I) oxide is added into the reaction mixture, and the temperature is raised, which triggers an intramolecular Ullmann reaction. Sometimes, the intramolecular phenol arylation proceeds without any catalyst, albeit in much lower yields (Scheme 62) [96].

**Scheme 63:**
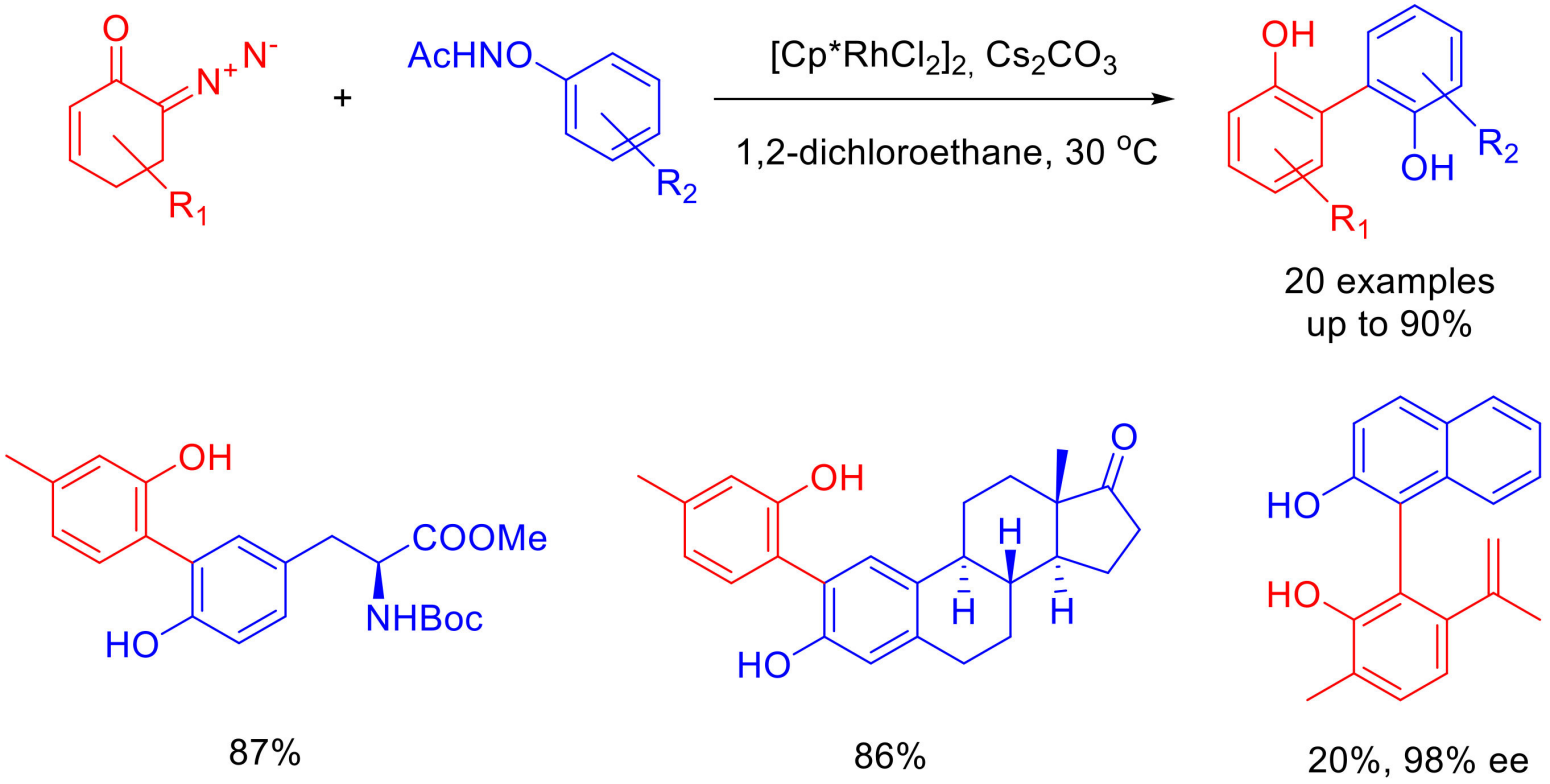
Synthesis of biphenols.

A rather elegant way of synthesizing biphenols from 6-diazocyclohex-2-enones involves C-H functionalization. As the precursors of the second phenol ring, one can use *N*-aryloxyacetamides. The target biphenols are obtained in high yields in the presence of a Rh(III) catalyst and cesium carbonate. The mild conditions allow the building of the biphenol moiety within a range of biologically active compounds. To get to a chiral biphenol, an L-(−)-carvone diazo derivative is used, as described above. The resulting product with *N*-β-naphthoxyacetamide is obtained in a low yield but with excellent enantiopurity (Scheme 63) [97].

**Scheme 64:**
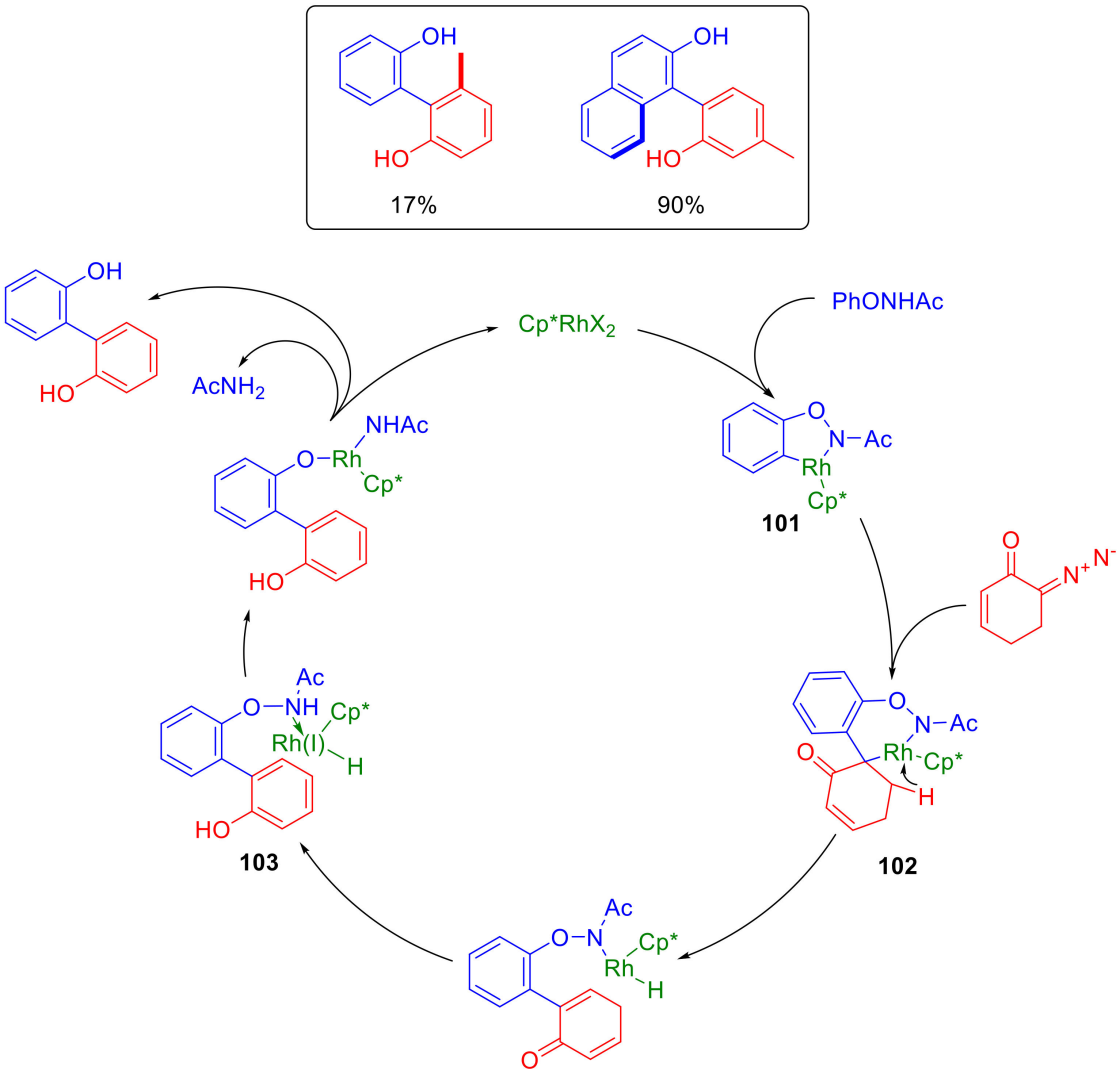
Plausible mechanism for the Rh(III)-catalyzed formation of biphenols.

The following plausible mechanism was proposed for the described biphenol synthesis. *N*-Aryloxyacetamide initially coordinates to the Rh(III) atom, with a subsequent C-H activation and the formation of cyclometallated complex **101**. The latter, upon interaction with the diazo compound, forms a rhodium carbene that undergoes a migratory insertion, forming a second metallocycle (**102**). β-Hydride elimination, aromatization and reductive elimination then follow, which result in the opening of the metallocycle, accompanied by the reduction of the metal to Rh(I). Intermediate **103**, thus formed, undergoes a redox rearrangement, leading to the regeneration of the catalyst. This process is accompanied by the elimination of the target product and acetamide. Apparently, the substituent in position 5 of the starting diazo compound would be oriented towards the sterically encumbered pentamethyl cyclopentadienyl ligand, which explains the low yield obtained with 6-diazo-5-methylcyclohex-2-enone and the L-(−)-carvone derivative. At the same time, using β-naphthoxyacetamide gives a rather high product yield (Scheme 64) [97].

**Scheme 65:**
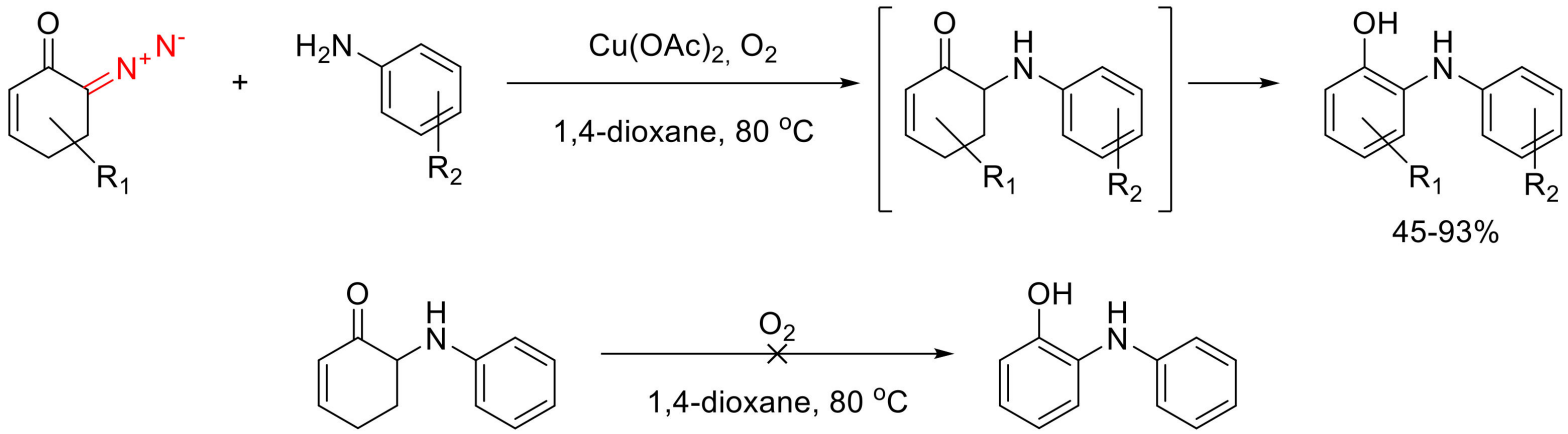
Synthesis of *o*-anilino-substituted phenols from 6-diazocyclohex-2-enones.

*o*-Anilino-substituted phenols can be obtained as the result of a cascade oxidative aromatization of the formal N-H insertion products obtained from 6-diazocyclohex-2-enones and various amines. *o*-Arylamino phenols were obtained in a one-pot protocol using Cu(II) acetate in an oxygen atmosphere [98]. It has been proposed that Cu(I) carbenes were at play [99], which decompose with the action of anilines delivering the N-H insertion product. Cu(I) and Cu(II) species catalyze the final step of the oxidative aromatization as the single-electron transfer agents. (Scheme 65) [98].

**Scheme 66:**
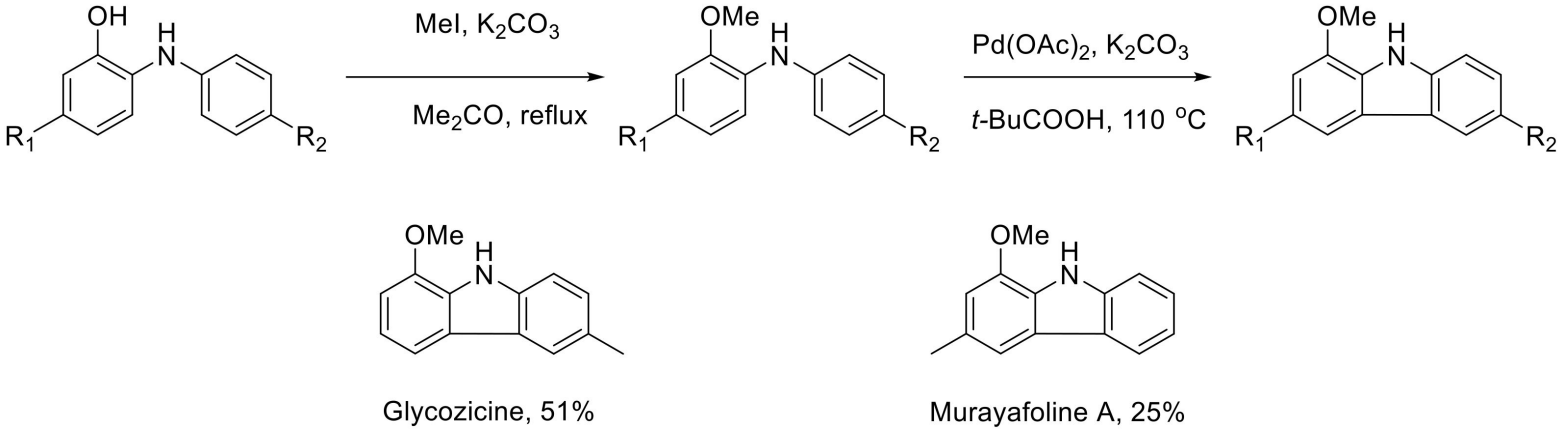
Alkaloid synthesis form *o*-anilino phenols.

In the course of N-H insertion, the anilines act as nucleophiles. Hence, with more electron-rich anilines, the reaction gives better yields. The transformation turns out to be sensitive to the steric properties of the anilines and gives lower yields for *o*-substituted anilines. At the same time, even the presence of a bulky isoprenyl substituent in position 5 of the starting cyclohexanone, does not affect the reaction outcome. The resulting *o*-arylamino phenols are apparent precursors to 1-oxygenated carbazoles, which are present in alkaloids isolated from *Rutaceae* plants. This is demonstrated by the synthesis of two natural alkaloids—glycozoline and murrayafoline A (Scheme 66) [99].

Later, *o*-arylamino phenols were synthesized in one-pot fashion using even milder conditions. Using Pd(II) acetate as the catalyst, the reaction was conducted in an open flask. Similar to the copper catalyst, Pd(II) acetate takes part in two catalytic processes: in the formal N-H insertion, it gives a Pd carbene species, and in the oxidative cycle, it transfers electrons.

This method allows the synthesizing of a series of *o*-arylamino phenols. In this case, all the advantages of the copper-catalyzed method are preserved. However, Pd(II) catalysis allows the involvement of sterically hindered anilines in this reaction without the loss of the yield. For instance, the use of 2,4,6-trimethylaniline gives the target product in 80% yield.

**Scheme 67:**
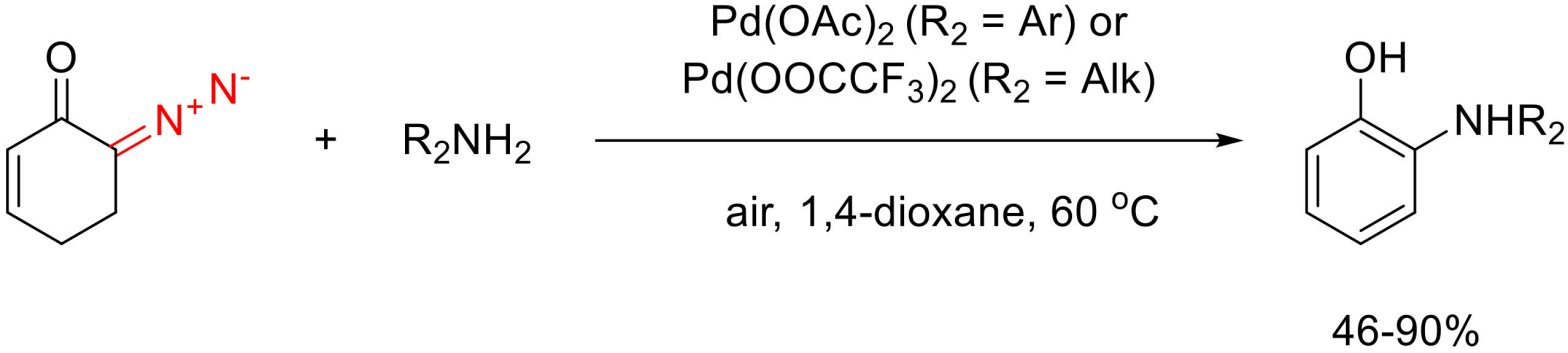
Pd(II)-catalyzed synthesis of *o*-amino phenols from 6-diazocyclohex-2-enone.

Changing the catalyst to Pd(II) trifluoroacetate also allows good yields with aliphatic amines (including sterically demanding *tert*-butylamine and diphenylamino methane), which could not be achieved under copper catalysis. Unfortunately, secondary aliphatic amines do not work in these reactions (Scheme 67) [100].

**Scheme 68:**
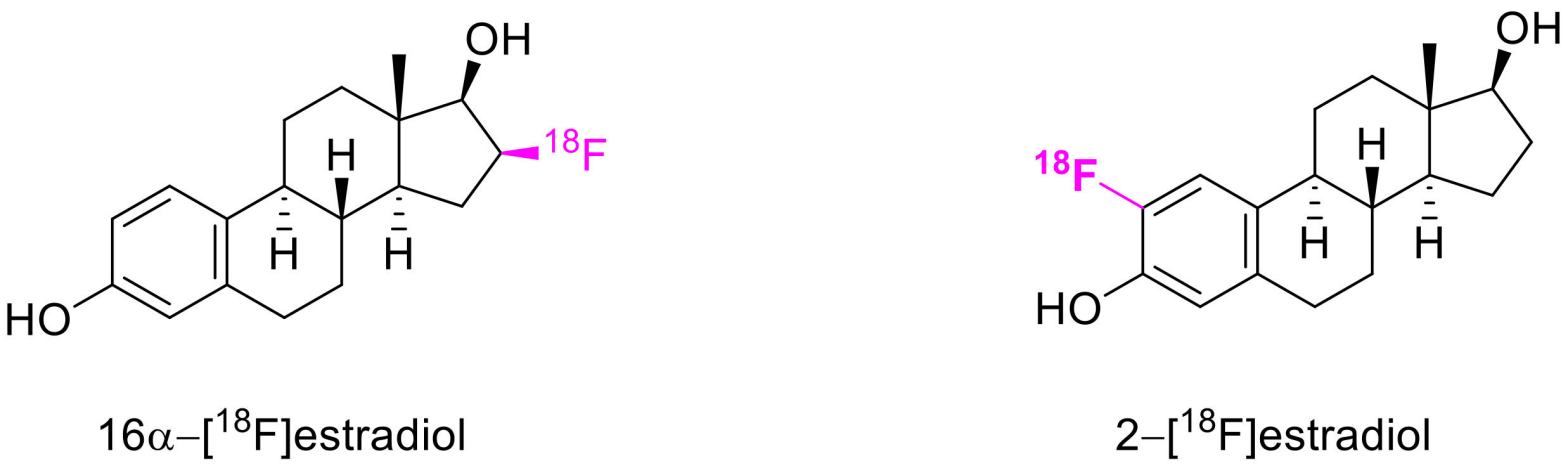
Structures of two ^18^F PET tracers.

The synthesis of phenols from 6-diazocyclohex-2-enones has tremendous potential in radiopharmacology. Today, one of the commonly used positron emission tomography (PET) tracers is 16α-[^18^F]fluoroestradiol. At the same time, its analog, 2-[^18^F]fluoroestradiol, containing an *o*-fluorophenol moiety, has a significantly higher practical potential, as it more strongly binds to steroid carrier proteins (Scheme 68). The existing synthesis method of this compound does not conform to the demands of radiopharmacology, as the hot fluorine is introduced two steps prior to the completion of its synthesis [101]. Synthesizing 2-[^18^F]fluoroestradiol through the fluorination of estradiol with electrophilic fluorinating agents is also not an option, as those require the use of ^19^F_2_ as the career gas, which leads to very low isotopic enrichment.

**Scheme 69:**
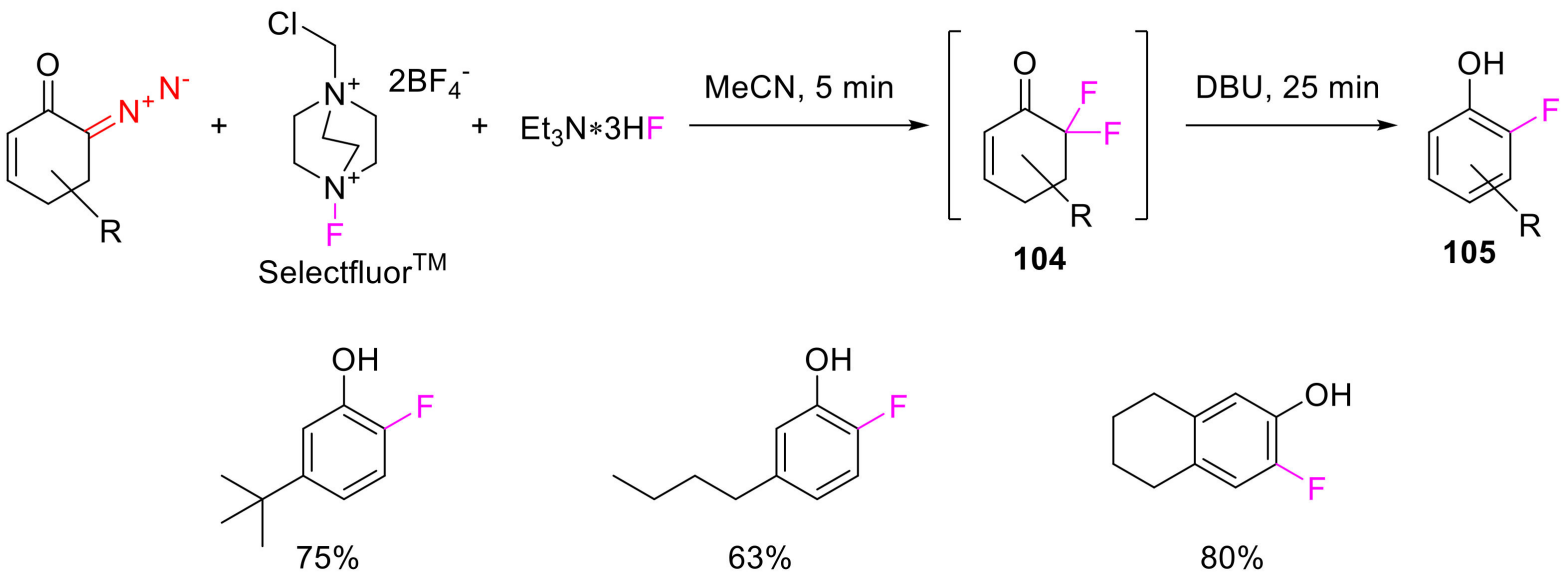
Synthesis of monofluorophenols **105** from 6-diazocyclohex-2-enones.

6-Diazocyclohex-2-enones can enter a halofluorination reaction with the simultaneous use of electrophilic and nucleophilic fluorinating agents. The resulting 6,6-difluorocyclohex-2-enones **104** are treated with a base (DBU), which result in dehydrofluorination and the formation of monofluorophenols **105** in high yields (Scheme 69) [102].

**Scheme 70:**
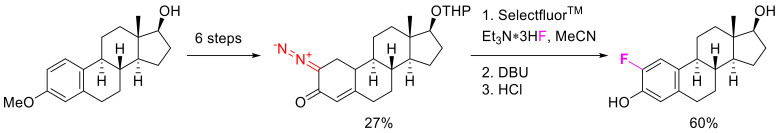
Synthesis of 2-fluoroestradiol from *O*-methyl estradiol.

Using this method, 2-fluoroestradiol is obtained in nine steps from readily available *O*-methyl estradiol. After the isotope fluorine is introduced, only two rather simple steps follow, which makes this approach suitable for producing 2-[^18^F]fluoroestradiol in the demanding environment of a PET scan laboratory (Scheme 70).

**Scheme 71:**
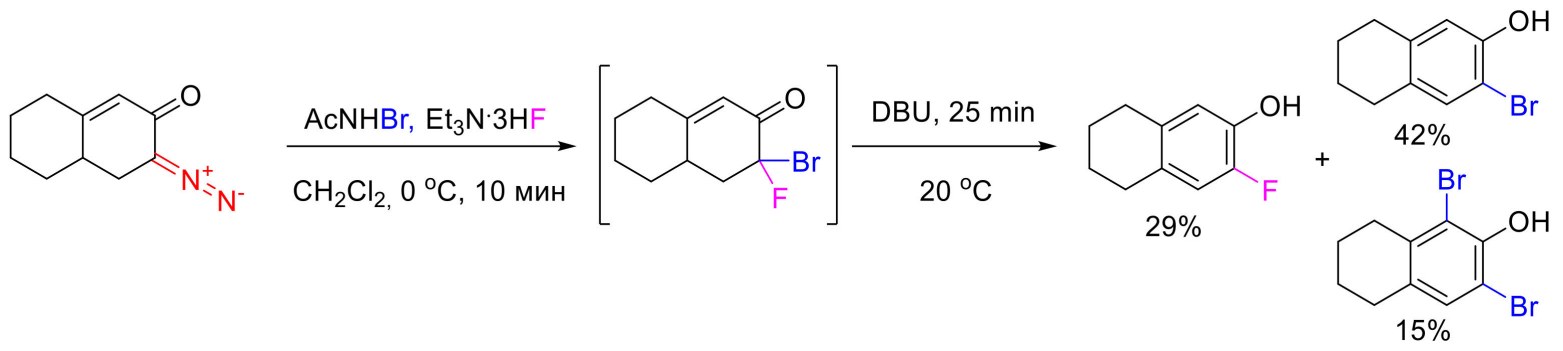
Synthesis of monofluorophenols via bromofluorination.

The only drawback of the approach based on the initial difluorination and subsequent HF elimination is the need for the use of an electrophilic fluorinating agent. The low isotope enrichment of the latter results in even lower enrichment of the product. To circumvent this problem, an alternative approach based on halofluorination and subsequent HHal elimination was proposed. Reactions with various electrophilic halogenating agents were screened, and the best results were obtained with *N*-bromoacetamide (Scheme 71).

A serious drawback of this approach, compared to difluorination, is the low yields of the monofluorophenols due to elimination of HF and the formation of numerous side-products resulting from electrophilic aromatic bromination. At the same time, when [^18^F]-labeled triethylamine hydrofluoride is used, bromofluorination has one key advantage, namely, the target *o*-fluorophenol is formed with very high isotope enrichment [102].

## 3. α-Diazo Lactones

### 3.1. Methods of Synthesis

**Scheme 72:**
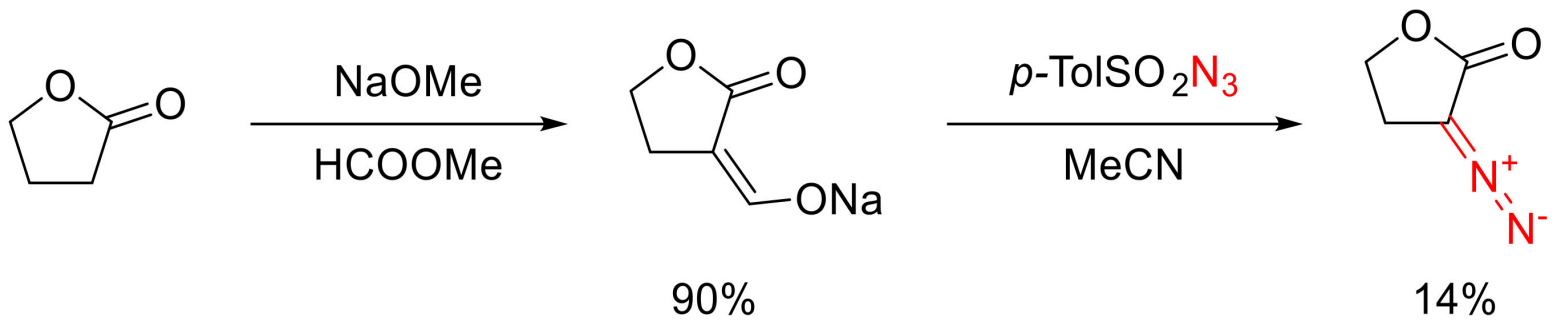
The first synthesis of α-diazo-γ-butyrolactone.

For the first time, α-diazo-γ-butyrolactone was synthesized through formylation and subsequent diazo transfer. Unfortunately, the yield of the target diazo compound was rather low (Scheme 72) [103,104].

**Scheme 73:**
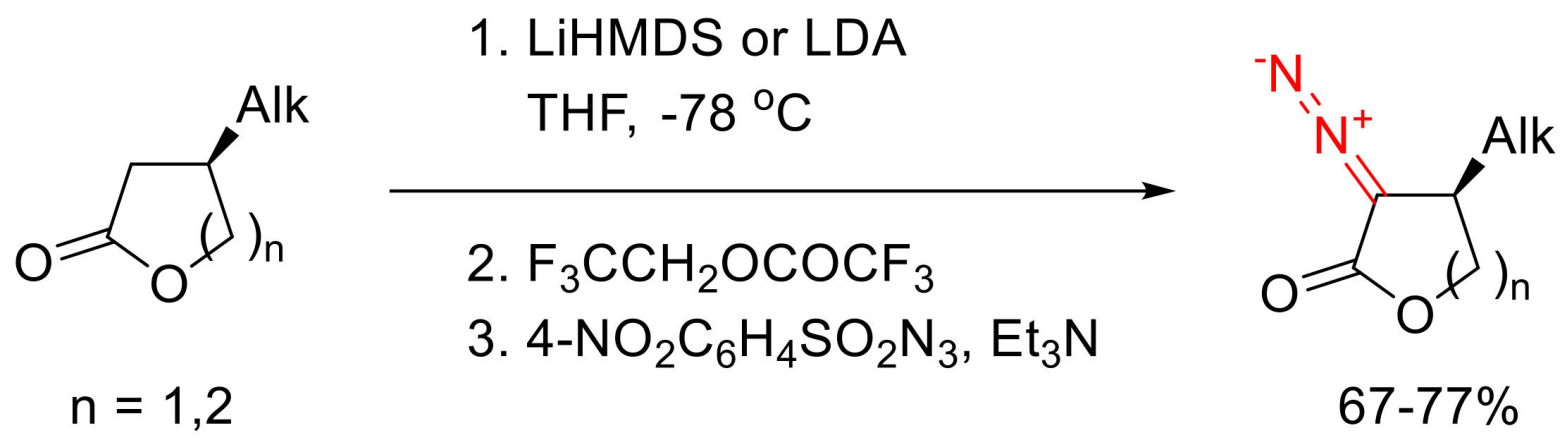
Detrifluoroacetylative diazo transfer as the method for the preparation of α-diazo lactones.

Later, it was shown that various α-diazo-γ-butyrolactones can be synthesized in direct diazo transfer conditions at low temperatures. However, this method did not gain popularity because of the formation of large numbers of triazine and azide side-products [105,106,107,108,109,110,111,112,113,114,115,116,117,118]. Much better results were achieved using detrifluoroacetylating diazo transfer, which to-date is the most widely accepted method of synthesizing α-diazo lactones, including six- and seven-membered ones (Scheme 73) [5,96,109,110].

**Scheme 74:**
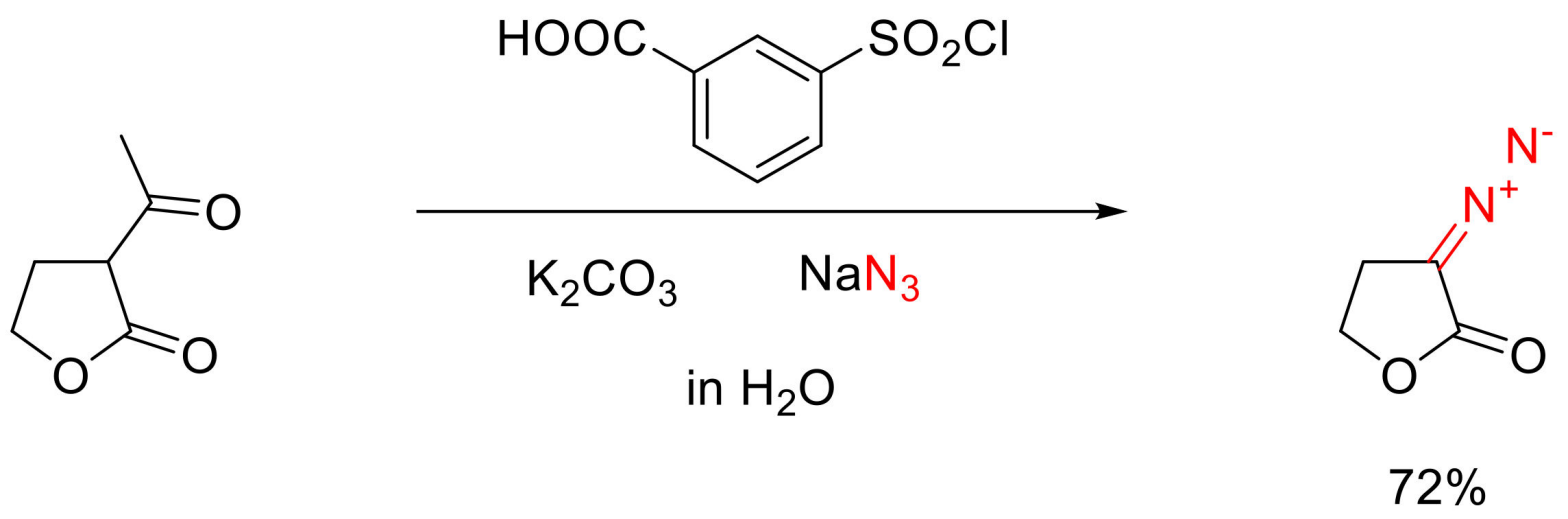
Deacetylative diazo transfer using the SAFE diazo transfer protocol in an aqueous medium.

α-Diazo-γ-butyrolactones can also be obtained from α-acetyl γ-butyrolactones. In this case, the diazo transfer reaction is usually conducted using trifluoromethane sulfazide generated in situ under phase transfer conditions [111,112,113]. In addition, later it was shown that the practical protocol of SAFE diazo transfer (vide supra) is also applicable for deacetylative diazo transfer with good yield (Scheme 74) [114].

**Scheme 75:**
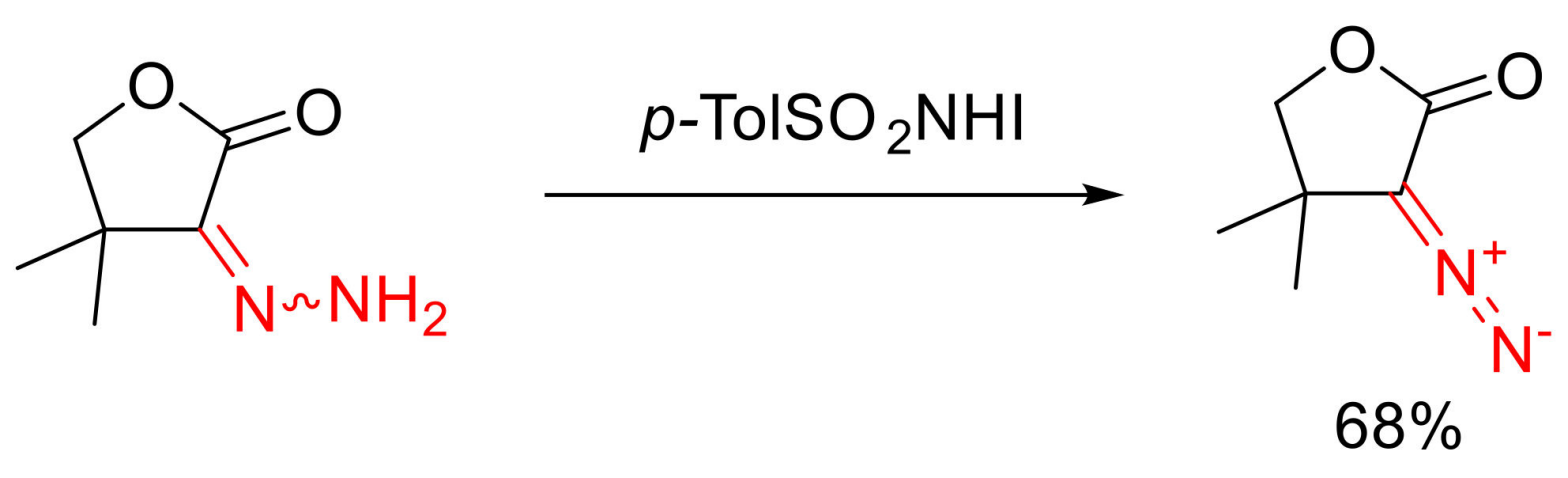
Oxidation of a hydrazone.

α-Diazo-γ-butyrolactones can be obtained from the respective hydrazone by oxidizing it with *N*-iodo-*p*-toluenesulfonamide (Scheme 75) [115].

**Scheme 76:**
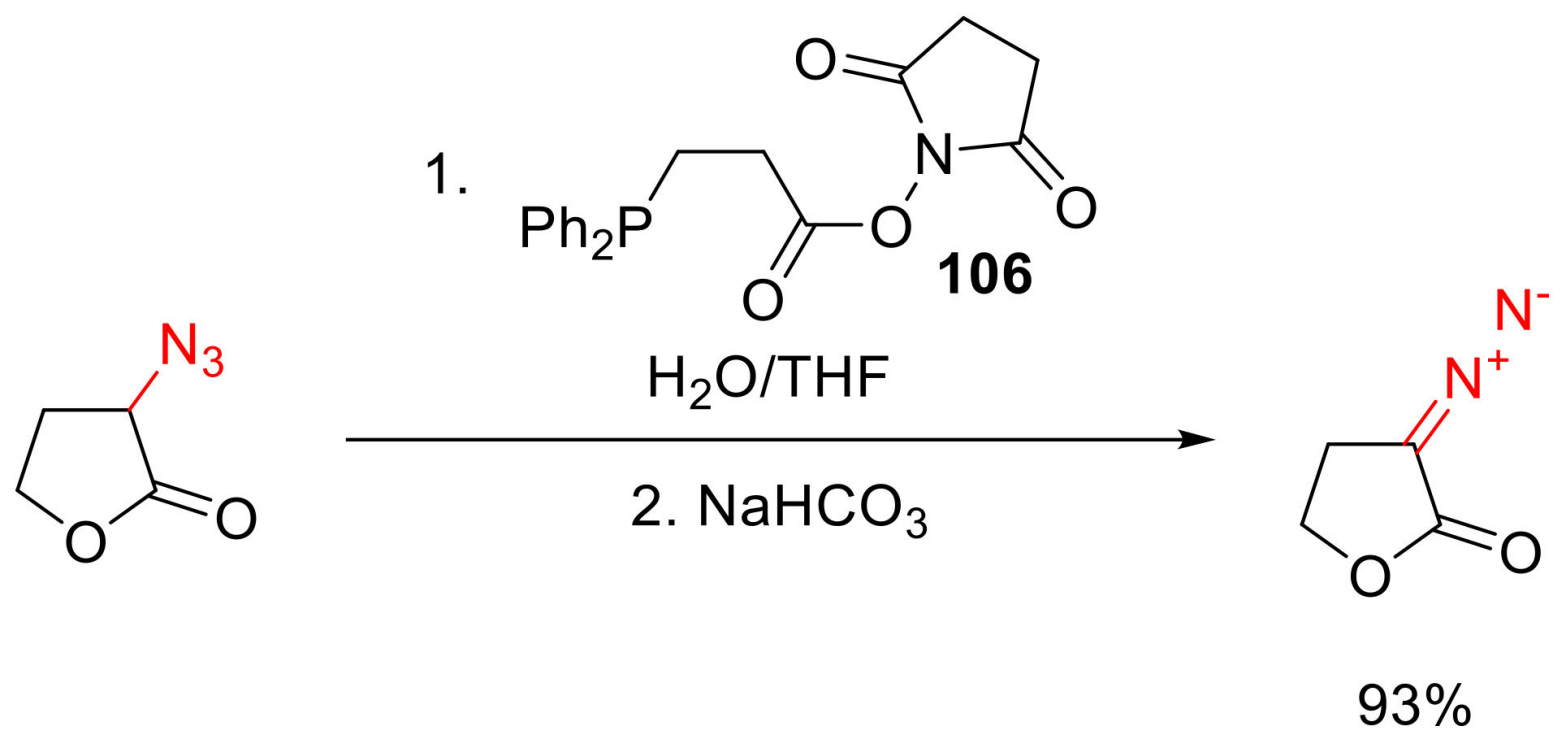
Azide reduction with NHS-containing phosphine.

α-Diazo-γ-butyrolactones can be obtained from the respective azide in the presence of a phosphine carrying a succinimide ester function in one of its side chains (such as **106**). The mild reaction conditions ensure the tolerance of many functional groups, which make this method, in particular, very suitable for the synthesis of various α-diazo-γ-butyrolactones (Scheme 76) [116].

Six- and seven-membered α-diazo-β-hydroxylactones, as well as their keto-analogs, can be obtained via intramolecular aldol condensation of the suitable terminal diazo esters [60,117].

### 3.2. Decomposition of Cyclic α-Diazo Lactones Followed by β-Migration

**Scheme 77:**
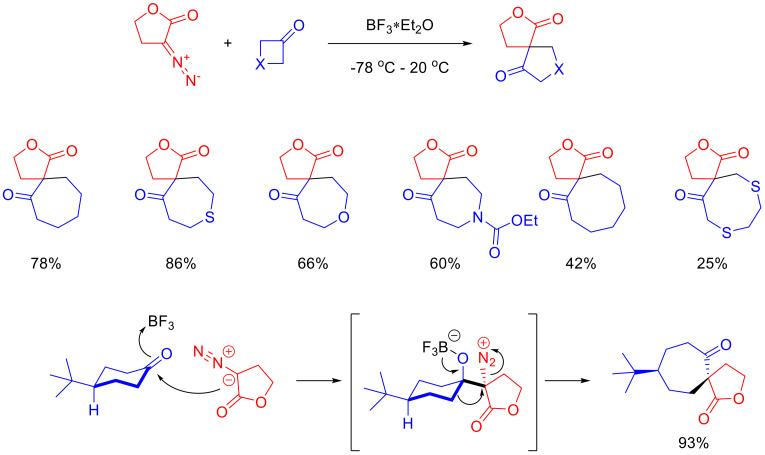
Büchner–Curtius–Schlotterbeck reaction of α-diazo-γ-butyrolactones.

α-Diazo-γ-butyrolactones can enter the Büchner–Curtius–Schlotterbeck reaction with cyclic ketones, which results in spirocyclic lactones. In the presence of Lewis acid catalysts, the nucleophilic addition of a diazo lactone to the ketone carbonyl group takes place, followed by the elimination of the nitrogen molecule and Tiffeneau–Demjanov rearrangement. The reaction scope is limited to six- to eight-membered cyclic ketones. Interestingly, when 4-(*tert*-butyl)cyclohexanone is used as a substrate, the reaction proceeds with high diastereoselectivity. This could be rationalized with the fixation of the cyclohexanone conformation by the sterically demanding substituent and the stereochemical requirements for the a-migration process, accompanied by the nitrogen molecule elimination (Scheme 77) [114].

**Scheme 78:**
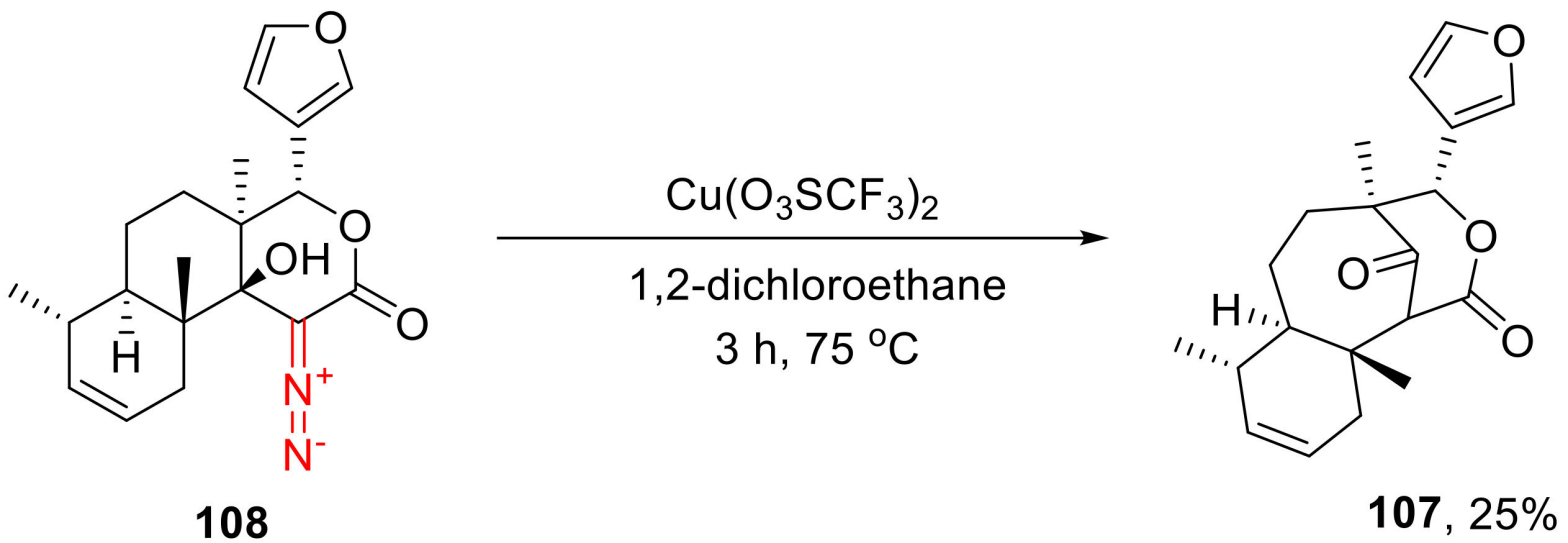
Cu(II) triflate catalyzed decomposition and rearrangement of tricyclic α-diazo-β-hydroxyvalerolactone **108**.

The decomposition of α-diazo-β-hydroxylactones can be accompanied by a β-alkyl shift that leads to 1,3-dicarbonyl compounds. This transformation has only once been exemplified in the literature in the context of cage-structure synthesis. A Cu(II) triflate catalyzed reaction gave a low yield of the product of decomposition and rearrangement **107** of tricyclic α-diazo-β-hydroxyvalerolactone **108** (Scheme 78) [117].

**Scheme 79:**
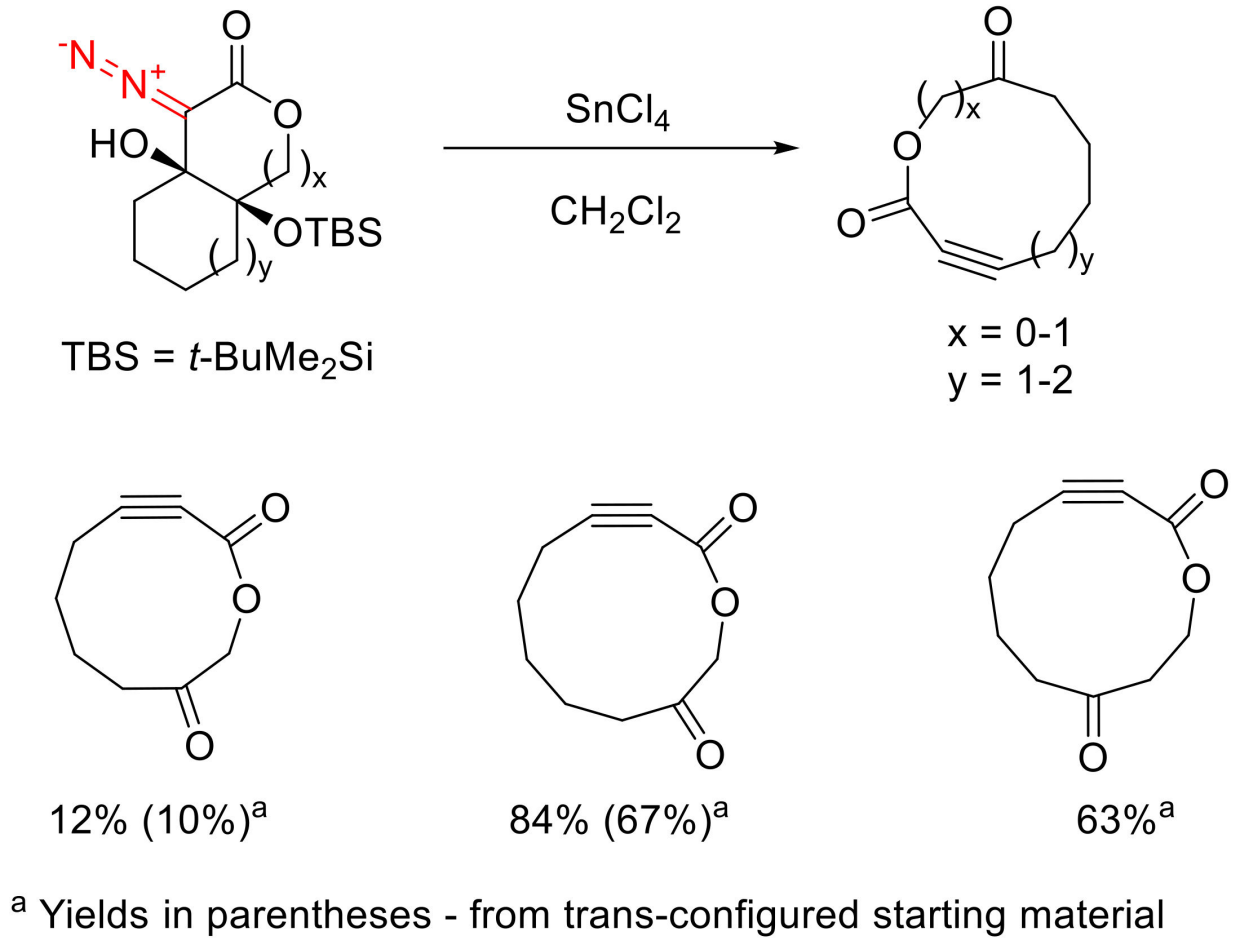
Fragmentation of α-diazo-β-hydroxy-γ-silyloxylactones into macrocyclic products.

Similar to the keto-analogs, bicyclic α-diazo-β-hydroxy-γ-silyloxylactones can undergo fragmentation upon the action of Sn(IV) chloride. Unfortunately, for lactones, this transformation is much less effective than for ketones. Similar to ketones, macrocyclic products are obtained with better yields from cis-bicyclic lactones compared to their trans-counterparts (Scheme 79) [118].

### 3.3. Decomposition of α-Diazo Lactones in the Presence of Substrates Containing n-Nucleophiles

The first attempts to conduct a formal X-H insertion using α-diazo-γ-butyrolactones were undertaken using Brǿnsted acids as catalysts; however, the target products were obtained with mediocre yields [104].

**Scheme 80:**
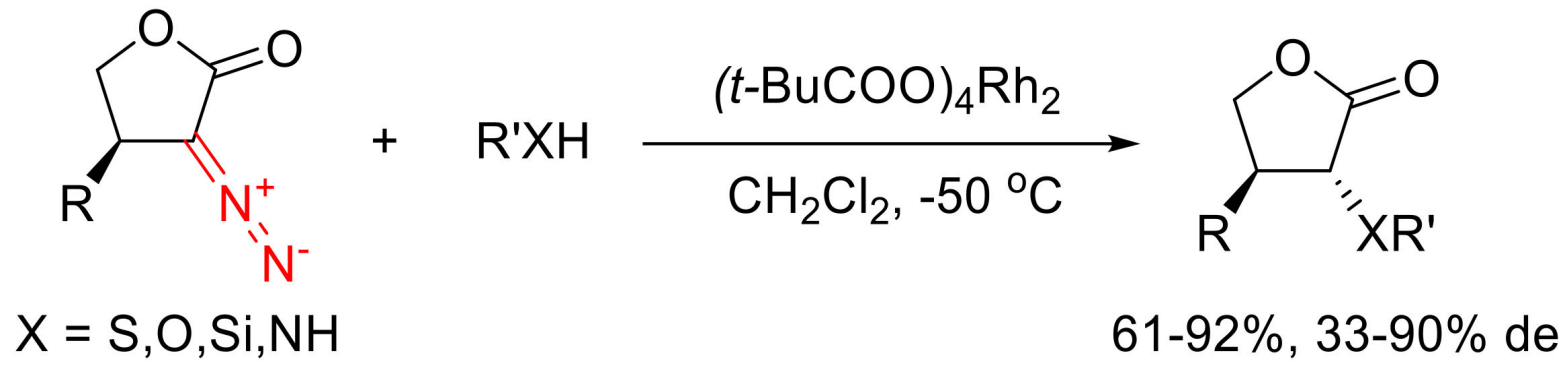
Formal insertion of α-diazo lactone-derived Rh(II) carbenes into various X-H bonds.

As to the similar reactions catalyzed by transition metals, it is important to go into the fundamentals of the reactivity of α-diazo lactones. It was established that five- and six-membered α-diazo lactones at low temperature in the presence of a Rh(II) catalyst can undergo a wide spectrum of intramolecular transformations in high yields, even in the case of α-substituted substrates. In particular, within a limited number of examples, it has been shown that β-substituted α-diazo-γ-butyrolactones can enter reactions of formal insertion into S-H, N-H, O-H and Si-H bonds (Scheme 80). The diastereoselectivity of these transformations has not always been high; however, in all cases the *trans*-isomer was predominantly formed. The *trans*-isomer content could be increased after the completion of the reaction by epimerizing the reaction mixture in the presence of a base [5].

**Scheme 81:**
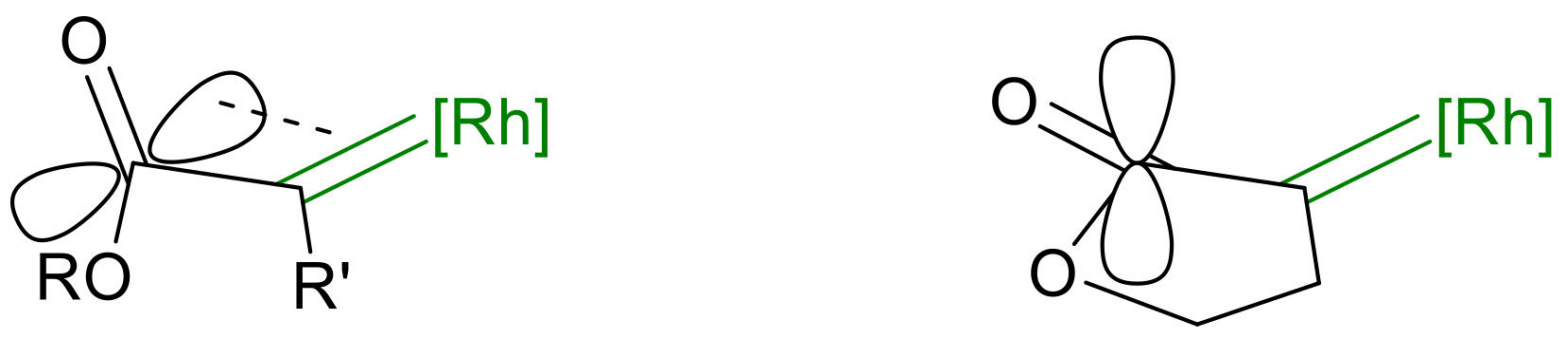
Comparison of α-diazo esters and α-diazo lactones in molecular orbital orientation.

The uniqueness of these transformations is in the fact that the acyclic analogs of α-diazo lactones—α-diazo esters containing a methine group in γ-position—after the formation of the carbene, simply undergo a β-hydride shift, which practically excludes intermolecular reactions. Quantum chemical calculations rationalized this difference in the chemical behavior of α-diazo lactones and α-diazo esters by the different steric properties of the carbenes formed. The carbenes formed from α-diazo esters are stabilized through the interaction with the π* orbital of the carbonyl group, which results in the orthogonal orientation of the carbonyl group and the C-Rh bond. As a result, for steric reasons, the approach of another reacting molecule is blocked, and the carbene finds a way towards stabilization via the β-hydride shift with an olefin formation. In the case of α-diazo lactones, conformational rigidity prevents the orthogonal orientation of the carbonyl group and C-Rh bond, which facilitates any intermolecular reactions (Scheme 81). This hypothesis is supported by the fact that, in contrast to five- and six-membered α-diazo lactones, less rigid α-diazo-β-ethylcaprolactones cannot be involved in intramolecular reactions [5].

**Scheme 82:**
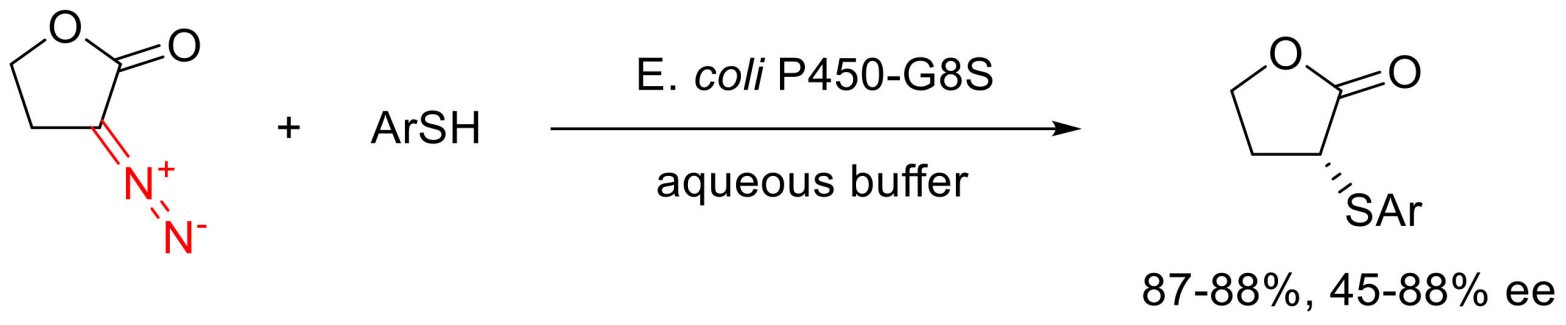
Cytochrome-catalyzed S-H insertion reaction of α-diazo-γ-butyrolactones.

Along with Rh(II) carboxylates, the X-H insertion reaction of α-diazo lactones can be catalyzed by gem-containing enzymes. The main advantages of such catalysts are their high enantioselectivity and their high reaction rate. For example, a cytochrome modification was found that turned out to be capable of effectively catalyzing the insertion reaction of α-diazo-γ-butyrolactones into aromatic thiols with high enantioselectivity (Scheme 82). Fine-tuning of the enzymes’ catalytic properties can be achieved by varying the gem-binding amino acid. In particular, going from cytochrome P411 to cytochrome P450 (essentially, changing from serine to cysteine), the catalytic activity and the enantioselectivity of the catalyzed reaction grows more than five-fold [119].

**Scheme 83:**
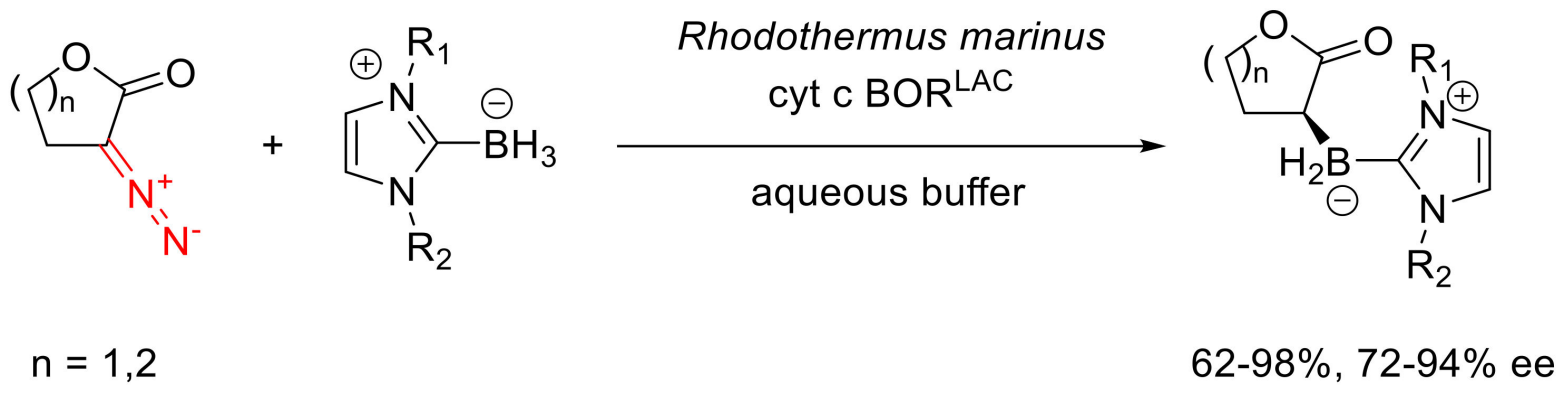
Cytochrome-catalyzed B-H insertion reaction of five- and six-membered α-diazo lactones.

Using the cytochrome catalysis, it is possible to obtain B-H insertion products with high enantioselectivity in reactions with boranes and five- and six-membered α-diazo lactones (Scheme 83). Notably, the same reaction with α-diazo caprolactones gives a very low yield. Since the free borane is a reactive compound, unstable in an aqueous medium, its complexes with *N*-heterocyclic carbenes are used in the reaction. This type of stabilization is dictated by the high biological compatibility of imidazolium salts and their derivatives [110].

**Scheme 84:**
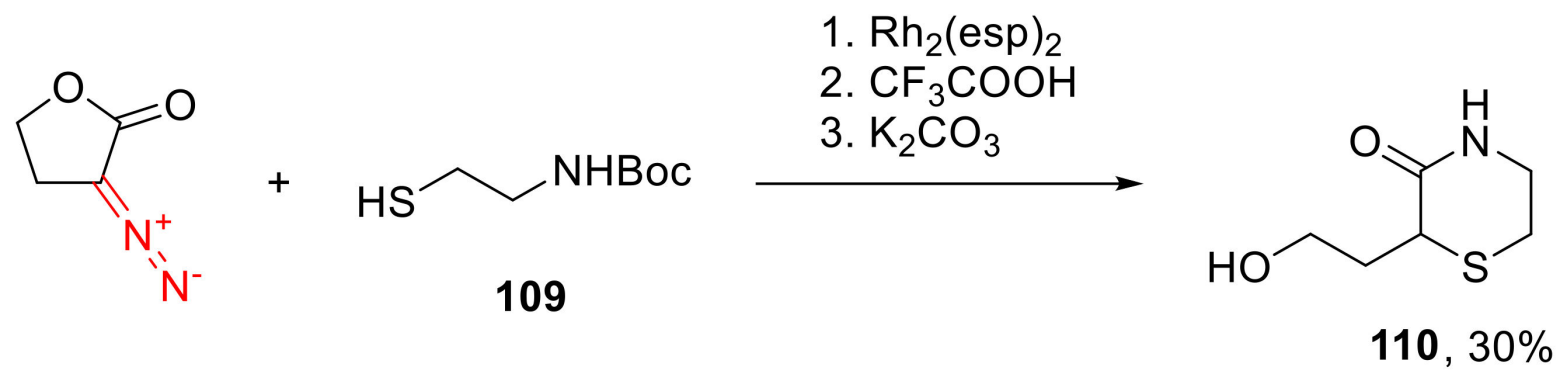
Cascade Rh(II)-catalyzed S-H insertion—recyclization.

In some cases, X-H insertion products derived from α-diazo lactones can be postmodified. For example, the product of a Rh(II)-catalyzed S-H insertion into *N*-Boc-aminoethanethiol (**109**) can undergo recyclization into hydroxyethyl-substituted thiomorpholinone **110** (Scheme 84) [120].

**Scheme 85:**
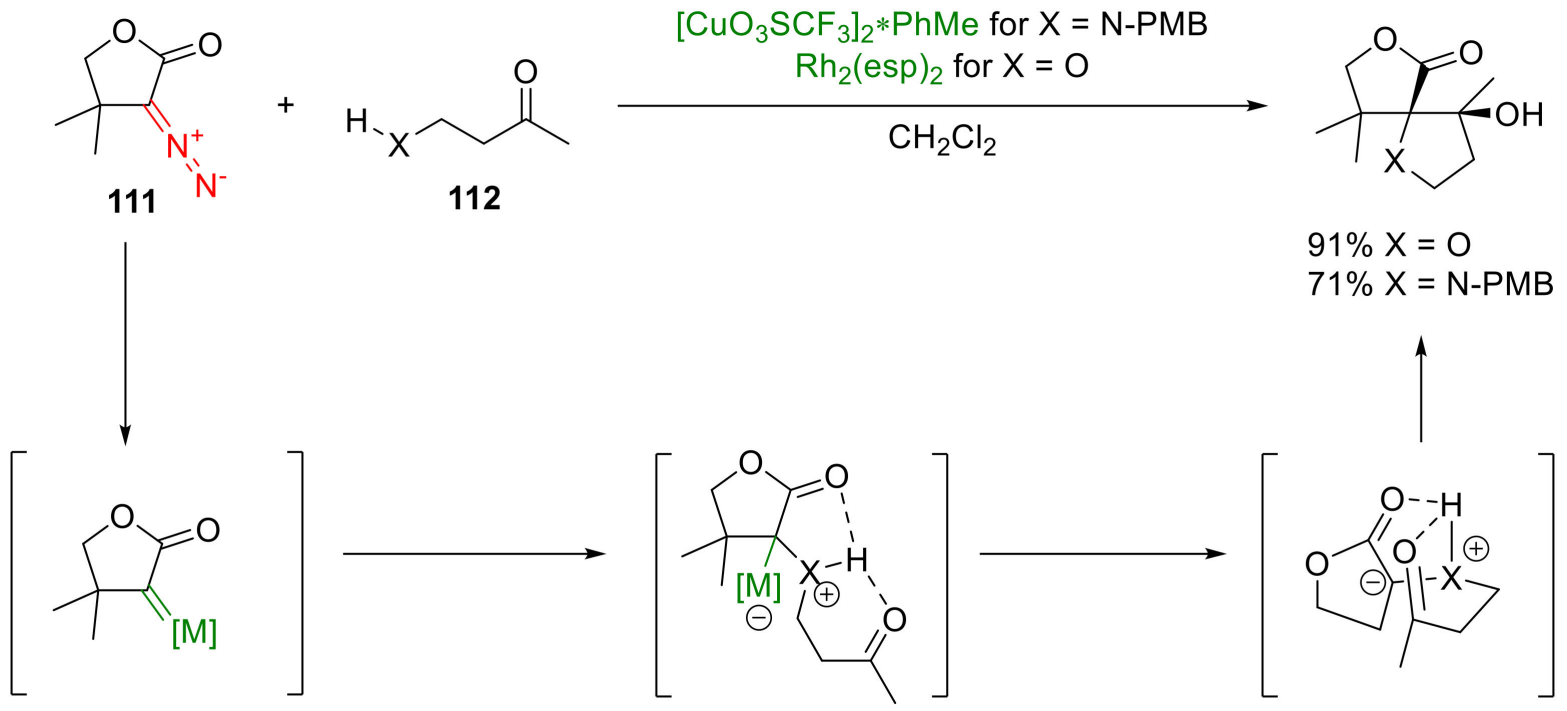
Spirocycle synthesis via X-H insertion into the molecule bearing an electrophilic moiety.

The ylide formed in the course of the X-H insertion reaction, instead of undergoing protolytic migration, can find stabilization through the interaction with an electrophile. If both the nucleophilic and electrophilic centers are within the same molecule, the reaction delivers spirocyclic compounds. This is nicely illustrated by the transition metal-catalyzed decomposition of α-diazo-γ-butyrolactones **111** in the presence of 3-hydroxy- and 3-alkylamino ketones **112** (Scheme 85). The diastereoselectivity of the reaction can be rationalized by the fixed ylide conformation due to hydrogen bonding between the amine or alcohol proton and the two carbonyl groups [121,122].

**Scheme 86:**
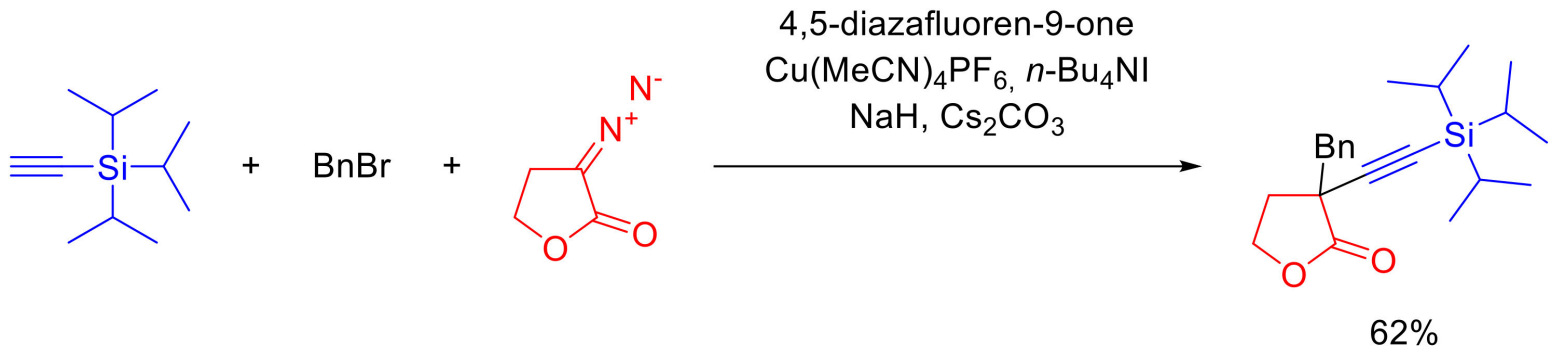
Ylide trapping by alkyne and alkyl bromide.

An unusual variant of ylide trapping represents the sequential interaction of α-diazo-γ-butyrolactones with an acetylene and alkyl bromide in the presence of Cu(I) acetonitrile complex (Scheme 86) [113].

Supposedly, the reaction proceeds through an acetylenide Cu(I) complex that decomposes the diazo lactone, which results in the respective carbene. After the alkynyl migration, the organocopper intermediate formed is alkylated in the presence of the base, delivering the observable final product [113].

### 3.4. Other Reactions of α-Diazo Lactones

**Scheme 87:**
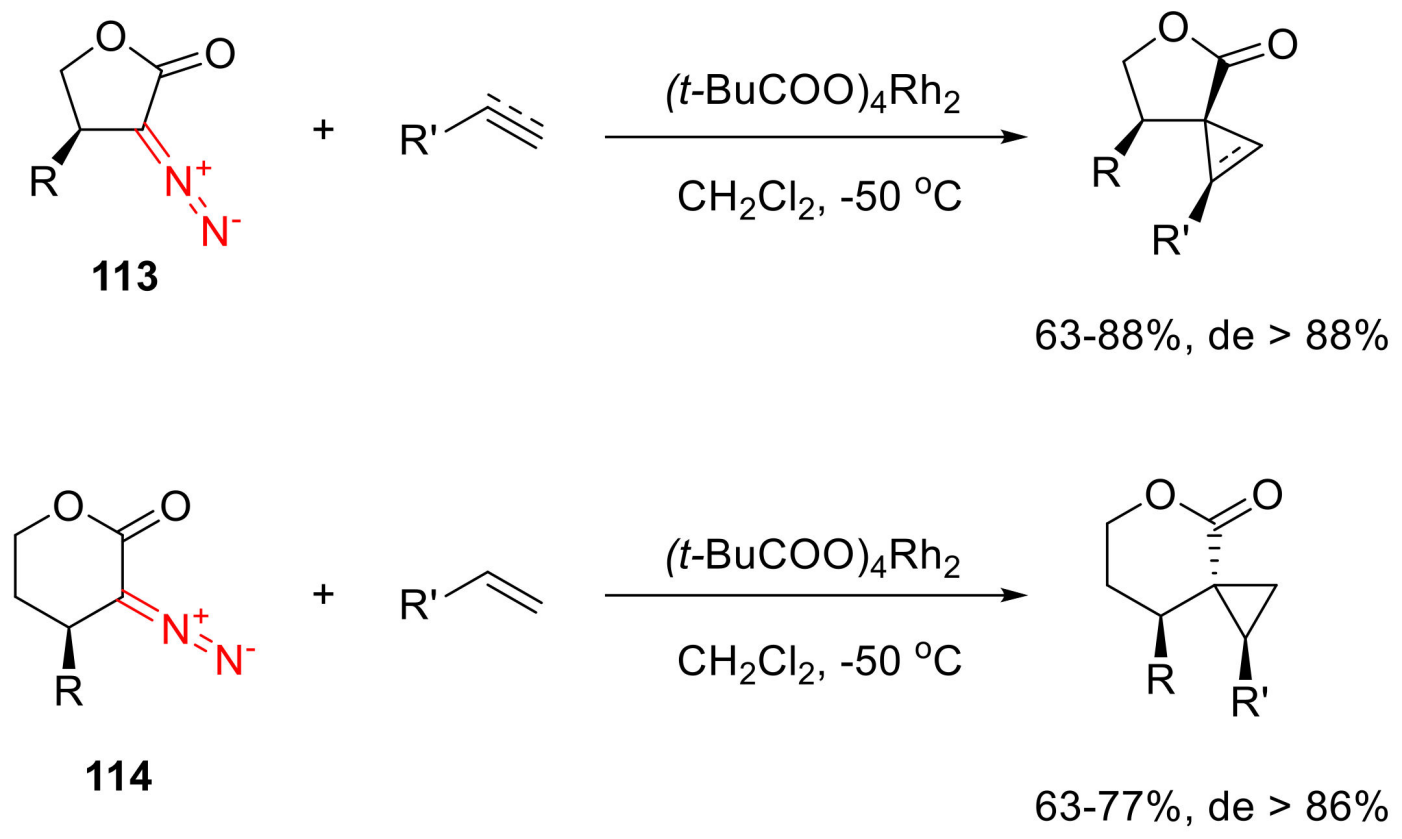
Cyclopropanation and cyclopropenation reactions of α-diazo lactones **113**–**114**.

Five- and six-membered α-diazo lactones **113**–**114** react with olefins and terminal acetylenes delivering cyclopropanes and cyclopropenes in high yields. In the case of a substitution in the γ-position of **113**–**114**, the reaction proceeds diastereoselectively. The relative configuration of the chiral center in the β-position strongly depends on the ring size of the starting α-diazo lactone (Scheme 87) [5].

**Scheme 88:**
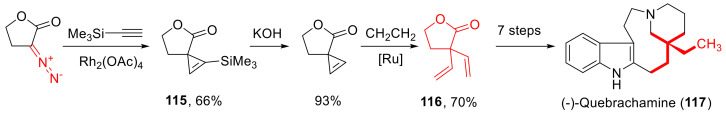
Rh(II)-catalyzed cyclopropenation of trimethylsilyl acetylene en route to (−)-quebrachamine (**117**).

A cyclopropenation reaction of α-diazo-γ-butyrolactones has been reported, even for trimethylsilyl acetylene. In the cyclopropane product **115**, the silyl group was removed, and the cyclopropane ring was opened. The resulting α,α-divinyl-γ-butyrolactone **116** served as the key intermediate in the synthesis of (–)-quebrachamine (**117**) (Scheme 88) [107].

**Scheme 89:**
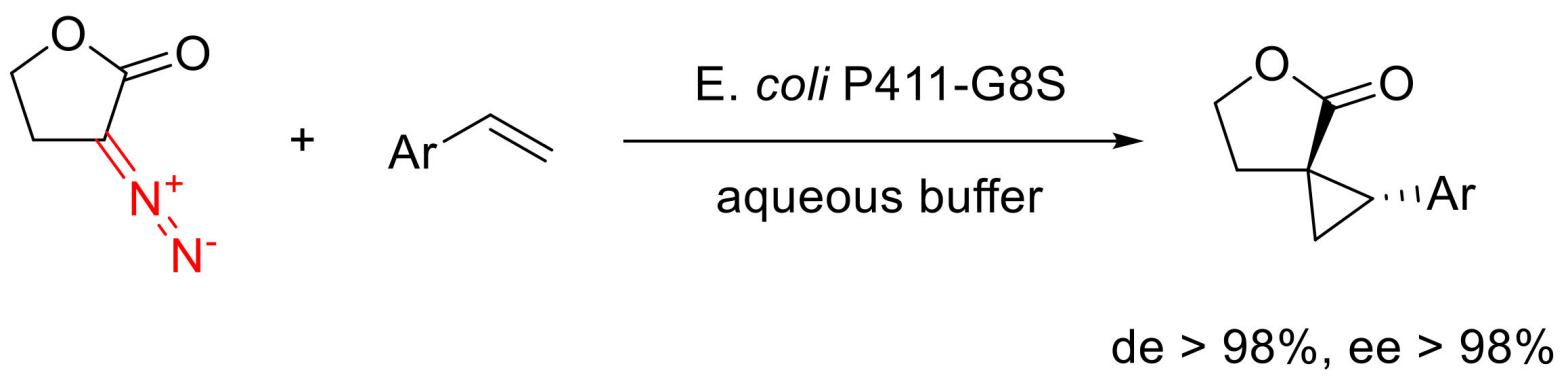
Cytochrome-catalyzed cyclopropanation of styrenes.

α-Diazo-γ-butyrolactones can react with styrenes on catalysis with cytochrome P411, delivering respective cyclopropanes with very high dia- and enantioselectivity (Scheme 89) [119].

**Scheme 90:**
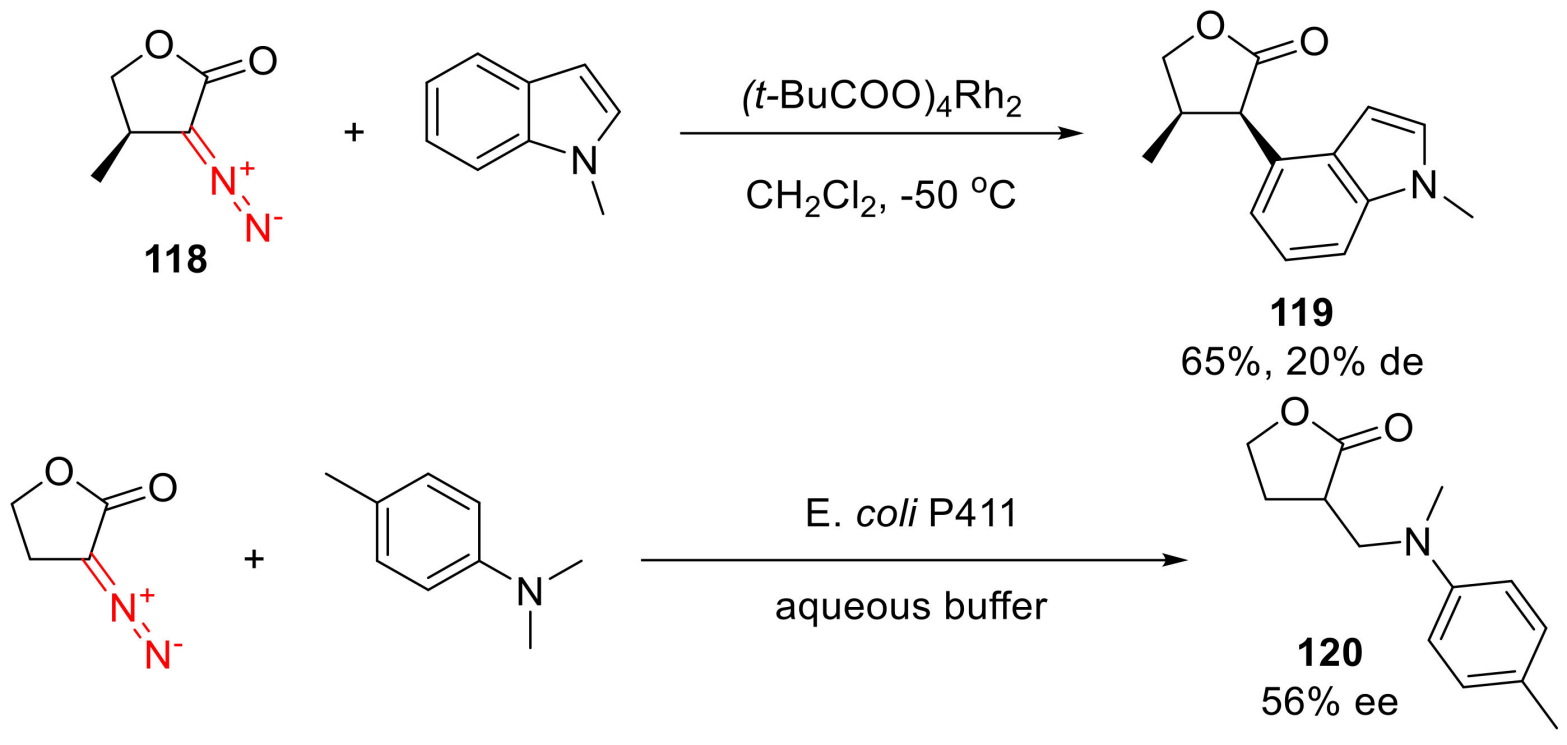
C-H insertion reactions of α-diazo-γ-butyrolactones.

Intramolecular C-H insertion reactions of α-diazo lactones are only sporadically encountered in the literature. For instance, an α-diazo-γ-butyrolactone **118** in the presence of a Rh(II) catalyst gives a C-H insertion product **119** with *N*-methylindole [5], while a cytochrome P411 catalyzed reaction of an α-diazo-γ-butyrolactone delivers enantioenriched product **120** with *N*,*N*-dimethyl-*p*-toluidine (Scheme 90) [123].

**Scheme 91:**
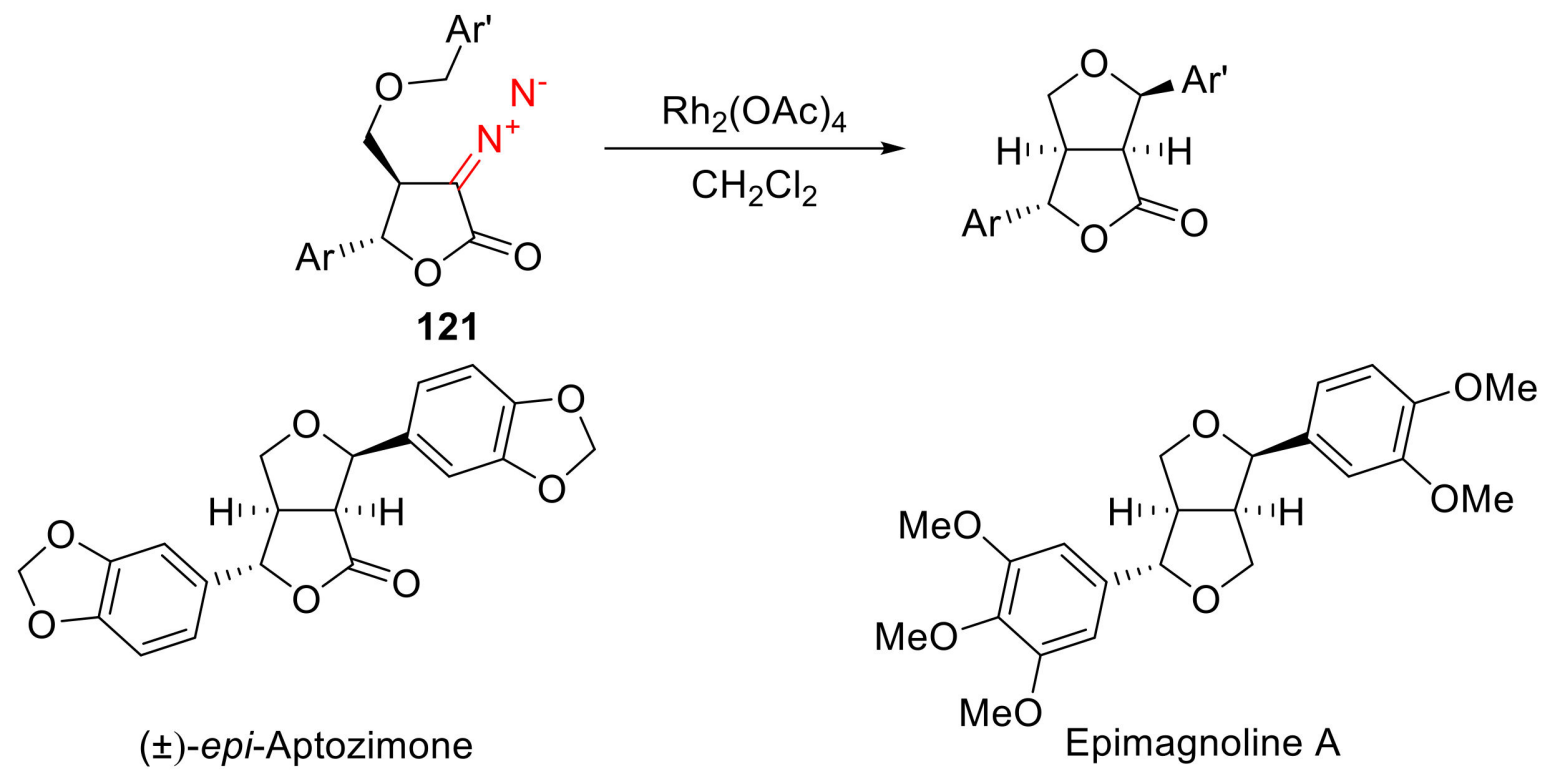
Intramolecular C-H insertion in the synthesis of naturally occurring furofuranones.

The intramolecular C-H insertion reaction of α-diazo-γ-butyrolactones was studied in detail in connection with its use in the synthesis of furofuranoids. C-H insertion involving the sidechain benzyloxy group of α-diazo-γ-aryl-γ-butyrolactones **121** in the presence of Rh(II) acetate is diastereoselective, in all cases, to give an *endo*,*exo*-furofuranone scaffold. This variant of intramolecular C-H insertion was successfully employed in the synthesis of natural furofuranone (±)-*epi*-aptozimone (Scheme 91) [106]. Later, a convenient method of two-step conversion of the resulting lactones into furans was used, which allowed the synthesizing of a series of natural furofuranone lignanes [105,109,112,124].

**Scheme 92:**
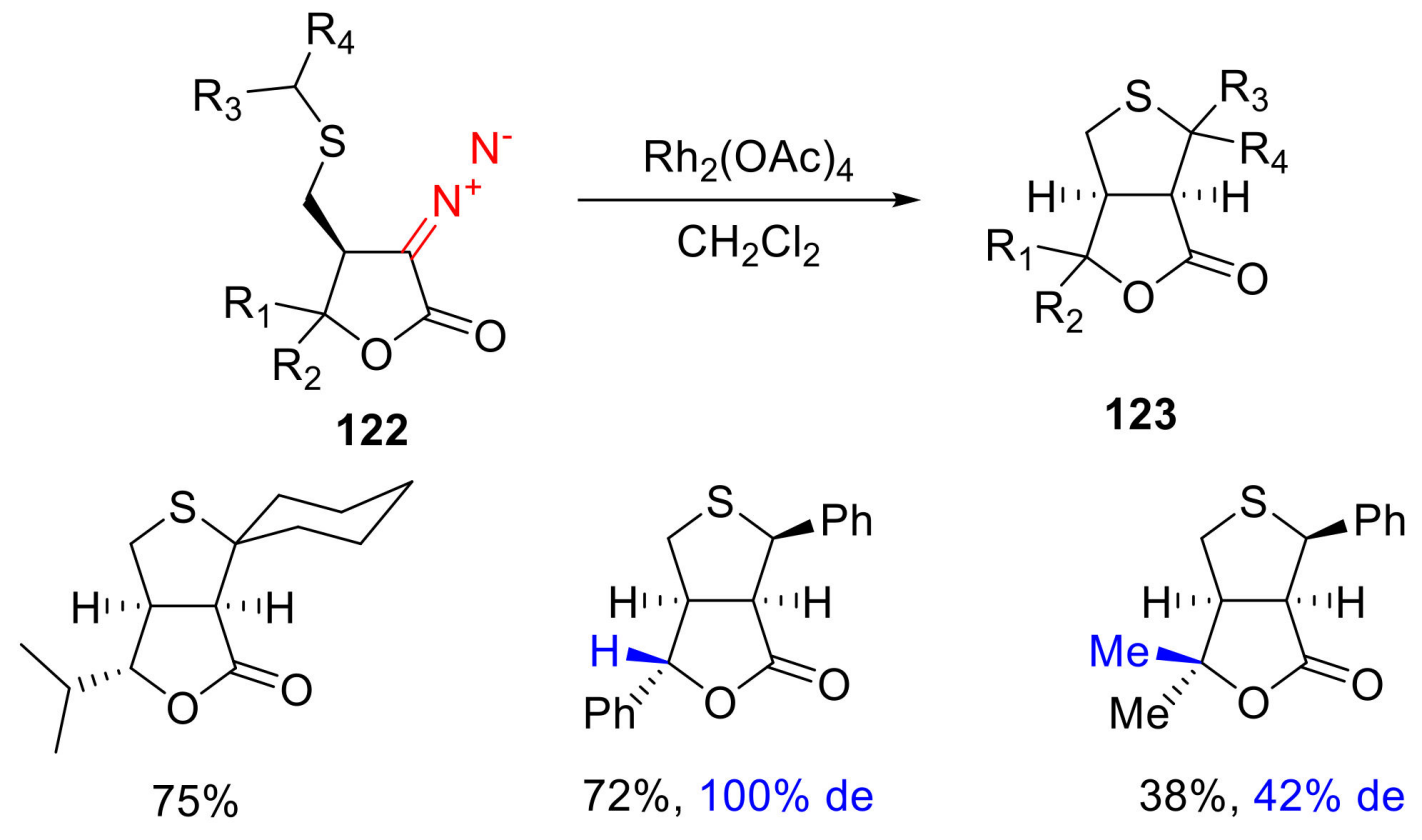
Bicyclic thiofuranone synthesis.

Intramolecular C-H insertion into the benzylthio group of α-diazo-γ-butyrolactones **122**, in most cases, proceeds diastereoselectively and with good yields, delivering thiofuranone bicyclic compounds **123** (including spirocyclic ones) (Scheme 92) [111].

Notably, the outcome of these reactions is dependent on the γ-substitution of the starting lactam. For *cis*-orientation of β- and γ-substituents, the yield drops significantly, and a lower diastereoselectivity is observed. This can be rationalized by the destabilizing interactions in the transition state that lead to the *endo*,*exo*-product.

**Scheme 93:**
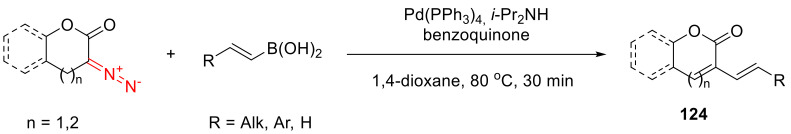
Oxidative cross-coupling with *trans*-vinyl boronic acids.

Similar to their keto-analogs, six- and seven-membered α-diazo lactones can enter an oxidative cross-coupling reaction with *trans*-vinyl boronic acids, delivering the respective α,β-unsaturated products **124** (Scheme 93) [111].

## 4. α-Diazo Lactams

### 4.1. Methods of Synthesis of α-Diazo Lactams

**Scheme 94:**
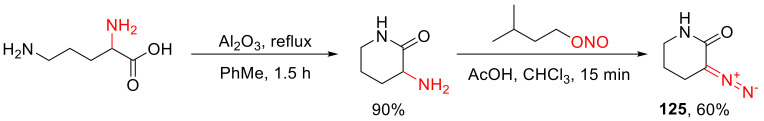
Example of preparation of α-diazo lactams via the nitrozation of an α-amino lactam.

Until recently, the only practical method of preparing α-diazo lactams was the nitrozation of the respective α-amino lactams. This approach allowed the preparation of five- and six-membered lactams (exemplified for the six-membered version **125** in Scheme 94) [125,126].

**Scheme 95:**
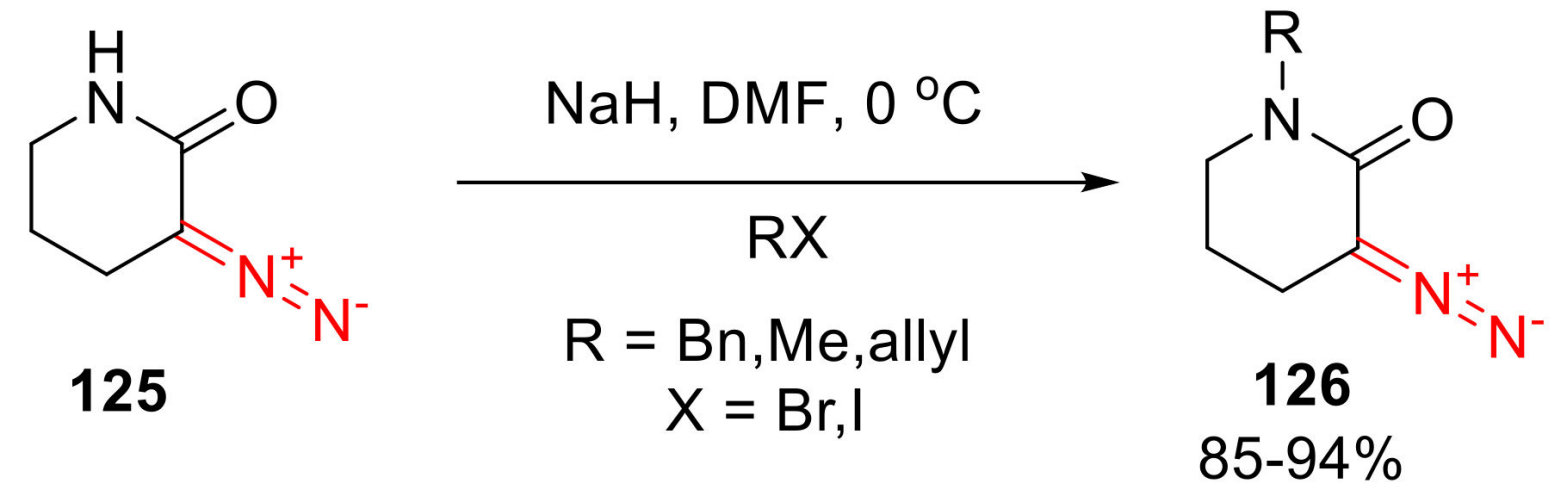
*N*-Alkylation of unsubstituted six-membered α-diazo lactam **125**.

In this case, the synthesis of α-amino lactams is typically conducted from naturally occurring amino acid precursors, which leads to *N*-unsubstituted α-amino lactams. To access their *N*-substituted versions **126**, alkylation or acylation at the lactam nitrogen atom is typically employed (Scheme 95) [127].

**Scheme 96:**
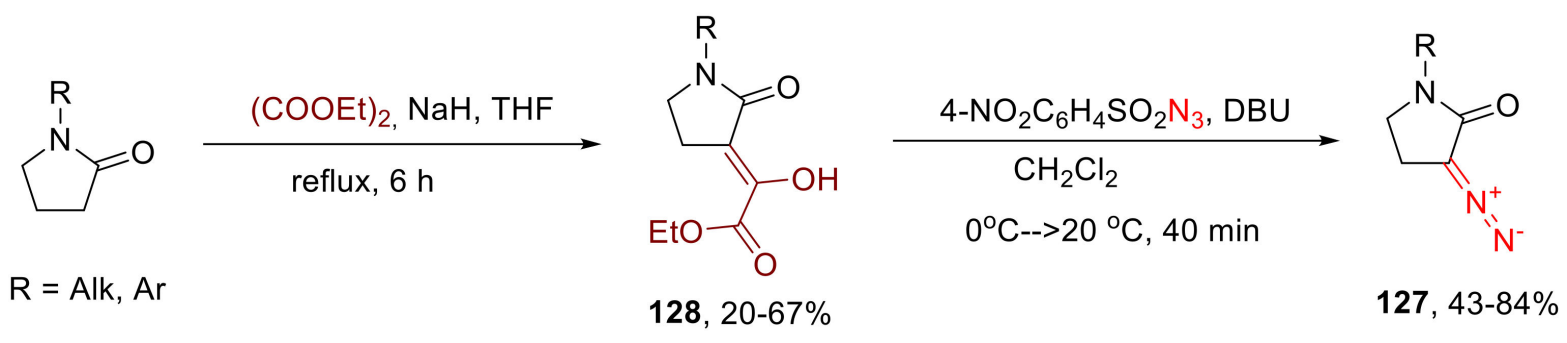
Synthesis of α-diazo-γ-butyrolactams **127** via diazo transfer onto ethoxalylated derivatives **128**.

A more common approach to the synthesis of five-membered α-diazo lactams became the so-called Danheiser diazo transfer. Notably, the classical pre-activation of a lactam substrate via trifluoroacetylation had been studied for only one substrate and was not very successful [5]. Later, it was shown that diversely substituted α-diazo-γ-butyrolactams **127** can be accessed via the diazo transfer onto ethoxalylated substrates **128** (Scheme 96) [128].

**Scheme 97:**
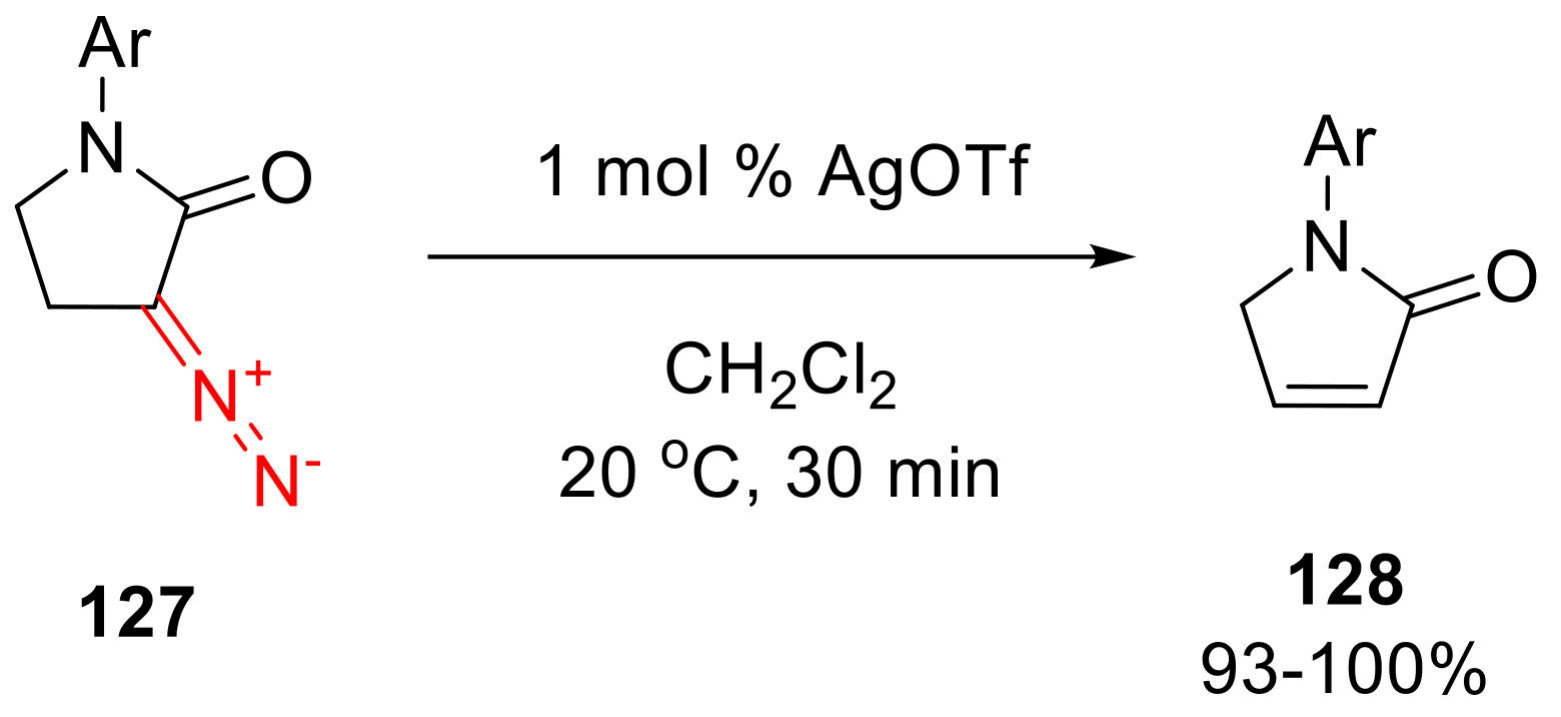
AgOTf catalyzed decomposition of α-diazo-γ-butyrolactams **127**.

To purify diazo compounds **127**, chromatography on neutral alumina has to be used, since they have been found to be unstable with chromatography on silica, eliminating the nitrogen molecule to give 3-pyrrolen-2-one **128**. The latter presumably is formed as the result of the diazo function decomposition and a subsequent 1,2-proton migration. This transformation has been reproduced quantitatively, using only 1 mol% of Lewis acidic silver(I) triflate (Scheme 97) [128].

**Scheme 98:**
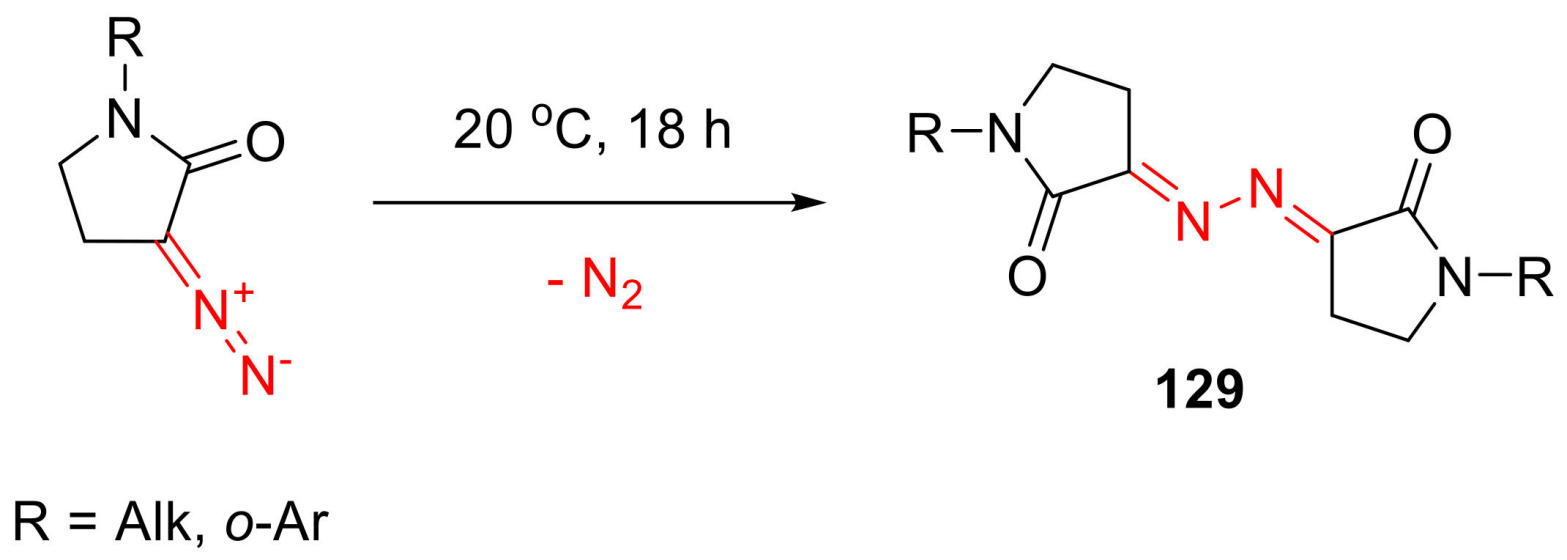
‘Dimerization’ of *N*-alkyl or *N*-(*o*-aryl) α-diazo-γ-butyrolactams.

α-Diazo-γ-lactams bearing alkyl, as well as *o*-substituted aryl groups on the nitrogen atom, have turned out to be particularly unstable. On standing, they rapidly ‘dimerize’ (with the extrusion of a nitrogen molecule) into the respective bis-hydrazones **129** (Scheme 98). Greater stability of *N*-aryl versions (**127**) can be rationalized by the greater stabilization of the diazo function by the aryl groups due to conjugation. *o*-Substitution in the aryl group perturbs that conjugation and makes such systems more ‘*N*-alkyl-like’ [128]. In order to be able to use such unstable α-diazo-γ-butyrolactams in subsequent transformations, a convenient, one-pot protocol has been developed that does not require the isolation of the diazo compounds in the interim [129].

**Scheme 99:**
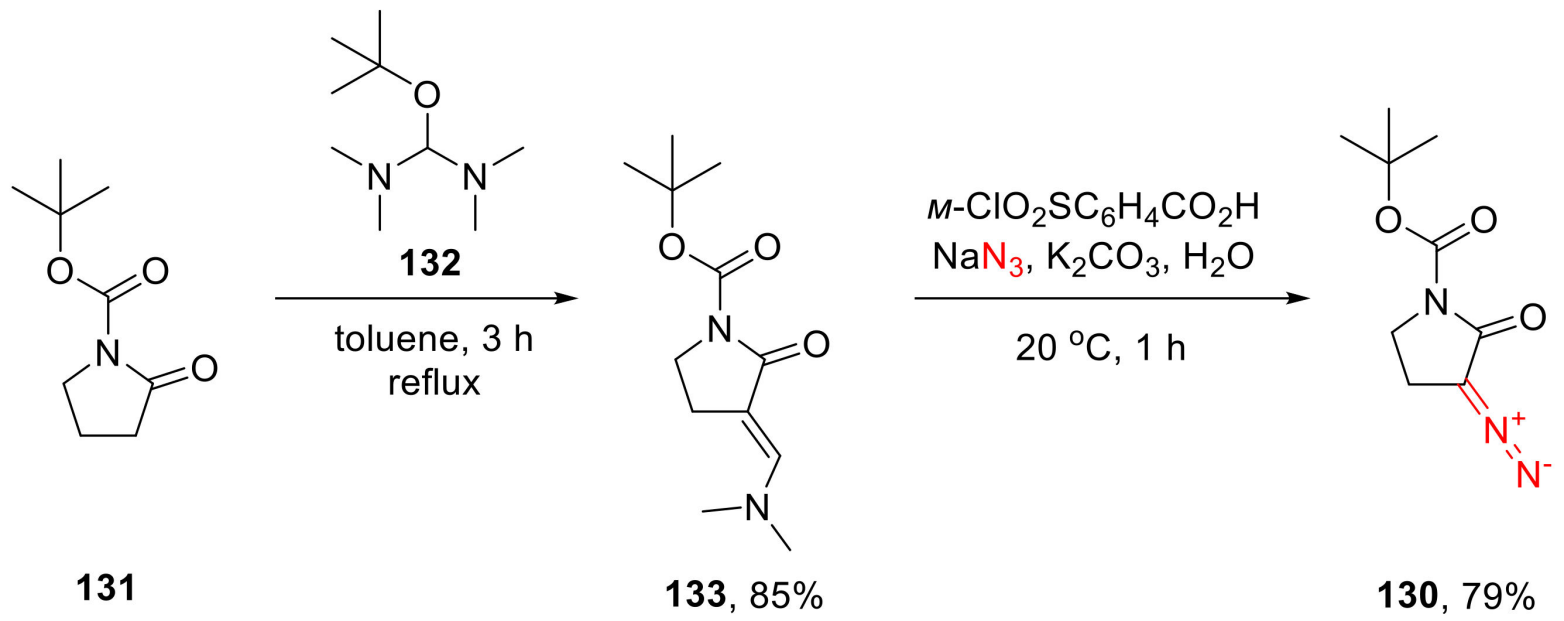
Diazo transfer onto *N*-Boc α-diazo-γ-butyrolactams **131** via α-dimethylaminomethylenation using the Broderick’s reagent (**132**).

As a chemical equivalent of NH-α-diazo-γ-butyrolactams, an *N*-Boc-protected derivative **130** has been developed. Due to the *N*-Boc group in the precursor **131** being chemically labile, the latter could not be activated by base-promoted acylation. Instead, **131** was activated by α-dimethylaminomethylenation using the Broderick’s reagent (**132**), and such derivative (**133**) was subjected to a ‘SAFE’ diazo transfer (Scheme 99) [130].

**Scheme 100:**
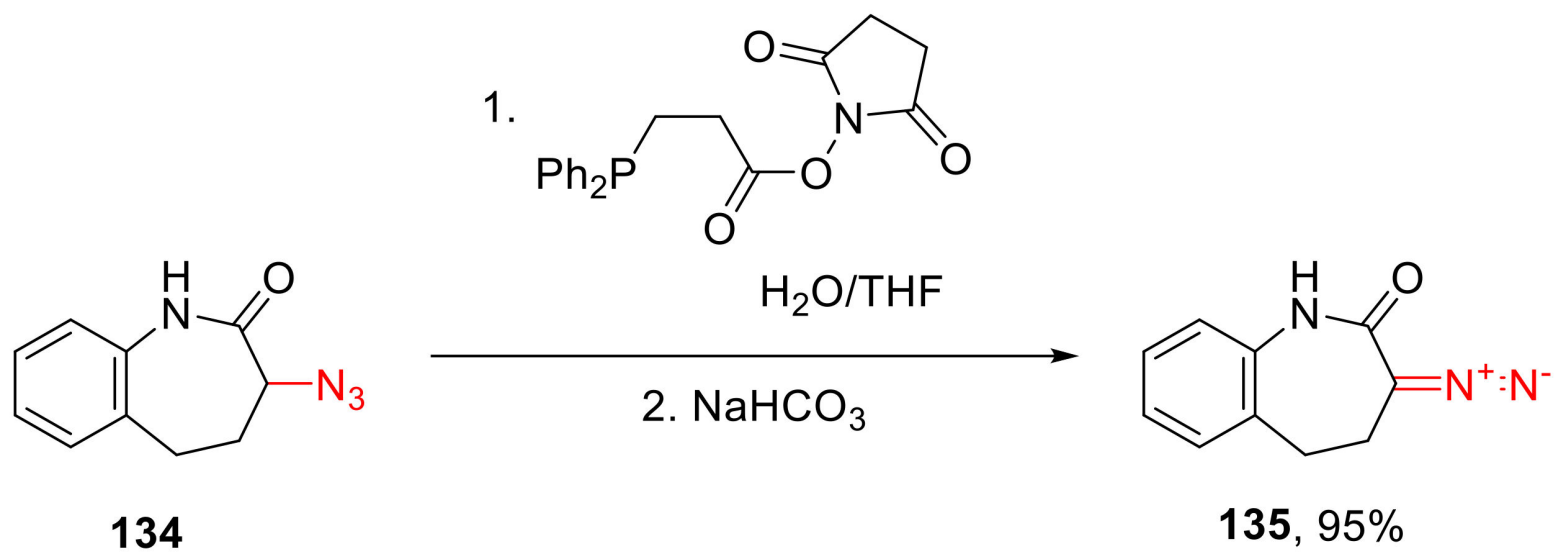
Reduction of an azido lactam with an NHS-containing phosphine.

Just like other diazo compounds, α-diazo lactams can be prepared via the treatment of the respective azido derivatives with a phosphine containing an *N*-hydroxy succinimide (NHS) group. There seems to be only one example of that strategy applied to an azido compound **134**, whereby an α-azido lactam **135** was obtained (Scheme 100). We believe that the scarcity of such examples in the literature has to do with the scarcity of the starting α-azido lactams, as well as with the high cost of the NHS-containing phosphine reagent [116].

### 4.2. Reactions of α-Diazo Lactams

**Scheme 101:**
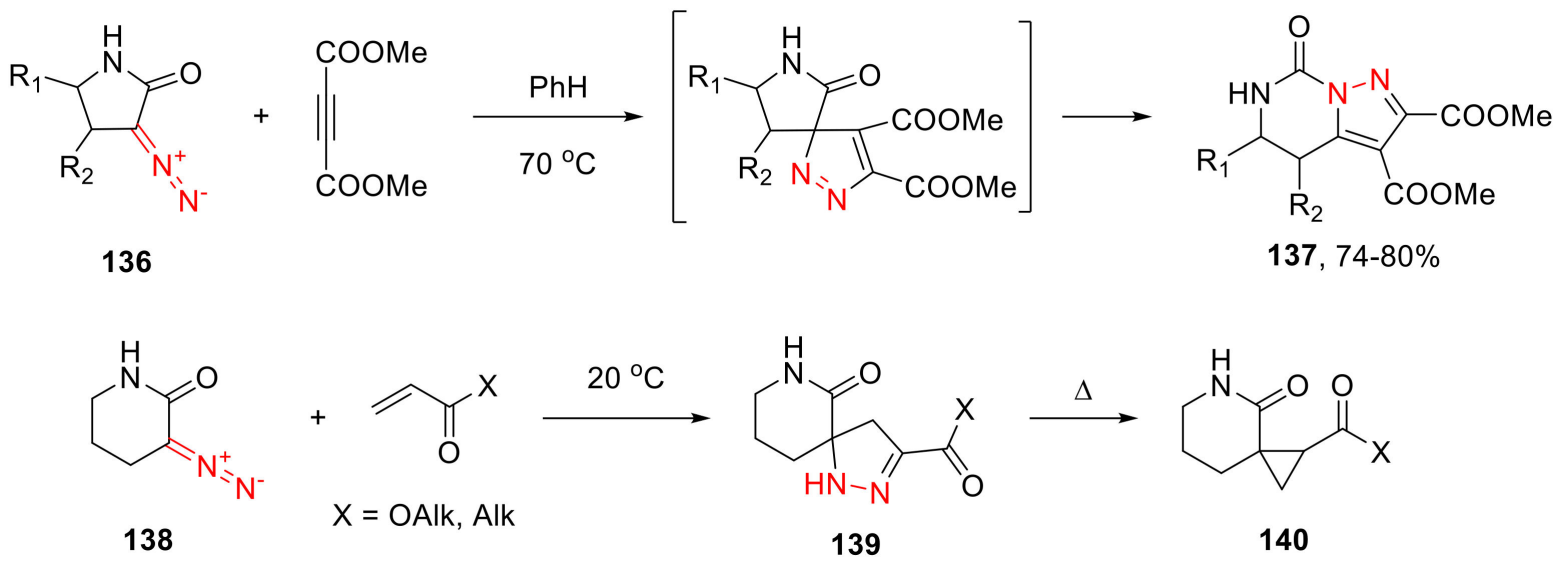
Reactions of α-diazo lactams with alkynes and α,β-unsaturated carbonyl compounds.

α-Diazo lactams, similar to cyclic α-diazo ketones, can enter cycloaddition reactions without the elimination of a nitrogen molecule. The reaction of five-membered α-diazo lactams **136** with dimethyl acetylenedicarboxylate leads to the respective pyrazoles **137**, just as with five-membered α-diazo ketones (vide supra) [126]. In turn, α-diazo-δ-valerolactams, such as **138**, react with α,β-unsaturated carbonyl compounds, delivering spiro pyrazolinones **139**, which, under thermal conditions, lose a nitrogen molecule, turning themselves into spiro cyclopropanes **140** (Scheme 101) [125].

Similar to other cyclic α-diazocarbonyl compounds, α-diazo lactams can enter various reactions of formal insertion into the X-H bond, exemplified by such reactions as the hydrochlorination of 3-diazopyperidine-2-one in refluxing 1,4-dioxane [125] and the hydrofluorination of α-diazo-γ-butyrolactams by the Olah’s reagent [131].

**Scheme 102:**
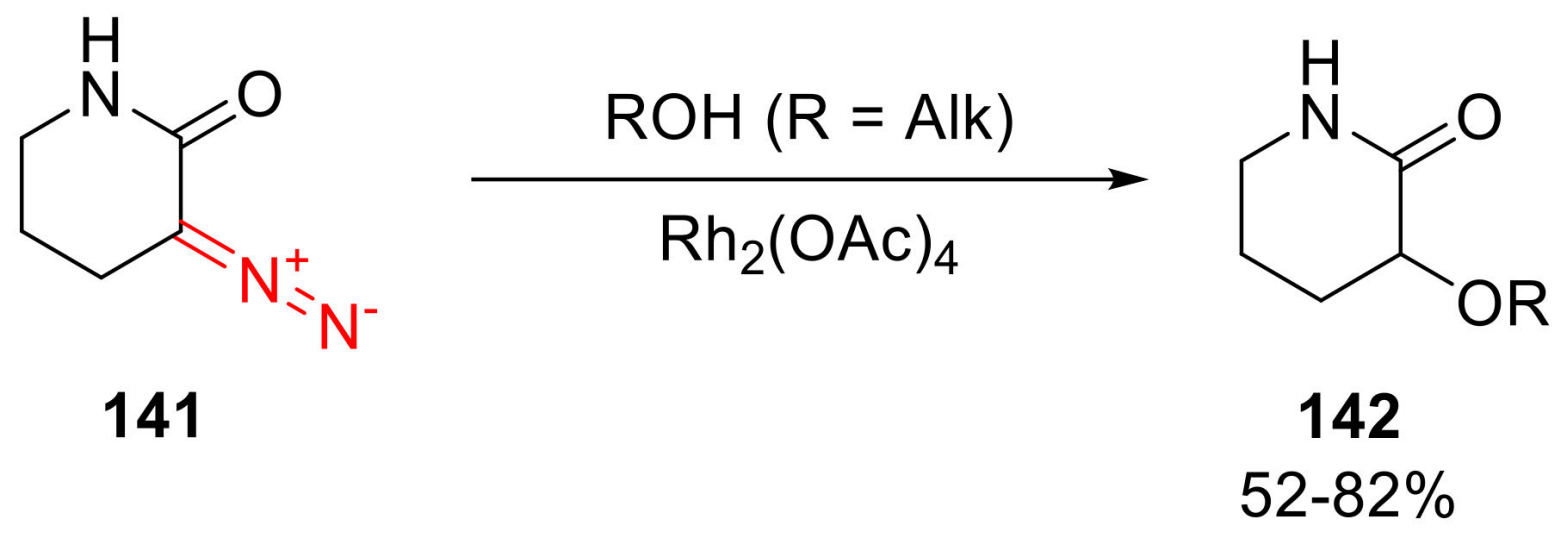
Rh(II)-catalyzed insertion of 3-diazopiperidine-2-one into the O-H bond of alcohols.

The O-H insertion reaction of alcohols has been studied well for α-diazo-δ-valerolactams, such as **141**. The decomposition of 3-diazopiperidine-2-one (**141**) in the presence of Rh(II) acetate in an alcohol used as a solvent delivers 3-alkoxypiperidine-2-ones **142** (Scheme 102). The reaction turns out to be sensitive to the sterics of the alcohol reactant—going from methanol to *tert*-butanol, the yield drops more than 1.5-fold [125].

Later, the reaction of formal insertion into the O-H bond of alcohols and water was studied for α-diazo-γ-butyrolactams [5,128,130]. For the five-membered α-diazo lactams, the transformation turns out to be even more sensitive to the steric bulk of the reacting alcohol. In particular, *tert*-butanol gives no product at all in this reaction.

**Scheme 103:**
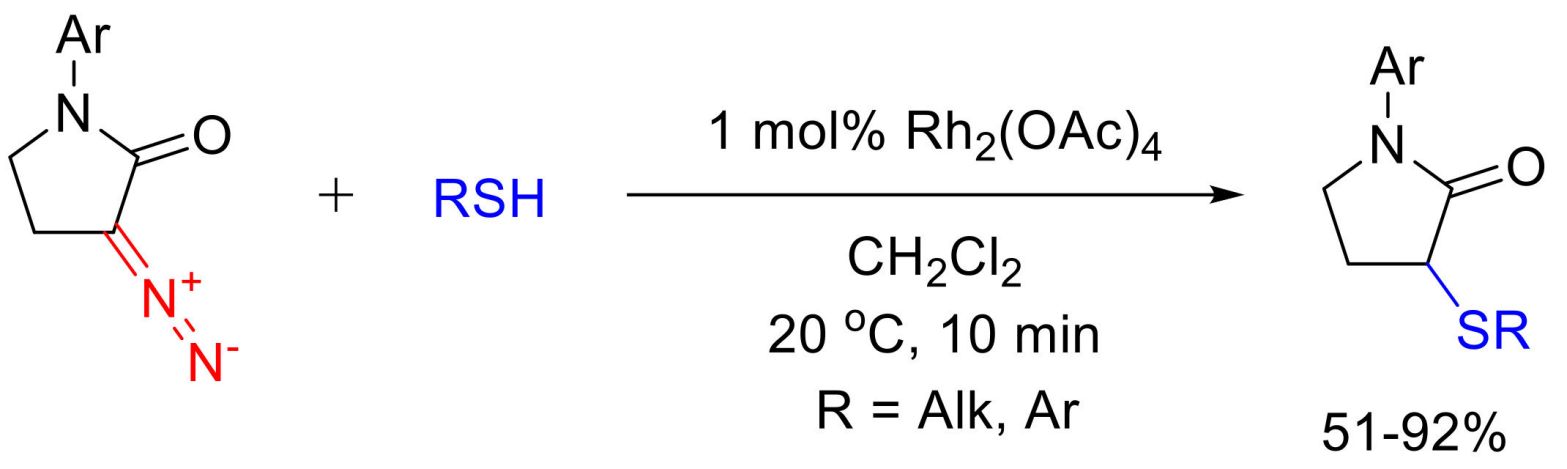
S-H insertion reaction of α-diazo-γ-butyrolactams under Rh(II) catalysis.

α-Diazo-γ-butyrolactams, when decomposed under Rh(II) catalysis, react well in the reaction of the formal S-H insertion (Scheme 103). Interestingly, in contrast to the O-H insertion reaction, this transformation has been found to be less sensitive to the sterical properties of the reacting thiol: even *tert*-butyl thiol gives a respectable 63% yield in this reaction. This observation can be rationalized by the longer C-S bond in thiols compared to the C-O bond in alcohols [130,132]. A few successful examples of S-H insertion reactions have also been reported for six-membered α-diazo lactams [133].

The S-H insertion reaction of α-diazo-γ-butyrolactams as the means of desymmetrizing symmetrical dithiols (ethane-1,2-dithiol and propane-1,3-dithiol) was later reported as an efficient strategy in constructing novel metallo-β-lactamase inhibitors [134].

**Scheme 104:**
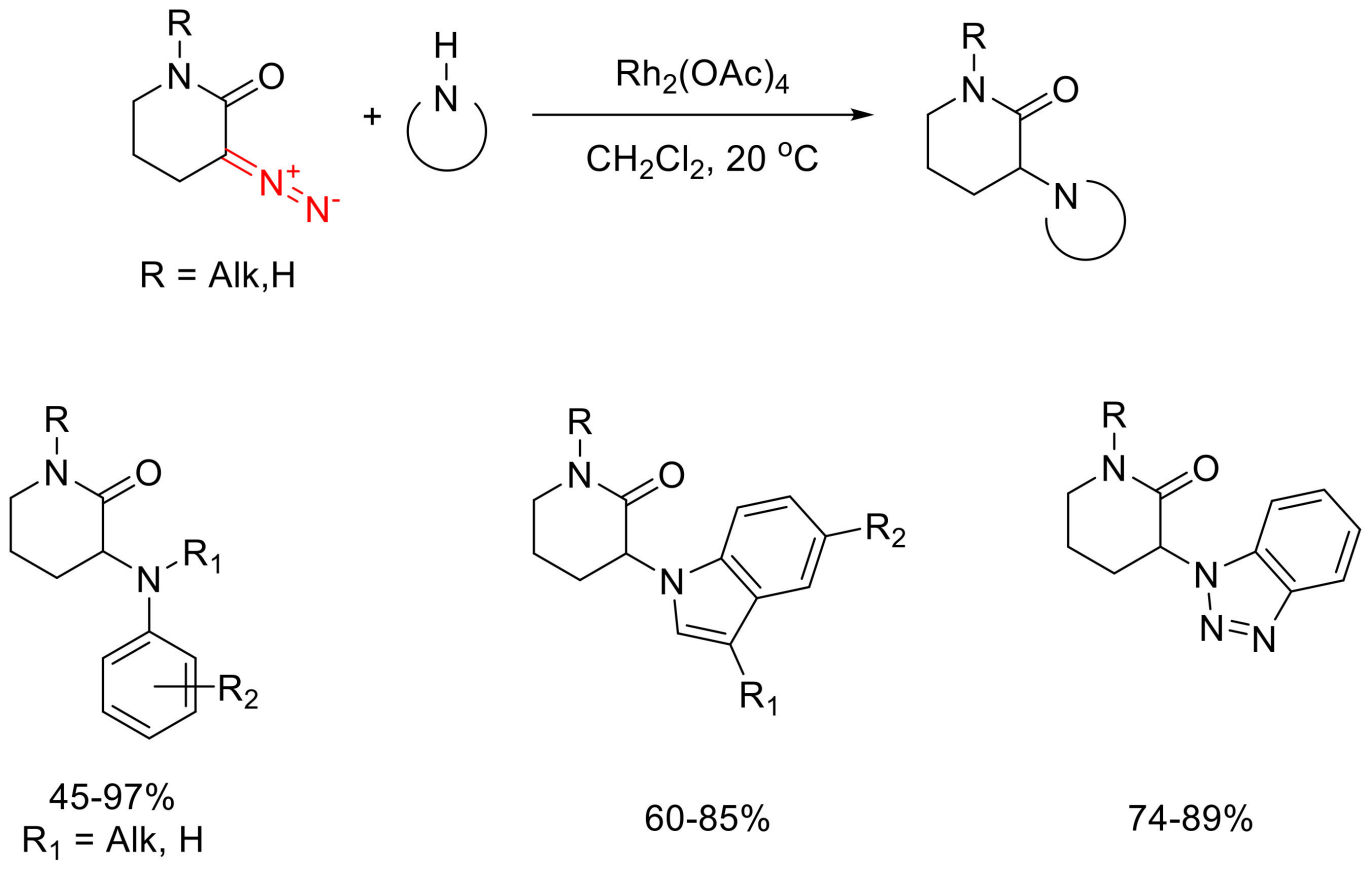
Rh(II)-catalyzed N-H insertion reactions of α-diazo-δ-valerolactams.

Six- and five-membered α-diazo lactams are also capable of entering a formal N-H insertion reaction with aromatic amines under Rh(II) acetate catalysis. The reaction has been shown to be sensitive to the electronic properties of the reacting aniline: the reaction gives better yields with anilines bearing electron-withdrawing substituents. Based on this observation, it has been concluded that the reaction proceeds as a concerted process rather than being mediated by the formation of an ammonium ylide (in which case, more electron-rich anilines would produce better results). It is interesting to note the selectivity of the N-H insertion reaction over O-H insertion: the reaction with *o*-amino phenol gives absolutely no *O*-alkylation product. In the case of α-diazo-δ-valerolactams, the N-H insertion reaction is reported not only for anilines but also for such N-H heterocycles as benzotriazole and indole (Scheme 104). Unfortunately, the scope of heterocycles that can be alkylated through such a process is limited. For instance, an attempt to involve imidazole or benzimidazole in this reaction did not even lead to the decomposition of the starting diazo lactam, presumably, due to the poisoning of the Rh(II) catalyst by the coordinating heterocycles. The selectivity of the N-H insertion into external amines and heterocycles over intramolecular N-H insertion into the valerolactams themselves [130,135,136] is also notable in these reactions.

**Scheme 105:**
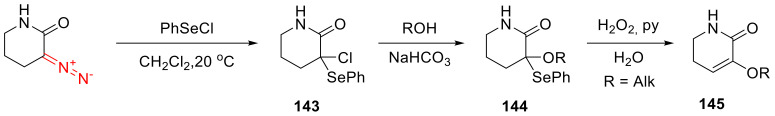
Evolution of 3-diazopiperidin-2-one via mild chloroselenylation.

3-Diazopiperidin-2-one can also be chloroselenylated under mild conditions. The initial chloride **143** can be further converted to alkoxide **144**, whereupon the phenylselenyl group can be removed under oxidative conditions to give α,β-unsaturated product **145** (Scheme 105) [137].

**Scheme 106:**
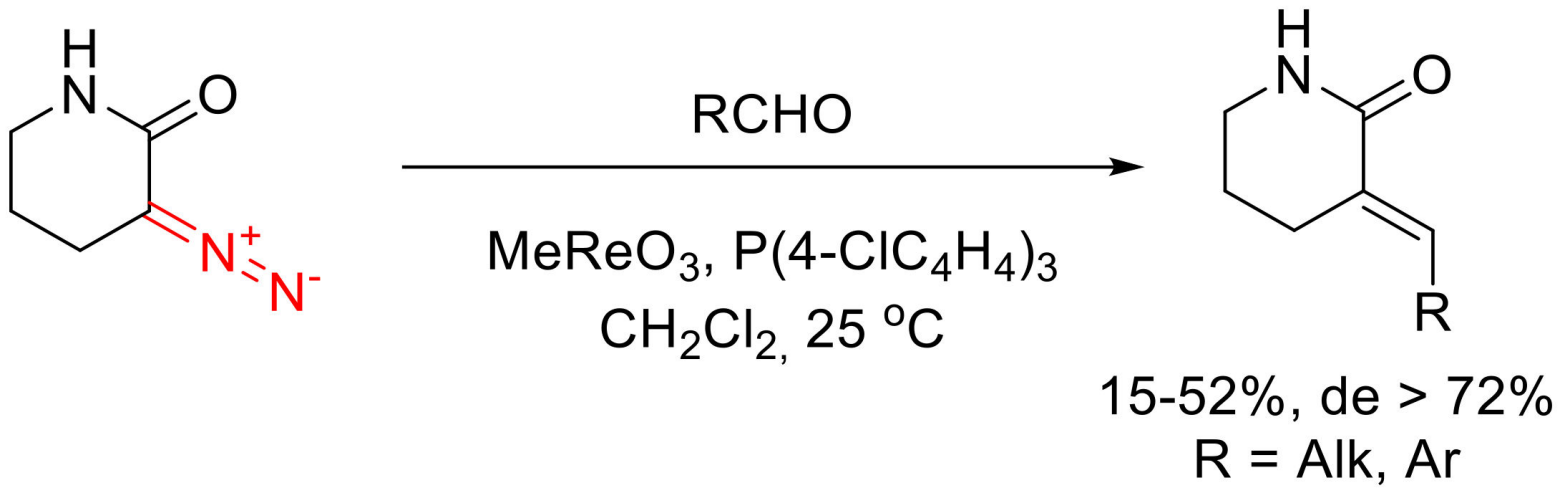
Olefination of α-diazo-δ-valerolactams with aldehydes.

α-Diazo-δ-valerolactams can be olefinated with aldehydes in the presence of trioxomethyl rhenium and triaryl phosphine (Scheme 106). The diastereoselectivity of the reaction has been shown to be dependent on the structure of the phosphine. The best results are obtained with tris(4-chlorophenyl)phosphine, which gives predominantly (*E*)-configured olefins. Unfortunately, the yields remain rather modest throughout [125,138].

**Scheme 107:**
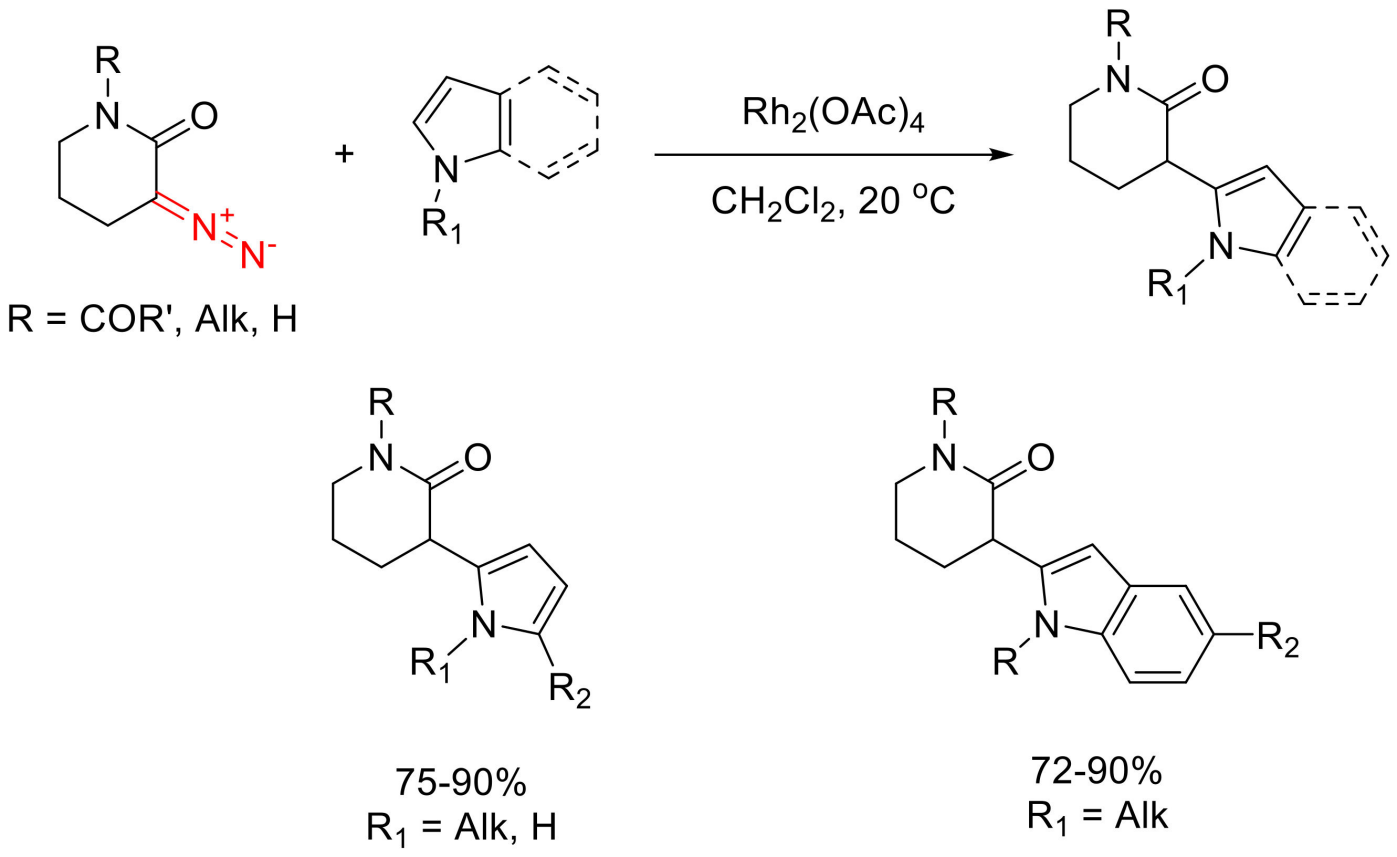
Rh(II)-catalyzed C-H insertion reaction of pyrroles and indoles.

Six-membered α-diazo lactams are also capable of insertion into the C-H bond of indoles and pyrroles under Rh(II) catalysis (Scheme 107). The transformation gives good yields for *N*-alkyl azoles, while putting an electron-accepting group (acyl or tosyl) on the pyrrole nitrogen atom renders the reaction completely ineffective. This can be rationalized by the reaction proceeding through the formation of a zwitter-ionic intermediate, in which the positive charge is localized on the azole ring [127]. Additionally, the following difference between pyrrole and indole substrates is noteworthy. *N*-Unsubstituted pyrroles enter the C-alkylation reaction quite readily, while *N*-unsubstituted indoles primarily give N-H insertion products [127].

**Scheme 108:**
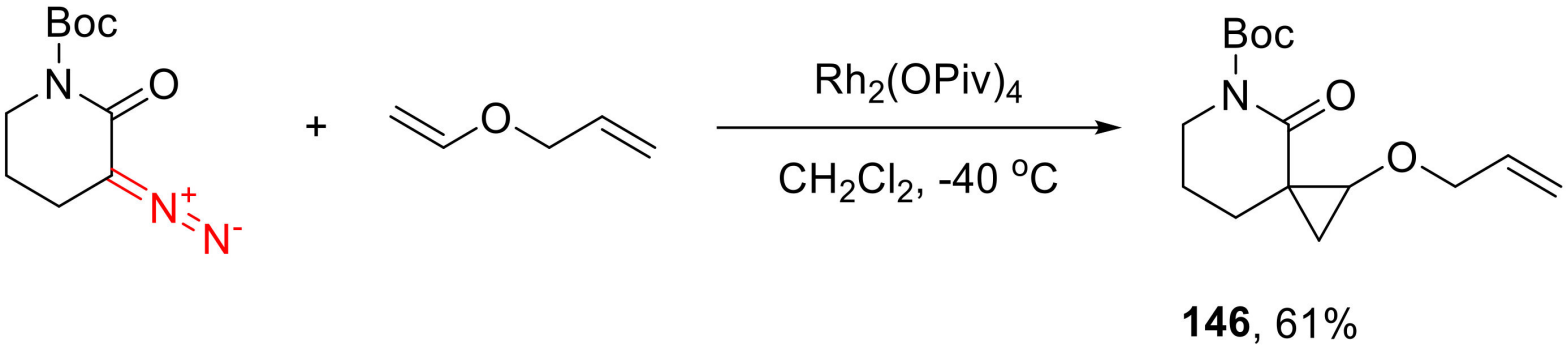
Selective cyclopropanation of allyl vinyl ether.

Cyclopropanation of α-diazo lactams is featured in the literature only fragmentarily. Six-membered substrates can react with styrenes, enol ethers and dihydrofuran in the presence of Rh(II) acetate [125]. Unfortunately, the reaction is not always diastereoselective. Notably, electron-rich olefins are more readily cyclopropanated, which is illustrated by the formation of a sole enol ether cyclopropanation product **146** in the reaction of a Boc-protected α-diazo-δ-valerolactam with allyl vinyl ether (Scheme 108) [139]. There is only a single example of the cyclopropanation of styrene by a five-membered α-diazo lactam in the literature [5].

**Scheme 109:**
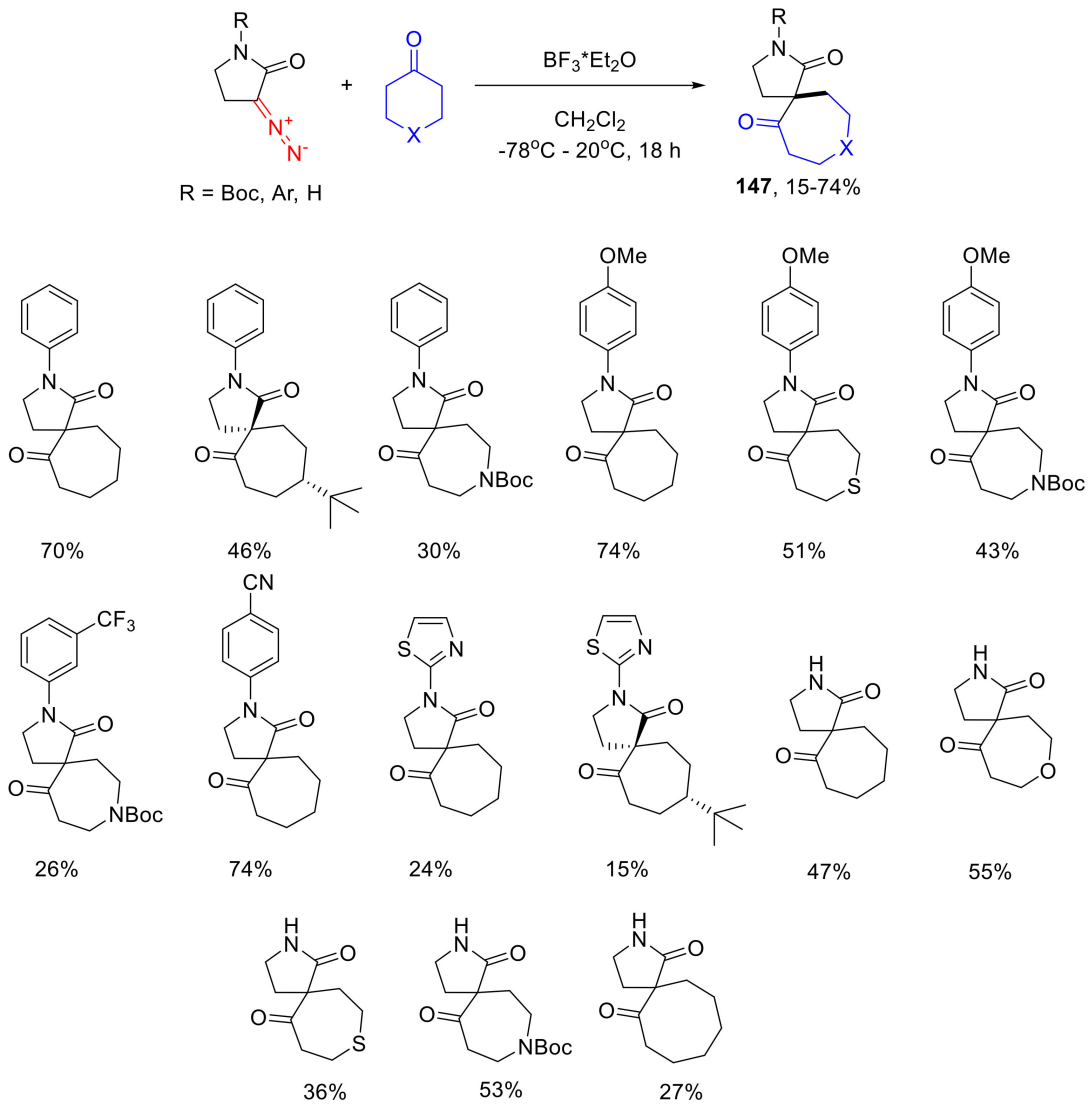
The Büchner–Curtius–Schlotterbeck reaction of α-diazo-γ-butyrolactams with cyclic ketones.

α-Diazo-γ-butyrolactams, similarly to α-diazo-γ-butyrolactone, can enter the Büchner–Curtius–Schlotterbeck reaction with cyclic ketones. It has been established that the best results in this reaction can be achieved at a low temperature and by using an equivalent amount of boron trifluoride diethyl etherate as the Lewis acid promoter of the reaction. Interestingly, no spirocyclic product **147** is obtained with Sc(III) triflate as the catalyst, while for α-diazo-γ-butyrolactones, this catalyst works well. Altogether, the reaction works well for six- and seven-membered cyclic ketones but, surprisingly, not for cyclopentanone. Interestingly, when using an *N*-Boc-protected α-diazo-γ-lactam, the Boc group is removed in the course of the spirocycle formation (Scheme 109) [140].

## 5. Conclusions

As it follows from the material presented above, cyclic α-diazocarbonyl compounds differ from their acyclic counterparts in their chemical properties. The peculiarities of their reactivity have found ample utility in modern synthetic organic chemistry.

At the same time, although different classes of cyclic α-diazocarbonyl compounds are studied equally well and for certain classes, the typical transformations of diazo compounds have been described only fragmentarily in the literature. Specifically, this is true for α-diazo lactones and lactams.

The starting materials for the synthesis of α-diazo lactones are not readily available, which has limited the study of this class of reagents to the simplest, unsubstituted representatives of rather specific examples. Moreover, the chemical transformations of α-diazo lactones described to-date are not particularly diverse, presumably, due to the limited chemical stability of α-diazo lactones themselves.

The situation with α-diazo lactams is more complex and more interesting. Until very recently (2019), the approach to the synthesis of their five-membered variants (α-diazo-γ-butyrolactams) was limited to only one, not very advantageous example, which was limiting the study of this class of reagents. At the same time, the chemical stability of available α-diazo-δ-valerolactams was well-established and, hence, a wider range of their chemical transformations has been described in the literature. In contrast, five-membered α-diazo lactams turned out to be much less studied, and this drawback has been addressed only in the last 2–3 years in our laboratories.

## Data Availability

Not applicable.
